# Propedia v2.3: A novel representation approach for the peptide-protein interaction database using graph-based structural signatures

**DOI:** 10.3389/fbinf.2023.1103103

**Published:** 2023-02-16

**Authors:** Pedro Martins, Diego Mariano, Frederico Chaves Carvalho, Luana Luiza Bastos, Lucas Moraes, Vivian Paixão, Raquel Cardoso de Melo-Minardi

**Affiliations:** Laboratory of Bioinformatics and Systems (LBS), Department of Computer Science, Universidade Federal de Minas Gerais, Belo Horizonte, Minas Gerais, Brazil

**Keywords:** propedia, peptide, protein, interactions, graph-based, database

## 1 Introduction

Propedia is a web database of peptide-protein interactions, which introduced a clustering approach based on three methods: (i) sequences, (ii) structure interface, and (iii) binding sites. Developed between the years 2015–2020, the first version of Propedia presented a total of 19,813 complexes peptide-protein, grouped by 1,845 sequence clusters, 1,891 interface clusters, and 1,466 binding site clusters. Here we show the first large-scale Propedia update: version 2.3. Propedia v2.3 presents over 49,300 peptide-protein complexes (an increment of approximately 150%) and introduces a new representative approach developed using graph-based structural signatures. In this data report, we also describe how the structural signatures of peptides from Propedia were calculated using the aCSM-ALL algorithm. We performed two case studies using seven machine learning algorithms to evaluate these new Propedia resources: (i) clustering based on sequences; and (ii) clustering based on six Propedia sub-dataset determined from PDB’s classification. Our analyses demonstrated that graph-based signatures could be useful in classifying peptides. Propedia v2.3 is available at http://bioinfo.dcc.ufmg.br/propedia2/.

### 1.1 Peptides

Peptides are biomolecules containing two to dozens of amino acid residues linked through peptide bonds (Neduva et al., 2005). Peptides represent a unique class of pharmaceutical compounds, molecularly poised between small molecules and proteins yet biochemically and therapeutically distinct from both (Lau and Dunn, 2018). It is estimated that between 15% and 40% of all protein-protein interactions in cells are mediated by these molecules (Angelova et al., 2019).

Nowadays, thousands of naturally occurring peptides have been identified. These often have crucial roles in human physiology, including actions such as hormones, neurotransmitters, growth factors, ion channel ligands, or anti-infectives (Moreno-Camacho et al., 2019). Compared to proteins, peptides are considered more versatile chemically because they can be more easily modified. In addition, they induce low resistance with limited non-target activity, making them good therapeutic agents (Lau and Dunn, 2018; Vinogradov et al., 2019).

Since the synthesis of the first therapeutic peptide, insulin, in 1921, remarkable achievements have been made. Peptide drug development has thus become one of the hottest topics in pharmaceutical research (Lau and Dunn, 2018). Previously, peptide pharmacological use was discouraged by existing limitations such as the short half-life and their low oral bioavailability. However, in recent years, new synthetic approaches have allowed their greater use as drugs, circumventing existing limitations (Wang et al., 2018; Pant et al., 2020). Currently, there are around 80 peptide drugs on the global market, and research for new peptide therapies continues at a steady pace, with over 150 peptides in clinical development and another 400–600 peptides in preclinical studies (Wang et al., 2018).

Therefore, understanding the structure and recognition of protein-peptide complexes can assist in designing new peptides and peptide-based compounds for drug development or biotechnological purposes. In this way, databases of protein-peptide complexes may pave the way for the analysis and understanding of protein-peptide recognition mechanisms (London et al., 2010; Das et al., 2013).

### 1.2 Propedia v.1.0

Previously, we proposed Propedia: a database of peptide-proteins interactions (Martins et al., 2021). Propedia is a comprehensive and up-to-date database with a web interface that allows you to group, search, and visualize the peptide-protein according to combinations. To provide a comprehensive dataset of complex protein-peptide experiments, we organized its first version into three types of clusters based on: (1) sequence similarity, (2) structure interface, and (3) potential protein-peptide binding site. These clustering strategies were suggested to analyze structures from different perspectives, aiding in detecting peptides for interaction with a target of interest. In the first version, the Propedia presented 19,813 complexes peptide-protein. At that time, Propedia was the filtered database with the most peptide structures available. However, with the large increase in the number of protein complex structures deposited in public databases, such as the PDB—Protein Data Bank (Bernstein et al., 1978; Berman et al., 2000), we realized the need to update the Propedia database. To update Propedia, we collect data from PDB and parser the 3D structures, separating complexes in peptide-protein pairs. Thus, Propedia creates an entry for each complex protein-peptide. For instance, if a complex has four chains A, B, C, and D interacting (being A and B proteins, and C and D peptides), Propedia generates four entries: A-C, A-D, B-C, and B-D. For each entrance, Propedia calculates several pieces of information that are not shown in PDB. For example, binding area (Å^2^) of protein and peptide, percentual of hydrophobic amino acids in the peptide, molecular weight, aromaticity, instability, isoelectric point, and so on. We also show similar entrances based on sequence comparisons between peptides, contact patterns in protein-peptide complexes, and interface interactions.

Now we present Propedia v2.3, which counts with the update of the number of complexes present in the database of 49,300, besides a code restructuring that allowed better performance and an improvement in the navigability of the web tool. From now on, Propedia will adopt the release year for its versions, i.e., version 2.3 corresponds to the version released at the beginning of 2023, with an expectation of significant updates released between 2 and 3 years. In addition to the new update policy, as justification for this new publication, Propedia v2.3 presents a new representative approach for peptides, developed using structural signatures based on graphs.

### 1.3 Structural signatures

A graph-based structural signature is a numerical representation of a macromolecule 3D structure used to detect similarities and differences between different structures. In this method, each atom is modelled as a graph node. Interactions between atoms can be represented by edges that connect the nodes. Additionally, edges can represent the relationship between an atom pair in a specific cutoff distance. Several studies have pointed out the advantage of using structural signatures to extract characteristics of macromolecules (Pires et al., 2011; Mariano et al., 2019; Rodrigues et al., 2022). For instance, the aCSM approach creates a cumulative vector of pairwise atoms from a cutoff range. This method presents two main steps (Pires et al., 2013).

Step 1 A distance matrix is calculated for the three-dimensional coordinates of every atom of a peptide against the other atoms.

Step 2 Then, it is calculated the number of atom pairs for a range distance, considering eight types of atoms (for the aCSM-all variation). These atom types can be acceptor, donor, aromatic, hydrophobic, negative, neutral, positive, and sulfide. The range distance is defined by two parameters: cutoff step and cutoff limit. For example, for a cutoff step of 1Å and a cutoff limit of 5Å, the signature counts the number of atom pairs at a distance from 0-1Å, 1-2Å, 2-3Å, 3-4Å, and 4-5Å. This results in a numerical vector representing the molecule structure.

The application of this approach for the peptide structures results in a signature vector dataset: a CSV (comma-separated values) file with a set of features that can represent the peptide structure and can be used, for example, in machine learning tasks. For instance, this new dataset could be used for detecting new peptides with therapeutic characteristics, predicting new functional peptide structures, or even inferring new uses for peptides with structures already publicly available.

## 2 Methods

In this section, we describe the strategies used for updating PropediaDB as well as calculating the novel proposed peptide sub-dataset based on structural signatures.

### 2.1 Data collection

Data was collected from Protein Data Bank on 15 November 2022. The criteria used for retrieving PDB entries were: (i) structures with 2–50 amino acids residues length; (ii) the structure should have at least two chains; and (iii) the structure should be solved using X-ray crystallography (resolution >2.5 Å) or by NMR (Nuclear Magnetic Resonance) spectroscopy.

### 2.2 Structures pre-processing

Data pre-processing is performed for each PDB structure collected using in-house Python scripts. For every PDB structure, lines corresponding to atoms of pair of peptide-protein are collected and stored in separated PDB files. Additionally, the contact interface is calculated and stored in corresponding files. Finally, metadata for each related complex is stored in a MySQL databank (these will be used to feed the search engine based on sequences, structures, and binding sites).

### 2.3 Signatures dataset

We obtained the structural signature of each peptide structure from Propedia v2.3 using the Signa molecular signatures toolkit (not published yet). Signa was executed using parameters “signature algorithm: acsm_all”, “cutoff_limit: 20Å”, and “cutoff_step: 0.2 Å”. The aCSM-ALL algorithm generates signatures containing the number of atoms at each distance cutoff in Å for each atom category considered (hydrophobic, positive, negative, acceptor, donor, aromatic, sulfur and neutral).

The final signature dataset is a CSV file in which each line contains the peptide ID (composed of the PDB ID and the peptide chain), followed by a structural signature vector of 3,600 representative numbers. The explanation of these numbers was included in the Supplementary Material.

### 2.4 Machine learning

We performed two case studies to evaluate the use of structural signatures to classify peptide-proteins structures of Propedia: (1) classifying based on sequence clusters (s0, s1, s112, s151, and s162); and (2) classifying based on the six specific Propedia sub-datasets: AntimicrobialDB, ViralDB, EnzymeDB, MembraneDB, HormoneDB, and PlantDB. The first dataset was obtained from a case study based on sequences similarities performed on the first Propedia version. On the other hand, the second case study includes a dataset collected from PDB metadata classifying based on the complexes’ characteristics (details will be presented in the next section). To evaluate if the signatures proposed were correctly describing the peptide groups, we proposed an analysis based on machine learning using Orange Data Mining (https://orangedatamining.com/).

Models were constructed using seven machine learning algorithms in the Orange Data Mining tool (default parameters were used in all case studies). First, SVM was executed using: cost (c = 1.00), regression loss epsilon of 0.10, linear kernel, numerical tolerance equal to 0.0010, and interaction limit of 100. Next, KNN was executed using the parameters: number of neighbours equal to 3, metric “Manhattan”, and weight “Distance”. Third, the neural network algorithm was performed using the parameters: Neurons in hidden layers equal to “300”, activation “ReLu”, solver “Adam”, regularization *α* = 0.001, the maximal number of iterations 200, and replicable training. Gradient Boosting was performed using the method “Gradient Boosting (scikit-learn)” and the number of trees equal to “100”, learning rate “0.100”, replicable training, growth control for limit depth of individual trees of “3” and “do not split subsets smaller than 2”, and the fraction of training instances of “1.00”. Logistic Regression was performed using the regularization type of “ridge (L2)” and strength c = 1. The decision tree algorithm was performed using the parameters “induced binary tree”, the minimum number of instances in leaves equal to “2”, do not split subsets smaller than “5”, maximal tree depth “100”, and classification stops when majority reaches 95%. Lastly, the random forest was performed using ten trees and the minimum length of subsets equal to 5.

## 3 Analysis

**FIGURE 1:**
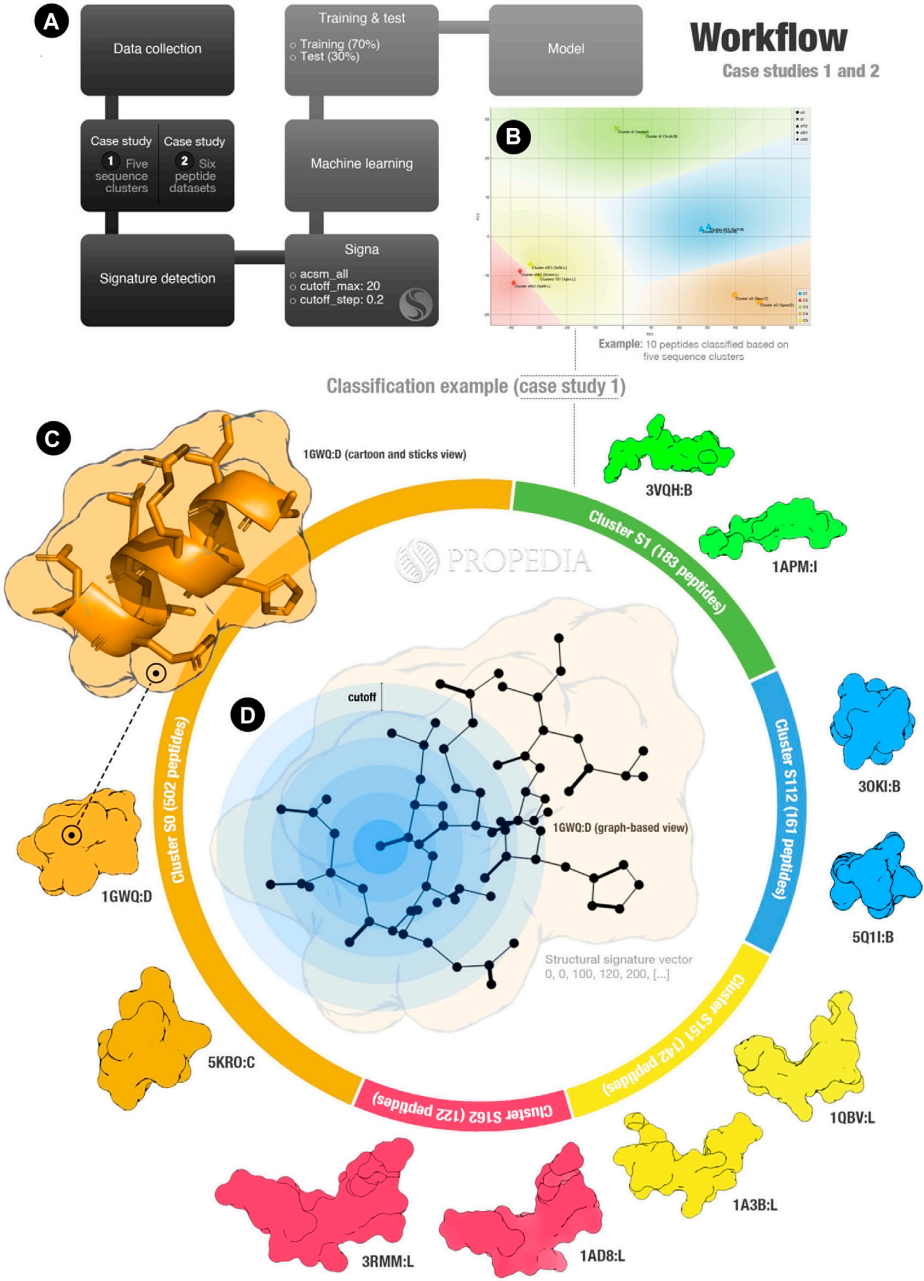
Propedia’s peptide signature validation workflow. **(A)** Workflow overall. **(B)** Example of clusters representation for case study 1 (x- and *y*-axis represent PCA1 and PCA2). Colours represent regions for classifying sequence clusters (generated using Orange Data Mining). **(C)** Infographic represents the case study 1 entrance. **(D)** Graph-based structural signature representation. Cutoffs are represented by blue circles. Figure generated using ChimeraX and Opensource PyMOL.

In this section, we describe two case studies performed to validate the new dataset. The steps used in this analysis are summarized in Figure 1. First, we collected the data from PDB and updated PropediaDB. Then, we collected the data corresponding to each case study, detected the structural signatures, and ran machine learning algorithms to see if the structural signatures were a good method to detect clusters based on two different metrics: sequence similarities (case study 1) and similarities in the peptide role and function (case study 2). We expect that the machine learning methods can classify each group correctly (as the example shown in Figure 1B).

In the first case study, we collected 1,111 peptide structures from the five most populated sequence clusters: S0, S1, S112, S151, and S162. Each one of these clusters contains 503, 183, 161, 142, and 122 peptides, respectively (as represented in Figure 1C). In the second case study, we collected 6,238 peptides from six Propedia sub-datasets: Antimicrobial, Enzyme, Hormone, Membrane, Plant, and Viral. These classifications were obtained from text mining analysis of 3D-structure files collected from Protein Data Bank.

For both case studies, we calculated the structural signatures. A representation of the graph-based structural model can be seen in Figure 1D. The blue rings represent the cutoff used in the aCSM method to detect atoms at determined distances (note that this method is applied for each atom of the peptide).

Then, we imported the signature vectors to the Orange Data Mining tool and performed classification using seven supervising machine learning algorithms: kNN, SVM, Neural Network, Gradient Boosting, Logistic Regression, Decision Tree, and Random Forest. Training and testing were performed using 70% and 30% of the datasets, respectively. The experiments were performed in triplicate (the results are the average of metrics obtained).

Table 1 summarizes the main results of our analyses. For AUC (Area under the ROC Curve), CA (classification accuracy), F1 (Harmonic Precision-Recall Mean), precision, and recall, high values can indicate good prediction results. The “correctly predicted (by class)” column shows the percentual of hits for a determined cluster (higher is better).

**TABLE 1:** Machine learning analysis results. The confusion matrices and ROC plots are available in the Supplementary Material.

Case study 1: classifying the sequence clusters						Correctly predicted (by class)				
Model	AUC	CA	F1	Precision	Recall	S0	S1	S112	S151	S162
kNN	0.984	0.952	0.952	0.952	0.952	97.8%	99.4%	95,1%	89,1%	85,6%
Tree	0.984	0.954	0.954	0.955	0.954	98,7%	94,5%	97,2%	86,0%	91,9%
SVM	0.992	0.937	0.937	0.942	0.937	100%	100%	100%	64,3%	84,7%
Random Forest	0.996	0.968	0.968	0.969	0.968	99,3%	98,2%	100%	86,0%	93,8%
Neural Network	0.992	0.915	0.910	0.919	0.915	100%	100%	100%	85,3%	40,5%
Logistic Regression	0.996	0.974	0.974	0.974	0.974	99,8%	100%	100%	88,4%	91,0%
Gradient Boosting	0.998	0.983	0.983	0.983	0.983	99,8%	100%	100%	93,8%	92,8%

Case study 2: Propedia sub-datasets						Correctly predicted (by class)					
Model	AUC	CA	F1	Precision	Recall	Antimicrobial	Enzyme	Hormone	Membrane	Plant	Viral
kNN	0.872	0.924	0.921	0.920	0.924	55,6%	96,7%	69,8%	51,9%	88,7%	56,1%
Tree	0.772	0.896	0.891	0.887	0.896	0.0%	95,8%	58,3%	27.4%	76,6%	45,8%
SVM	0.594	0.306	0.409	0.780	0.306	33,3%	30,7%	12,5%	29,6%	42,9%	32,2%
Random Forest	0.917	0.926	0.918	0.921	0.926	44,4%	98,5%	62,5%	38,5%	85,3%	42,8%
Neural Network	0.861	0.909	0.898	0.897	0.909	22,2%	97,5%	63,5%	36,3%	79.2%	29,9%
Logistic Regression	0.878	0.909	0.901	0.898	0.909	11,1%	96,9%	66,1%	28,1%	86,1%	37,5%
Gradient Boosting	0.931	0.919	0.908	0.913	0.919	55,6%	98,4%	64,6%	33,3%	81,0%	34,5%

**Notes:**In this table, the first column indicates the machine-learning method used in the prediction for both case studies. The next five columns indicate the metrics used to assess the quality of the models. The closer to 1, the better the model. The "Corrected predicted (by class)" column indicates the percentage values of correct answers within that category for both case studies. The first case study has five categories: S0, S1, S122, S151, and S162. On the other hand, the second case study presents six categories: antimicrobial, enzyme, hormone, membrane, plant, and viral.

As we hypothesized, the structural signatures demonstrated to be a good method for representing peptide structures. The predictions based on sequence similarities obtained an accuracy of over 0.98 using the Gradient Boosting algorithm. Additionally, the most errors occurred for clusters S151 and S162, which have visually similar structures as shown in Figure 1C (structures yellow and red at the bottom). This could indicate that the structural signatures can detect similarities with more details than sequence-based strategies.

Compared to the first case study, the second case study presented lower values for AUC, CA, F1, precision, and recall. Although for many algorithms, accuracy values were superior to 0.9, which is considered a good predictor. For the results extracted by classes, lower values for the percentual of corrected predicted were found in several groups. This can significate that the peptides share few structural similarities, the PDB classification was not good for classification, or even that some peptides can share characteristics of different groups, which could be a good indicator that they could be used in different therapeutic goals. Another possibility is that dataset imbalance impacted the final prediction, including a bias for correctly classifying the cluster with more samples (enzyme). In this case, the KNN algorithm could reduce the bias problem once it considers only the three closer neighbour structures for classification (which we can observe in Table 1).

Lastly, we tried to explain which characteristics are more important to classification in each group. Our results indicate that the presence of aromatic/acceptor atom interactions, as well as disulfide bonds, can be responsible for this. Although more studies would be necessary for more precise conclusions (details were included in Supplementary Material).

## 4 Conclusion

Here, we presented Propedia v2.3, the first major update to the Propedia database, with a 150% increase in the number of complexes, in addition to a new dataset developed using graph-based structural signatures. These improvements enable new use cases and applications such as the ones presented as case studies in this work. As such, we expect the database to become more useful to its current users as well as to new users.

Additionally, we also turn available the Propedia source code into an open-source project at https://github.com/LBS-UFMG/propedia.

## Data Availability

The original contributions presented in the study are included in the article/Supplementary Material, further inquiries can be directed to the corresponding author.
