# Supplementary material for: Propedia v2.3: A novel representation approach for the peptide-protein interaction database using graph-based structural signatures
Source: Front Bioinform. 2023 Feb 16;3:1103103. doi: 10.3389/fbinf.2023.1103103 (PMC9978205; doi:10.3389/fbinf.2023.1103103)
Supplement: Supplementary file 1 [file DataSheet1.PDF]

Supplementary Material

1 Supplementary Data

1.1 Graph-based structural signatures

Graph-based structural signatures are representative vectors of macromolecules. In this approach, proteins or peptides are modelled as graphs, in which atoms are nodes, and an edge connects all the atom pairs in a determined cutoff. A cumulative vector of atom pairs at a determined cutoff represents the macromolecule. Signatures can be obtained using the CSM (Cutoff Scanning Matrix) method and its variations, such as CSM-classic (a method that uses only the alpha-carbon atom), aCSM (a method that uses all atoms), aCSM-HP (separate interactions between polar and hydrophobic atoms), and aCSM-ALL (that uses eight types of atoms: hydrophobic, positive, negative, acceptor, donor, aromatic, sulfur, and neutral).

In Supplementary table 1, we can see an example of aCSM signature in a CSV file. In the first line, we can see the signature header with the distance cutoffs. In the second line, we can see the signature of the peptide in the "chain B" from the PDB structure 5ZLM (available at https://www.rcsb.org/structure/5zml). In this example, we considered a cutoff limit of 20Å with a cutoff step of 0.2Å (this will return 100 numbers).

Supplementary table 1. aCSM classical signature. Fragment of the CSV file of structural signatures from Propedia peptides.

peptide,20.0-19.8,19.8-19.6,19.6-19.4,19.4-19.2,19.2-19.0,19.0-18.8,18.8-18.6,18.6-18.4,18.4-18.2,18.2-18.0,18.0-17.8,17.8-17.6,17.6-17.4,17.4-17.2,17.2-17.0,17.0-16.8,16.8-16.6,16.6-16.4,16.4-16.2,16.2-16.0,16.0-15.8,15.8-15.6,15.6-15.4,15.4-15.2,15.2-15.0,15.0-14.8,14.8-14.6,14.6-14.4,14.4-14.2,14.2-14.0,14.0-13.8,13.8-13.6,13.6-13.4,13.4-13.2,13.2-13.0,13.0-12.8,12.8-12.6,12.6-12.4,12.4-12.2,12.2-12.0,12.0-11.8,11.8-11.6,11.6-11.4,11.4-11.2,11.2-11.0,11.0-10.8,10.8-10.6,10.6-10.4,10.4-10.2,10.2-10.0,10.0-9.8,9.8-9.6,9.6-9.4,9.4-9.2,9.2-9.0,9.0-8.8,8.8-8.6,8.6-8.4,8.4-8.2,8.2-8.0,8.0-7.8,7.8-7.6,7.6-7.4,7.4-7.2,7.2-7.0,7.0-6.8,6.8-6.6,6.6-6.4,6.4-6.2,6.2-6.0,6.0-5.8,5.8-5.6,5.6-5.4,5.4-5.2,5.2-5.0,5.0-4.8,4.8-4.6,4.6-4.4,4.4-4.2,4.2-4.0,4.0-3.8,3.8-3.6,3.6-3.4,3.4-3.2,3.2-3.0,3.0-2.8,2.8-2.6,2.6-2.4,2.4-2.2,2.2-2.0,2.0-1.8,1.8-1.6,1.6-1.4,1.4-1.2,1.2-1.0,1.0-0.8,0.8-0.6,0.6-0.4,0.4-0.2,0.2-0.0
peptide/5zml_B.pdb,5604,5575,5552,5532,5501,5479,5462,5437,5405,5375,5340,5304,5278,5246,5215,5177,5136,5096,5057,5019,4974,4946,4887,4847,4796,4732,4687,4633,4587,4533,4459,4384,4327,4274,4219,4154,4086,4012,3954,3871,3809,3736,3655,3576,3509,3440,3367,3279,3213,3112,3029,2933,2844,2763,2683,2593,2513,2437,2344,2242,2148,2066,1972,1878,1786,1707,1618,1530,1444,1349,1257,1175,1094,1000,923,839,754,671,593,532,505,449,397,360,327,292,263,245,153,109,109,109,109,45,0,0,0,0,0,0
[...] More 27930 lines.

This table indicates that the peptide presented in the chain B of the PDB 5ZML has 5604 atom pairs located at a distance range from 19.8Å to 20Å, 5575 at a distance range from 19.6Å to 19.8Å, 5552 at a distance range from 19.4Å to 19.6Å, and so on. Note that CSM traditional method performs a cutoff countdown starting from the cutoff limit until zero (variating the cutoff step).

5ZML-B is a peptide of sequence “KKRYSRXQLLXFRR” complexed with a protein (chain A). In Supplementary Figure 1, we can see the structure of 5ZLM represents stapled peptides tailored against the initiation of translation. The peptide is represented as sticks with a yellow semi-transparent surface, while the protein is shown as a cartoon with a green surface.

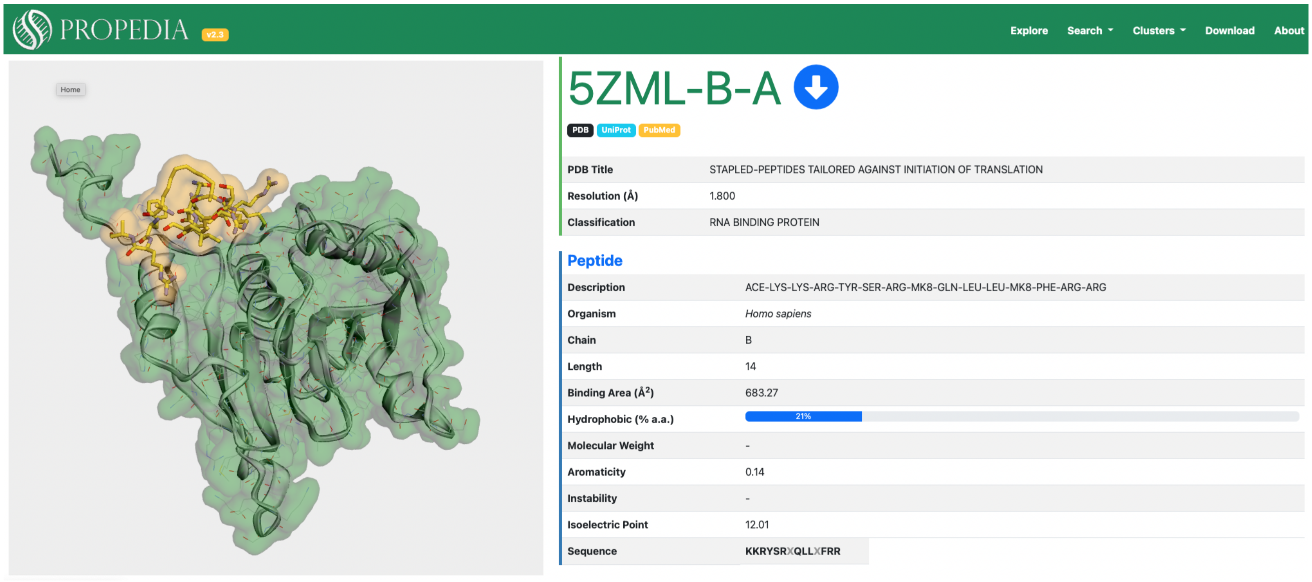

Supplementary figure 1. Structure of 5ZML (peptide B is complexed with chain A). Available at http://bioinfo.dcc.ufmg.br/propedia2/index.php/complex/view/5zml-B-A.

The aCSM-ALL is a variation of the CSM classical algorithm. It considers the pharmacological properties of atoms when calculating the signature. Hence, it returns a higher vector of numbers. For example, a signature determined for a cutoff limit of 20Å with a cutoff step of 0.2Å will have 3600 numbers, as shown in Supplementary Table 2.

Supplementary table 2. aCSM-ALL signature for chain B of 5zml.

peptide/5zml_B.pdb, 76, 156, 310, 344, 0, 385, 0, 0, 66, 309, 336, 0, 371, 0, 0, 285, 649, 0, 733, 0, 0, 348, 0, 801, 0, 0, 0, 0, 0, 435, 0, 0, 0, 0, 0,
76, 156, 309, 340, 0, 384, 0, 0, 66, 309, 336, 0, 370, 0, 0, 282, 646, 0, 728, 0, 0, 345, 0, 797, 0, 0, 0, 0, 0, 431, 0, 0, 0, 0, 0, 76, 156, 308, 340, 0,
382, 0, 0, 66, 309, 335, 0, 369, 0, 0, 281, 641, 0, 723, 0, 0, 343, 0, 792, 0, 0, 0, 0, 0, 431, 0, 0, 0, 0, 0, 76, 156, 307, 339, 0, 381, 0, 0, 66, 309, 335,
0, 369, 0, 0, 280, 639, 0, 719, 0, 0, 340, 0, 789, 0, 0, 0, 0, 0, 427, 0, 0, 0, 0, 0, 75, 156, 306, 338, 0, 377, 0, 0, 66, 308, 335, 0, 369, 0, 0, 277, 636, 0,
714, 0, 0, 336, 0, 783, 0, 0, 0, 0, 0, 425, 0, 0, 0, 0, 0, 75, 156, 303, 337, 0, 373, 0, 0, 66, 308, 334, 0, 369, 0, 0, 275, 632, 0, 711, 0, 0, 335, 0, 781, 0,
0, 0, 0, 0, 424, 0, 0, 0, 0, 0, 74, 156, 302, 336, 0, 373, 0, 0, 66, 307, 333, 0, 369, 0, 0, 275, 629, 0, 707, 0, 0, 334, 0, 778, 0, 0, 0, 0, 0, 423, 0, 0, 0,
0, 0, 73, 156, 301, 333, 0, 371, 0, 0, 66, 307, 333, 0, 368, 0, 0, 272, 627, 0, 704, 0, 0, 329, 0, 776, 0, 0, 0, 0, 0, 421, 0, 0, 0, 0, 0, 73, 156, 300, 328,
0, 370, 0, 0, 66, 307, 332, 0, 368, 0, 0, 270, 621, 0, 699, 0, 0, 326, 0, 770, 0, 0, 0, 0, 0, 419, 0, 0, 0, 0, 0, 73, 156, 295, 324, 0, 369, 0, 0, 66, 306,
332, 0, 367, 0, 0, 270, 618, 0, 694, 0, 0, 324, 0, 767, 0, 0, 0, 0, 0, 414, 0, 0, 0, 0, 0, 73, 156, 293, 321, 0, 368, 0, 0, 66, 306, 332, 0, 367, 0, 0, 269,
610, 0, 686, 0, 0, 321, 0, 764, 0, 0, 0, 0, 0, 408, 0, 0, 0, 0, 0, 73, 156, 292, 320, 0, 364, 0, 0, 66, 306, 330, 0, 367, 0, 0, 263, 604, 0, 683, 0, 0, 318, 0,
756, 0, 0, 0, 0, 0, 406, 0, 0, 0, 0, 0, 72, 156, 290, 319, 0, 364, 0, 0, 66, 305, 329, 0, 366, 0, 0, 261, 601, 0, 681, 0, 0, 313, 0, 751, 0, 0, 0, 0, 0, 404,
0, 0, 0, 0, 0, 72, 156, 289, 319, 0, 361, 0, 0, 66, 304, 328, 0, 366, 0, 0, 257, 595, 0, 675, 0, 0, 309, 0, 746, 0, 0, 0, 0, 0, 403, 0, 0, 0, 0, 0, 72, 156,
287, 317, 0, 359, 0, 0, 66, 304, 327, 0, 366, 0, 0, 257, 590, 0, 670, 0, 0, 305, 0, 738, 0, 0, 0, 0, 0, 401, 0, 0, 0, 0, 0, 71, 156, 287, 314, 0, 356, 0, 0,
66, 299, 327, 0, 365, 0, 0, 254, 588, 0, 665, 0, 0, 303, 0, 728, 0, 0, 0, 0, 0, 398, 0, 0, 0, 0, 0, 70, 156, 284, 311, 0, 353, 0, 0, 66, 296, 326, 0, 365, 0,
0, 251, 582, 0, 658, 0, 0, 299, 0, 723, 0, 0, 0, 0, 0, 396, 0, 0, 0, 0, 0, 70, 156, 281, 308, 0, 350, 0, 0, 66, 295, 323, 0, 364, 0, 0, 248, 579, 0, 650, 0, 0,
296, 0, 717, 0, 0, 0, 0, 0, 393, 0, 0, 0, 0, 0, 69, 156, 277, 307, 0, 349, 0, 0, 66, 293, 322, 0, 362, 0, 0, 244, 574, 0, 642, 0, 0, 294, 0, 712, 0, 0, 0, 0, 0,
0, 390, 0, 0, 0, 0, 0, 69, 156, 276, 305, 0, 347, 0, 0, 66, 289, 322, 0, 361, 0, 0, 239, 567, 0, 635, 0, 0, 292, 0, 706, 0, 0, 0, 0, 0, 389, 0, 0, 0, 0, 0, 67,

156, 274, 302, 0, 340, 0, 0, 66, 287, 322, 0, 360, 0, 0, 237, 563, 0, 630, 0, 0, 289, 0, 699, 0, 0, 0, 0, 0, 382, 0, 0, 0, 0, 0, 67, 156, 272, 300, 0, 339, 0, 0, 66, 283, 320, 0, 358, 0, 0, 234, 562, 0, 627, 0, 0, 288, 0, 694, 0, 0, 0, 0, 0, 380, 0, 0, 0, 0, 0, 66, 156, 265, 295, 0, 334, 0, 0, 66, 280, 320, 0, 357, 0, 0, 233, 554, 0, 620, 0, 0, 282, 0, 687, 0, 0, 0, 0, 0, 372, 0, 0, 0, 0, 0, 65, 156, 262, 294, 0, 331, 0, 0, 66, 274, 319, 0, 355, 0, 0, 231, 552, 0, 618, 0, 0, 279, 0, 681, 0, 0, 0, 0, 0, 364, 0, 0, 0, 0, 0, 64, 156, 262, 293, 0, 328, 0, 0, 66, 269, 316, 0, 351, 0, 0, 225, 547, 0, 607, 0, 0, 277, 0, 674, 0, 0, 0, 0, 0, 0, 361, 0, 0, 0, 0, 0, 62, 156, 255, 290, 0, 322, 0, 0, 66, 262, 314, 0, 347, 0, 0, 223, 537, 0, 599, 0, 0, 275, 0, 666, 0, 0, 0, 0, 0, 358, 0, 0, 0, 0, 0, 62, 156, 253, 288, 0, 318, 0, 0, 66, 256, 313, 0, 343, 0, 0, 221, 531, 0, 594, 0, 0, 272, 0, 662, 0, 0, 0, 0, 0, 352, 0, 0, 0, 0, 0, 62, 154, 249, 281, 0, 313, 0, 0, 66, 252, 311, 0, 340, 0, 0, 219, 526, 0, 587, 0, 0, 270, 0, 653, 0, 0, 0, 0, 0, 350, 0, 0, 0, 0, 0, 62, 153, 248, 279, 0, 311, 0, 0, 66, 244, 309, 0, 334, 0, 0, 218, 519, 0, 581, 0, 0, 268, 0, 647, 0, 0, 0, 0, 0, 348, 0, 0, 0, 0, 0, 62, 153, 245, 278, 0, 306, 0, 0, 66, 238, 305, 0, 331, 0, 0, 217, 513, 0, 571, 0, 0, 265, 0, 640, 0, 0, 0, 0, 0, 343, 0, 0, 0, 0, 0, 61, 150, 237, 271, 0, 302, 0, 0, 66, 236, 300, 0, 327, 0, 0, 215, 504, 0, 566, 0, 0, 260, 0, 628, 0, 0, 0, 0, 0, 336, 0, 0, 0, 0, 0, 60, 147, 233, 266, 0, 301, 0, 0, 66, 229, 295, 0, 323, 0, 0, 212, 494, 0, 556, 0, 0, 259, 0, 613, 0, 0, 0, 0, 0, 330, 0, 0, 0, 0, 0, 60, 146, 227, 263, 0, 292, 0, 0, 66, 223, 291, 0, 320, 0, 0, 208, 490, 0, 547, 0, 0, 259, 0, 606, 0, 0, 0, 0, 0, 329, 0, 0, 0, 0, 0, 58, 145, 223, 261, 0, 290, 0, 0, 66, 218, 288, 0, 319, 0, 0, 204, 480, 0, 540, 0, 0, 257, 0, 602, 0, 0, 0, 0, 0, 323, 0, 0, 0, 0, 0, 58, 142, 221, 257, 0, 287, 0, 0, 66, 215, 281, 0, 315, 0, 0, 202, 475, 0, 529, 0, 0, 254, 0, 598, 0, 0, 0, 0, 0, 319, 0, 0, 0, 0, 0, 57, 140, 220, 251, 0, 283, 0, 0, 66, 214, 275, 0, 311, 0, 0, 195, 467, 0, 519, 0, 0, 252, 0, 591, 0, 0, 0, 0, 0, 313, 0, 0, 0, 0, 0, 55, 140, 219, 248, 0, 281, 0, 0, 66, 203, 269, 0, 309, 0, 0, 189, 460, 0, 508, 0, 0, 250, 0, 579, 0, 0, 0, 0, 0, 310, 0, 0, 0, 0, 0, 54, 138, 215, 244, 0, 276, 0, 0, 66, 202, 261, 0, 305, 0, 0, 184, 451, 0, 503, 0, 0, 245, 0, 566, 0, 0, 0, 0, 0, 302, 0, 0, 0, 0, 0, 53, 137, 211, 239, 0, 273, 0, 0, 66, 201, 257, 0, 303, 0, 0, 182, 441, 0, 497, 0, 0, 242, 0, 556, 0, 0, 0, 0, 0, 296, 0, 0, 0, 0, 0, 52, 137, 207, 235, 0, 268, 0, 0, 66, 195, 247, 0, 296, 0, 0, 178, 431, 0, 486, 0, 0, 237, 0, 546, 0, 0, 0, 0, 0, 290, 0, 0, 0, 0, 0, 51, 135, 204, 232, 0, 265, 0, 0, 66, 193, 239, 0, 294, 0, 0, 173, 424, 0, 474, 0, 0, 234, 0, 537, 0, 0, 0, 0, 0, 288, 0, 0, 0, 0, 0, 50, 134, 199, 226, 0, 261, 0, 0, 66, 190, 231, 0, 289, 0, 0, 172, 414, 0, 467, 0, 0, 230, 0, 527, 0, 0, 0, 0, 0, 280, 0, 0, 0, 0, 0, 50, 133, 193, 224, 0, 259, 0, 0, 66, 188, 217, 0, 289, 0, 0, 164, 401, 0, 453, 0, 0, 226, 0, 515, 0, 0, 0, 0, 0, 277, 0, 0, 0, 0, 0, 48, 131, 192, 218, 0, 255, 0, 0, 66, 184, 210, 0, 284, 0, 0, 163, 389, 0, 442, 0, 0, 219, 0, 502, 0, 0, 0, 0, 0, 0, 273, 0, 0, 0, 0, 0, 48, 131, 191, 213, 0, 248, 0, 0, 66, 180, 200, 0, 281, 0, 0, 158, 381, 0, 432, 0, 0, 213, 0, 498, 0, 0, 0, 0, 0, 269, 0, 0, 0, 0, 0, 47, 129, 189, 212, 0, 242, 0, 0, 66, 177, 195, 0, 277, 0, 0, 151, 374, 0, 423, 0, 0, 209, 0, 487, 0, 0, 0, 0, 0, 262, 0, 0, 0, 0, 0, 46, 127, 182, 210, 0, 238, 0, 0, 66, 173, 191, 0, 270, 0, 0, 146, 364, 0, 416, 0, 0, 203, 0, 479, 0, 0, 0, 0, 0, 256, 0, 0, 0, 0, 0, 46, 126, 176, 206, 0, 232, 0, 0, 66, 168, 183, 0, 265, 0, 0, 141, 350, 0, 405, 0, 0, 200, 0, 467, 0, 0, 0, 0, 0, 248, 0, 0, 0, 0, 0, 45, 122, 174, 199, 0, 229, 0, 0, 66, 163, 178, 0, 258, 0, 0, 140, 346, 0, 393, 0, 0, 198, 0, 457, 0, 0, 0, 0, 0, 245, 0, 0, 0, 0, 0, 45, 120, 169, 195, 0, 223, 0, 0, 66, 158, 170, 0, 247, 0, 0, 131, 330, 0, 381, 0, 0, 190, 0, 447, 0, 0, 0, 0, 0, 240, 0, 0, 0, 0, 0, 45, 116, 165, 186, 0, 218, 0, 0, 66, 155, 163, 0, 234, 0, 0, 127, 321, 0, 374, 0, 0, 183, 0, 443, 0, 0, 0, 0, 0, 233, 0, 0, 0, 0, 0, 44, 110, 161, 184, 0, 213, 0, 0, 66, 148, 159, 0, 224, 0, 0, 119, 311, 0, 364, 0, 0, 176, 0, 428, 0, 0, 0, 0, 0, 226, 0, 0, 0, 0, 0, 42, 108, 153, 181, 0, 210, 0, 0, 66, 141, 151, 0, 219, 0, 0, 113, 304, 0, 352, 0, 0, 169, 0, 417, 0, 0, 0, 0, 0, 218, 0, 0, 0, 0, 0, 41, 106, 148, 175, 0, 205, 0, 0, 66, 138, 144, 0, 208, 0, 0, 110, 295, 0, 344, 0, 0, 167, 0, 403, 0, 0, 0, 0, 0, 213, 0, 0, 0, 0, 0, 40, 102, 143, 173, 0, 199, 0, 0, 66, 134, 139, 0, 196, 0, 0, 105, 286, 0, 336, 0, 0, 165, 0, 390, 0, 0, 0, 0, 0, 209, 0, 0, 0, 0, 0, 38, 100, 136, 165, 0, 193, 0, 0, 66, 122, 134, 0, 190, 0, 0, 102, 280, 0, 325, 0, 0, 162, 0, 378, 0, 0, 0, 0, 0, 202, 0, 0, 0, 0, 0, 35, 94, 132, 162, 0, 190, 0, 0, 66, 118, 134, 0, 179, 0, 0, 101, 269, 0, 310, 0, 0, 157, 0, 369, 0, 0, 0, 0, 0, 197, 0, 0, 0, 0, 0, 35, 90, 129, 156, 0, 182, 0, 0, 66, 117, 132, 0, 173, 0, 0, 97, 260, 0, 301, 0, 0, 152, 0, 357, 0, 0, 0, 0, 0, 190, 0, 0, 0, 0, 0, 34, 85, 124, 151, 0, 179, 0, 0, 64, 116, 125, 0, 165, 0, 0, 91, 248, 0, 281, 0, 0, 146, 0, 352, 0, 0, 0, 0, 0, 183, 0, 0, 0, 0, 0, 32, 80, 115, 147, 0, 175, 0, 0, 63, 112, 120, 0, 156, 0, 0, 85, 237, 0, 262, 0, 0, 140, 0, 341, 0, 0, 0, 0, 0, 177, 0, 0, 0, 0, 0, 32, 76, 112, 139, 0, 172, 0, 0, 62, 101, 112, 0, 144, 0, 0, 83, 233, 0, 253, 0, 0, 131, 0, 326, 0, 0, 0, 0, 0, 172, 0, 0, 0, 0, 0, 32, 69, 105, 133, 0, 167, 0, 0, 61, 96, 109, 0, 138, 0, 0, 79, 223, 0, 246, 0, 0, 125, 0, 316, 0, 0, 0, 0, 0, 167, 0, 0, 0, 0, 0, 31, 63, 102, 126, 0, 162, 0, 0, 60, 92, 106, 0, 127, 0, 0, 72, 215, 0, 236, 0, 0, 118, 0, 305, 0, 0, 0, 0, 0, 157, 0, 0, 0, 0, 0, 30, 59, 95, 122, 0, 154, 0, 0, 57, 86, 98, 0, 123, 0, 0, 69, 208, 0, 223, 0, 0, 110, 0, 293, 0, 0, 0, 0, 0, 151, 0, 0, 0, 0, 0, 30, 56, 91, 119, 0, 150, 0, 0, 56, 83, 90, 0, 114, 0, 0, 66, 193, 0, 204, 0, 0, 103, 0, 282, 0, 0, 0, 0, 0, 149, 0, 0, 0, 0, 0, 30, 49, 89, 117, 0, 143, 0, 0, 55, 77, 87, 0, 109, 0, 0, 61, 181, 0, 197, 0, 0, 96, 0, 270, 0, 0, 0, 0, 0, 146, 0, 0, 0, 0, 0, 29, 47, 85, 113, 0, 137, 0, 0, 53, 69, 76, 0, 101, 0, 0, 59, 174, 0, 192, 0, 0, 93, 0, 248, 0, 0, 0, 0, 0, 142, 0, 0, 0, 0, 0, 27, 45, 80, 110, 0, 129, 0, 0, 51, 63, 70, 0, 96, 0, 0, 55, 166, 0, 184, 0, 0, 84, 0, 234, 0, 0, 0, 0, 0, 136, 0, 0, 0, 0, 0, 25, 39, 77, 103, 0, 123, 0, 0, 48, 56, 67, 0, 88, 0, 0, 51, 163, 0, 175, 0, 0, 76, 0, 222, 0, 0, 0, 0, 0, 131, 0, 0, 0, 0, 0, 22, 33, 73, 97, 0, 120, 0, 0, 47, 50, 60, 0, 81, 0, 0, 46, 156, 0, 168, 0, 0, 67, 0, 211, 0, 0, 0, 0, 0, 118, 0, 0, 0, 0, 0, 21, 32, 66, 91, 0, 116, 0, 0, 44, 49, 49, 0, 71, 0, 0, 40, 144, 0, 162, 0, 0, 61, 0, 197, 0, 0, 0, 0, 0, 114, 0, 0, 0, 0, 0, 20, 30, 59, 86, 0, 113, 0, 0, 40, 45, 43, 0, 59, 0, 0, 38, 137, 0, 157, 0, 0, 54, 0, 186, 0, 0, 0, 0, 0, 108, 0, 0, 0, 0, 0, 20, 27, 56, 80, 0, 107, 0, 0, 40, 40, 40, 0, 58, 0, 0, 38, 121, 0, 142, 0, 0, 48, 0, 173, 0, 0, 0, 0, 0, 104, 0, 0, 0, 0, 0, 19, 25, 55, 76, 0, 103, 0, 0, 39, 32, 35, 0, 47, 0, 0, 37, 105, 0, 129, 0, 0, 43, 0, 164, 0, 0, 0, 0, 0, 91, 0, 0, 0, 0, 0, 19, 24, 50, 66, 0, 97, 0, 0, 36, 31, 31, 0, 41, 0, 0, 34, 99, 0, 120, 0, 0, 38, 0, 153, 0, 0, 0, 0, 0, 84, 0, 0, 0, 0, 0, 17, 20, 47, 62, 0, 90, 0, 0, 36, 25, 25, 0, 36, 0, 0, 30, 91, 0, 111, 0, 0, 34, 0, 139, 0, 0, 0, 0, 0, 76, 0, 0, 0, 0, 0, 12, 15, 43, 55, 0, 85, 0, 0, 34, 19, 20, 0, 30, 0, 0, 27, 82, 0, 107, 0, 0, 34, 0, 120, 0, 0, 0, 0, 0, 71, 0, 0, 0, 0, 0, 8, 13, 43, 49, 0, 78, 0, 0, 33, 16, 18, 0, 22, 0, 0, 26, 73, 0, 95, 0, 0, 32, 0, 100, 0, 0, 0, 0, 0, 65, 0, 0, 0, 0, 0, 8, 9, 42, 42, 0, 75, 0, 0, 32, 13, 16, 0, 16, 0, 0, 23, 67, 0, 87, 0, 0, 32, 0, 82, 0, 0, 0, 0, 0, 49, 0, 0, 0, 0, 0, 7, 8, 40, 32, 0, 72, 0, 0, 32, 13, 12, 0, 12, 0, 0, 21, 59, 0, 73, 0, 0, 31, 0, 75, 0, 0, 0, 0, 0, 45, 0, 0, 0, 0, 0, 7, 4, 39, 29, 0, 66, 0, 0, 31, 11, 11, 0, 11, 0, 0, 21, 57, 0, 72, 0, 0, 30, 0, 72, 0, 0, 0, 0, 0, 44, 0, 0, 0, 0, 0, 7, 3, 37, 22, 0, 52, 0, 0, 30, 9, 10, 0, 8, 0, 0, 21, 54, 0, 67, 0, 0, 30, 0, 59, 0, 0, 0, 0, 0, 40, 0, 0, 0, 0, 0, 5, 2, 35, 19, 0, 46, 0, 0, 30, 9, 6, 0, 6, 0, 0, 20, 44, 0, 64, 0, 0, 29, 0, 49, 0, 0, 0, 0, 0, 33, 0, 0, 0, 0, 0, 5, 1, 27, 15, 0, 44, 0, 0, 30, 6, 6, 0, 4, 0, 0, 16, 40, 0, 63, 0, 0, 28, 0, 46, 0, 0, 0, 0, 0, 0, 29, 0, 0, 0, 0, 0, 0, 0, 24, 8, 0, 40, 0, 0, 30, 5, 6, 0, 3, 0, 0, 15, 34, 0, 59, 0, 0, 28, 0, 46, 0, 0, 0, 0, 0, 0, 29, 0, 0, 0, 0, 0, 0, 0, 18, 3, 0, 38, 0, 0, 30, 4, 6, 0, 3, 0, 0, 14, 25, 0, 58, 0, 0, 28, 0, 40, 0, 0, 0, 0, 0, 0, 25, 0, 0, 0, 0, 0, 0, 0, 12, 1, 0, 31, 0, 0, 30, 3, 6, 0, 2, 0, 0, 11, 20, 0, 57, 0, 0, 28, 0, 39, 0, 0, 0, 0, 0, 0, 23, 0, 0, 0, 0, 0, 0, 0, 10, 1, 0, 25, 0, 0, 24, 3, 6, 0, 2, 0, 0, 9, 20, 0, 57, 0, 0, 28, 0, 37, 0, 0, 0, 0, 0, 0, 23, 0, 0, 0, 0, 0, 0, 0, 10, 0, 0, 20, 0, 0, 20, 3, 2, 0, 0, 0, 0, 9, 5, 0, 38, 0, 0, 17, 0, 14, 0, 0, 0, 0, 0, 0, 15, 0, 0, 0, 0, 0, 0, 0, 0, 0, 0, 13, 0, 0, 12, 1, 2, 0, 0, 0, 0, 0, 3, 0, 34, 0, 0, 17, 0, 14, 0, 0, 0, 0, 0, 0, 13, 0, 0, 0, 0, 0, 0, 0, 0, 0, 0, 13, 0, 0, 12, 1, 2, 0, 0, 0, 0, 0, 3, 0, 34, 0, 0, 17, 0, 14, 0, 0, 0, 0, 0, 0, 13, 0, 0, 0, 0, 0, 0, 0, 0, 0, 0, 13, 0, 0, 12, 1, 2, 0, 0, 0, 0, 0, 3, 0, 34, 0, 0, 17, 0, 14, 0, 0, 0, 0, 0, 0, 13, 0, 0, 0, 0, 0, 0, 0, 0, 0, 0, 0, 0, 0, 0, 0, 0, 0, 0, 0, 0, 13, 0, 0, 12, 1, 2, 0, 0, 0, 0, 0, 3, 0, 34, 0, 0, 17, 0, 14, 0, 0, 0, 0, 0, 0, 13, 0, 0, 0, 0, 0, 0, 0, 0, 0, 0, 13, 0, 0, 12, 1, 0, 0, 0, 0, 0, 0, 0, 0, 19, 0

Note that the aCSM-ALL considers combinations of eight types of contacts (removing redundancy). Thus, the signature vector will have a higher set of numbers. Supplementary Table 3 presents an explanation for each number.

Supplementary table 3. aCSM-ALL header (all eight types of combinations minus redundant ones). Cutoff limit 20Å. Cutoff step: 0.2Å.

peptide,acceptor x acceptor (20.0-19.8),acceptor x donor (20.0-19.8),acceptor x aromatic (20.0-19.8),acceptor x hydrophobic (20.0-19.8),acceptor x negative (20.0-19.8),acceptor x neutral (20.0-19.8),acceptor x positive (20.0-19.8),acceptor x sulfide (20.0-19.8),donor x donor (20.0-19.8),donor x aromatic (20.0-19.8),donor x hydrophobic (20.0-19.8),donor x negative (20.0-19.8),donor x neutral (20.0-19.8),donor x positive (20.0-19.8),donor x sulfide (20.0-19.8),aromatic x aromatic (20.0-19.8),aromatic x hydrophobic (20.0-19.8),aromatic x negative (20.0-19.8),aromatic x neutral (20.0-19.8),aromatic x positive (20.0-19.8),aromatic x sulfide (20.0-19.8),hydrophobic x hydrophobic (20.0-19.8),hydrophobic x negative (20.0-19.8),hydrophobic x neutral (20.0-19.8),hydrophobic x positive (20.0-19.8),hydrophobic x sulfide (20.0-19.8),negative x negative (20.0-19.8),negative x neutral (20.0-19.8),negative x positive (20.0-19.8),negative x sulfide (20.0-19.8),neutral x neutral (20.0-19.8),neutral x positive (20.0-19.8),neutral x sulfide (20.0-19.8),positive x positive (20.0-19.8),positive x sulfide (20.0-19.8),sulfide x sulfide (20.0-19.8),acceptor x acceptor (19.8-19.6),acceptor x donor (19.8-19.6),acceptor x aromatic (19.8-19.6),acceptor x hydrophobic (19.8-19.6),acceptor x negative (19.8-19.6),acceptor x neutral (19.8-19.6),acceptor x positive (19.8-19.6),acceptor x sulfide (19.8-19.6),donor x donor (19.8-19.6),donor x aromatic (19.8-19.6),donor x hydrophobic (19.8-19.6),donor x negative (19.8-19.6),donor x neutral (19.8-19.6),donor x positive (19.8-19.6),donor x sulfide (19.8-19.6),aromatic x aromatic (19.8-19.6),aromatic x hydrophobic (19.8-19.6),aromatic x negative (19.8-19.6),aromatic x neutral (19.8-19.6),aromatic x positive (19.8-19.6),aromatic x sulfide (19.8-19.6),hydrophobic x hydrophobic (19.8-19.6),hydrophobic x negative (19.8-19.6),hydrophobic x neutral (19.8-19.6),hydrophobic x positive (19.8-19.6),hydrophobic x sulfide (19.8-19.6),negative x negative (19.8-19.6),negative x neutral (19.8-19.6),negative x positive (19.8-19.6),negative x sulfide (19.8-19.6),neutral x neutral (19.8-19.6),neutral x positive (19.8-19.6),neutral x sulfide (19.8-19.6),positive x positive (19.8-19.6),positive x sulfide (19.8-19.6),sulfide x sulfide (19.8-19.6),acceptor x acceptor (19.6-19.4),acceptor x donor (19.6-19.4),acceptor x aromatic (19.6-19.4),acceptor x hydrophobic (19.6-19.4),acceptor x negative (19.6-19.4),acceptor x neutral (19.6-19.4),acceptor x positive (19.6-19.4),acceptor x sulfide (19.6-19.4),donor x donor (19.6-19.4),donor x aromatic (19.6-19.4),donor x hydrophobic (19.6-19.4),donor x negative (19.6-19.4),donor x neutral (19.6-19.4),donor x positive (19.6-19.4),donor x sulfide (19.6-19.4),aromatic x aromatic (19.6-19.4),aromatic x hydrophobic (19.6-19.4),aromatic x negative (19.6-19.4),aromatic x neutral (19.6-19.4),aromatic x positive (19.6-19.4),aromatic x sulfide (19.6-19.4),hydrophobic x hydrophobic (19.6-19.4),hydrophobic x negative (19.6-19.4),hydrophobic x neutral (19.6-19.4),hydrophobic x positive (19.6-19.4),hydrophobic x sulfide (19.6-19.4),negative x negative (19.6-19.4),negative x neutral (19.6-19.4),negative x positive (19.6-19.4),negative x sulfide (19.6-19.4),neutral x neutral (19.6-19.4),neutral x positive (19.6-19.4),neutral x sulfide (19.6-19.4),positive x positive (19.6-19.4),positive x sulfide (19.6-19.4),sulfide x sulfide (19.6-19.4),acceptor x acceptor (19.4-19.2),acceptor x donor (19.4-19.2),acceptor x aromatic (19.4-19.2),acceptor x hydrophobic (19.4-19.2),acceptor x negative (19.4-19.2),acceptor x neutral (19.4-19.2),acceptor x positive (19.4-19.2),acceptor x sulfide (19.4-19.2),donor x donor (19.4-19.2),donor x aromatic (19.4-19.2),donor x hydrophobic (19.4-19.2),donor x negative (19.4-19.2),donor x neutral (19.4-19.2),donor x positive (19.4-19.2),donor x sulfide (19.4-19.2),aromatic x aromatic (19.4-19.2),aromatic x hydrophobic (19.4-19.2),aromatic x negative (19.4-19.2),aromatic x neutral (19.4-19.2),aromatic x positive (19.4-19.2),aromatic x sulfide (19.4-19.2),hydrophobic x hydrophobic (19.4-19.2),hydrophobic x negative (19.4-19.2),hydrophobic x neutral (19.4-19.2),hydrophobic x positive (19.4-19.2),hydrophobic x sulfide (19.4-19.2),negative x negative (19.4-19.2),negative x neutral (19.4-19.2),negative x positive (19.4-19.2),negative x sulfide (19.4-19.2),neutral x neutral (19.4-19.2),neutral x positive (19.4-19.2),neutral x sulfide (19.4-19.2),positive x positive (19.4-19.2),positive x sulfide (19.4-19.2),sulfide x sulfide (19.4-19.2),acceptor x acceptor (19.2-19.0),acceptor x donor (19.2-19.0),acceptor x aromatic (19.2-19.0),acceptor x hydrophobic (19.2-19.0),acceptor x negative (19.2-19.0),acceptor x neutral (19.2-19.0),acceptor x positive (19.2-19.0),acceptor x sulfide (19.2-19.0),donor x donor (19.2-19.0),donor x aromatic (19.2-19.0),donor x hydrophobic (19.2-19.0),donor x negative (19.2-19.0),donor x neutral (19.2-19.0),donor x positive (19.2-19.0),donor x sulfide (19.2-19.0),aromatic x aromatic (19.2-19.0),aromatic x hydrophobic (19.2-19.0),aromatic x negative (19.2-19.0),aromatic x neutral (19.2-19.0),aromatic x positive (19.2-19.0),aromatic x sulfide (19.2-19.0),hydrophobic x hydrophobic (19.2-19.0),hydrophobic x negative (19.2-19.0),hydrophobic x neutral (19.2-19.0),hydrophobic x positive (19.2-19.0),hydrophobic x sulfide (19.2-19.0),negative x negative (19.2-19.0),negative x neutral (19.2-19.0),negative x positive (19.2-19.0),negative x sulfide (19.2-19.0),neutral x neutral (19.2-19.0),neutral x positive (19.2-19.0),neutral x sulfide (19.2-19.0),positive x positive (19.2-19.0),positive x sulfide (19.2-19.0),sulfide x sulfide (19.2-19.0),acceptor x acceptor (19.0-18.8),acceptor x donor (19.0-18.8),acceptor x aromatic (19.0-18.8),acceptor x hydrophobic (19.0-18.8),acceptor x negative (19.0-18.8),acceptor x neutral (19.0-18.8),acceptor x positive (19.0-18.8),acceptor x sulfide (19.0-18.8),donor x donor (19.0-18.8),donor x aromatic (19.0-18.8),donor x hydrophobic (19.0-18.8),donor x negative (19.0-18.8),donor x neutral (19.0-18.8),donor x positive (19.0-18.8),donor x sulfide (19.0-18.8),aromatic x aromatic (19.0-18.8),aromatic x hydrophobic (19.0-18.8),aromatic x negative (19.0-18.8),aromatic x neutral (19.0-18.8),aromatic x positive (19.0-18.8),aromatic x sulfide (19.0-18.8),hydrophobic x hydrophobic (19.0-18.8),hydrophobic x negative (19.0-18.8),hydrophobic x neutral (19.0-18.8),hydrophobic x positive (19.0-18.8),hydrophobic x sulfide (19.0-18.8),negative x negative (19.0-18.8),negative x neutral (19.0-18.8),negative x positive (19.0-18.8),negative x sulfide (19.0-18.8),neutral x neutral (19.0-18.8),neutral x positive (19.0-18.8),neutral x sulfide (19.0-18.8),positive x positive (19.0-18.8),positive x sulfide (19.0-18.8),sulfide x sulfide (19.0-18.8),acceptor x acceptor (18.8-18.6),acceptor x donor (18.8-18.6),acceptor x aromatic (18.8-18.6),acceptor x hydrophobic (18.8-18.6),acceptor x negative (18.8-18.6),acceptor x neutral (18.8-18.6),acceptor x positive (18.8-18.6),acceptor x sulfide (18.8-18.6),donor x donor (18.8-18.6),donor x aromatic (18.8-18.6),donor x hydrophobic (18.8-18.6),donor x negative (18.8-18.6),donor x neutral (18.8-18.6),donor x positive (18.8-18.6),donor x sulfide (18.8-18.6),aromatic x aromatic (18.8-18.6),aromatic x hydrophobic (18.8-18.6),aromatic x negative (18.8-18.6),aromatic x neutral (18.8-18.6),aromatic x positive (18.8-18.6),aromatic x sulfide (18.8-18.6),hydrophobic x hydrophobic (18.8-18.6),hydrophobic x negative (18.8-18.6),hydrophobic x neutral (18.8-18.6),hydrophobic x positive (18.8-18.6),hydrophobic x sulfide (18.8-18.6),negative x negative (18.8-18.6),negative x neutral (18.8-18.6),negative x positive (18.8-18.6),negative x sulfide (18.8-18.6),neutral x neutral (18.8-18.6),neutral x positive (18.8-18.6),neutral x sulfide (18.8-18.6),positive x positive (18.8-18.6),positive x sulfide (18.8-18.6),sulfide x sulfide (18.8-18.6),acceptor x acceptor (18.6-18.4),acceptor x donor (18.6-18.4),acceptor x aromatic (18.6-18.4),acceptor x hydrophobic (18.6-18.4),acceptor x negative (18.6-18.4),acceptor x neutral (18.6-18.4),acceptor x positive (18.6-18.4),acceptor x sulfide (18.6-18.4),donor x donor (18.6-18.4),donor x aromatic (18.6-18.4),donor x hydrophobic (18.6-18.4),donor x negative (18.6-18.4),donor x neutral (18.6-18.4),donor x positive (18.6-18.4),donor x sulfide (18.6-18.4),aromatic x aromatic (18.6-18.4),aromatic x hydrophobic (18.6-18.4),aromatic x negative (18.6-18.4),aromatic x neutral (18.6-18.4),aromatic x positive (18.6-18.4),aromatic x sulfide (18.6-18.4),hydrophobic x hydrophobic (18.6-18.4),hydrophobic x negative (18.6-18.4),hydrophobic x neutral (18.6-18.4),hydrophobic x positive (18.6-18.4),hydrophobic x sulfide (18.6-18.4),negative x negative

(18.6-18.4),negative x neutral (18.6-18.4),negative x positive (18.6-18.4),negative x sulfide (18.6-18.4),neutral x neutral (18.6-18.4),neutral x positive (18.6-18.4),neutral x sulfide (18.6-18.4),positive x positive (18.6-18.4),positive x sulfide (18.6-18.4),sulfide x sulfide (18.6-18.4),acceptor x acceptor (18.4-18.2),acceptor x donor (18.4-18.2),acceptor x aromatic (18.4-18.2),acceptor x hydrophobic (18.4-18.2),acceptor x negative (18.4-18.2),acceptor x neutral (18.4-18.2),acceptor x positive (18.4-18.2),acceptor x sulfide (18.4-18.2),donor x donor (18.4-18.2),donor x aromatic (18.4-18.2),donor x hydrophobic (18.4-18.2),donor x negative (18.4-18.2),donor x neutral (18.4-18.2),donor x positive (18.4-18.2),donor x sulfide (18.4-18.2),aromatic x aromatic (18.4-18.2),aromatic x hydrophobic (18.4-18.2),aromatic x negative (18.4-18.2),aromatic x neutral (18.4-18.2),aromatic x positive (18.4-18.2),aromatic x sulfide (18.4-18.2),hydrophobic x hydrophobic (18.4-18.2),hydrophobic x negative (18.4-18.2),hydrophobic x neutral (18.4-18.2),hydrophobic x positive (18.4-18.2),hydrophobic x sulfide (18.4-18.2),negative x negative (18.4-18.2),negative x neutral (18.4-18.2),negative x positive (18.4-18.2),negative x sulfide (18.4-18.2),neutral x neutral (18.4-18.2),neutral x positive (18.4-18.2),neutral x sulfide (18.4-18.2),positive x positive (18.4-18.2),positive x sulfide (18.4-18.2),sulfide x sulfide (18.4-18.2),acceptor x acceptor (18.2-18.0),acceptor x donor (18.2-18.0),acceptor x aromatic (18.2-18.0),acceptor x hydrophobic (18.2-18.0),acceptor x negative (18.2-18.0),acceptor x neutral (18.2-18.0),acceptor x positive (18.2-18.0),acceptor x sulfide (18.2-18.0),donor x donor (18.2-18.0),donor x aromatic (18.2-18.0),donor x hydrophobic (18.2-18.0),donor x negative (18.2-18.0),donor x neutral (18.2-18.0),donor x positive (18.2-18.0),donor x sulfide (18.2-18.0),aromatic x aromatic (18.2-18.0),aromatic x hydrophobic (18.2-18.0),aromatic x negative (18.2-18.0),aromatic x neutral (18.2-18.0),aromatic x positive (18.2-18.0),aromatic x sulfide (18.2-18.0),hydrophobic x hydrophobic (18.2-18.0),hydrophobic x negative (18.2-18.0),hydrophobic x neutral (18.2-18.0),hydrophobic x positive (18.2-18.0),hydrophobic x sulfide (18.2-18.0),negative x negative (18.2-18.0),negative x neutral (18.2-18.0),negative x positive (18.2-18.0),negative x sulfide (18.2-18.0),neutral x neutral (18.2-18.0),neutral x positive (18.2-18.0),neutral x sulfide (18.2-18.0),positive x positive (18.2-18.0),positive x sulfide (18.2-18.0),sulfide x sulfide (18.2-18.0),acceptor x acceptor (18.0-17.8),acceptor x donor (18.0-17.8),acceptor x aromatic (18.0-17.8),acceptor x hydrophobic (18.0-17.8),acceptor x negative (18.0-17.8),acceptor x neutral (18.0-17.8),acceptor x positive (18.0-17.8),acceptor x sulfide (18.0-17.8),donor x donor (18.0-17.8),donor x aromatic (18.0-17.8),donor x hydrophobic (18.0-17.8),donor x negative (18.0-17.8),donor x neutral (18.0-17.8),donor x positive (18.0-17.8),donor x sulfide (18.0-17.8),aromatic x aromatic (18.0-17.8),aromatic x hydrophobic (18.0-17.8),aromatic x negative (18.0-17.8),aromatic x neutral (18.0-17.8),aromatic x positive (18.0-17.8),aromatic x sulfide (18.0-17.8),hydrophobic x hydrophobic (18.0-17.8),hydrophobic x negative (18.0-17.8),hydrophobic x neutral (18.0-17.8),hydrophobic x positive (18.0-17.8),hydrophobic x sulfide (18.0-17.8),negative x negative (18.0-17.8),negative x neutral (18.0-17.8),negative x positive (18.0-17.8),negative x sulfide (18.0-17.8),neutral x neutral (18.0-17.8),neutral x positive (18.0-17.8),neutral x sulfide (18.0-17.8),positive x positive (18.0-17.8),positive x sulfide (18.0-17.8),sulfide x sulfide (18.0-17.8),acceptor x acceptor (17.8-17.6),acceptor x donor (17.8-17.6),acceptor x aromatic (17.8-17.6),acceptor x hydrophobic (17.8-17.6),acceptor x negative (17.8-17.6),acceptor x neutral (17.8-17.6),acceptor x positive (17.8-17.6),acceptor x sulfide (17.8-17.6),donor x donor (17.8-17.6),donor x aromatic (17.8-17.6),donor x hydrophobic (17.8-17.6),donor x negative (17.8-17.6),donor x neutral (17.8-17.6),donor x positive (17.8-17.6),donor x sulfide (17.8-17.6),aromatic x aromatic (17.8-17.6),aromatic x hydrophobic (17.8-17.6),aromatic x negative (17.8-17.6),aromatic x neutral (17.8-17.6),aromatic x positive (17.8-17.6),aromatic x sulfide (17.8-17.6),hydrophobic x hydrophobic (17.8-17.6),hydrophobic x negative (17.8-17.6),hydrophobic x neutral (17.8-17.6),hydrophobic x positive (17.8-17.6),hydrophobic x sulfide (17.8-17.6),negative x negative (17.8-17.6),negative x neutral (17.8-17.6),negative x positive (17.8-17.6),negative x sulfide (17.8-17.6),neutral x neutral (17.8-17.6),neutral x positive (17.8-17.6),neutral x sulfide (17.8-17.6),positive x positive (17.8-17.6),positive x sulfide (17.8-17.6),sulfide x sulfide (17.8-17.6),acceptor x acceptor (17.6-17.4),acceptor x donor (17.6-17.4),acceptor x aromatic (17.6-17.4),acceptor x hydrophobic (17.6-17.4),acceptor x negative (17.6-17.4),acceptor x neutral (17.6-17.4),acceptor x positive (17.6-17.4),acceptor x sulfide (17.6-17.4),donor x donor (17.6-17.4),donor x aromatic (17.6-17.4),donor x hydrophobic (17.6-17.4),donor x negative (17.6-17.4),donor x neutral (17.6-17.4),donor x positive (17.6-17.4),donor x sulfide (17.6-17.4),aromatic x aromatic (17.6-17.4),aromatic x hydrophobic (17.6-17.4),aromatic x negative (17.6-17.4),aromatic x neutral (17.6-17.4),aromatic x positive (17.6-17.4),aromatic x sulfide (17.6-17.4),hydrophobic x hydrophobic (17.6-17.4),hydrophobic x negative (17.6-17.4),hydrophobic x neutral (17.6-17.4),hydrophobic x positive (17.6-17.4),hydrophobic x sulfide (17.6-17.4),negative x negative (17.6-17.4),negative x neutral (17.6-17.4),negative x positive (17.6-17.4),negative x sulfide (17.6-17.4),neutral x neutral (17.6-17.4),neutral x positive (17.6-17.4),neutral x sulfide (17.6-17.4),positive x positive (17.6-17.4),positive x sulfide (17.6-17.4),sulfide x sulfide (17.6-17.4),acceptor x acceptor (17.4-17.2),acceptor x donor (17.4-17.2),acceptor x aromatic (17.4-17.2),acceptor x hydrophobic (17.4-17.2),acceptor x negative (17.4-17.2),acceptor x neutral (17.4-17.2),acceptor x positive (17.4-17.2),acceptor x sulfide (17.4-17.2),donor x donor (17.4-17.2),donor x aromatic (17.4-17.2),donor x hydrophobic (17.4-17.2),donor x negative (17.4-17.2),donor x neutral (17.4-17.2),donor x positive (17.4-17.2),donor x sulfide (17.4-17.2),aromatic x aromatic (17.4-17.2),aromatic x hydrophobic (17.4-17.2),aromatic x negative (17.4-17.2),aromatic x neutral (17.4-17.2),aromatic x positive (17.4-17.2),aromatic x sulfide (17.4-17.2),hydrophobic x hydrophobic (17.4-17.2),hydrophobic x negative (17.4-17.2),hydrophobic x neutral (17.4-17.2),hydrophobic x positive (17.4-17.2),hydrophobic x sulfide (17.4-17.2),negative x negative (17.4-17.2),negative x neutral (17.4-17.2),negative x positive (17.4-17.2),negative x sulfide (17.4-17.2),neutral x neutral (17.4-17.2),neutral x positive (17.4-17.2),neutral x sulfide (17.4-17.2),positive x positive (17.4-17.2),positive x sulfide (17.4-17.2),sulfide x sulfide (17.4-17.2),acceptor x acceptor (17.2-17.0),acceptor x donor (17.2-17.0),acceptor x aromatic (17.2-17.0),acceptor x hydrophobic (17.2-17.0),acceptor x negative (17.2-17.0),acceptor x neutral (17.2-17.0),acceptor x positive (17.2-17.0),acceptor x sulfide (17.2-17.0),donor x donor (17.2-17.0),donor x aromatic (17.2-17.0),donor x hydrophobic (17.2-17.0),donor x negative (17.2-17.0),donor x neutral (17.2-17.0),donor x positive (17.2-17.0),donor x sulfide (17.2-17.0),aromatic x aromatic (17.2-17.0),aromatic x hydrophobic (17.2-17.0),aromatic x negative (17.2-17.0),aromatic x neutral (17.2-17.0),aromatic x positive (17.2-17.0),aromatic x sulfide (17.2-17.0),hydrophobic x hydrophobic (17.2-17.0),hydrophobic x negative (17.2-17.0),hydrophobic x neutral (17.2-17.0),hydrophobic x positive (17.2-17.0),hydrophobic x sulfide (17.2-17.0),negative x negative (17.2-17.0),negative x neutral (17.2-17.0),negative x positive (17.2-17.0),negative x sulfide (17.2-17.0),neutral x neutral (17.2-17.0),neutral x positive (17.2-17.0),neutral x sulfide (17.2-17.0),positive x positive (17.2-17.0),positive x sulfide (17.2-17.0),sulfide x sulfide (17.2-17.0),acceptor x acceptor (17.0-16.8),acceptor x donor (17.0-16.8),acceptor x aromatic (17.0-16.8),acceptor x hydrophobic (17.0-16.8),acceptor x negative (17.0-16.8),acceptor x neutral (17.0-16.8),acceptor x positive (17.0-16.8),acceptor x sulfide (17.0-16.8),donor x donor (17.0-16.8),donor x aromatic (17.0-16.8),donor x hydrophobic (17.0-16.8),donor x negative (17.0-16.8),donor x neutral (17.0-16.8),donor x positive (17.0-16.8),donor x sulfide (17.0-16.8),aromatic x aromatic (17.0-16.8),aromatic x hydrophobic (17.0-16.8),aromatic x negative (17.0-16.8),aromatic x neutral (17.0-16.8),aromatic x positive (17.0-16.8),aromatic x sulfide (17.0-16.8),hydrophobic x hydrophobic (17.0-16.8),hydrophobic x negative (17.0-16.8),hydrophobic x neutral (17.0-16.8),hydrophobic x positive (17.0-16.8),hydrophobic x sulfide (17.0-16.8),negative x negative (17.0-16.8),negative x neutral (17.0-16.8),negative x positive (17.0-16.8),negative x sulfide (17.0-16.8),neutral x neutral (17.0-16.8),neutral x positive (17.0-16.8),neutral x sulfide (17.0-16.8),positive x positive (17.0-16.8),positive x sulfide (17.0-16.8),sulfide x sulfide (17.0-16.8),acceptor x acceptor (16.8-16.6),acceptor x donor (16.8-16.6),acceptor x aromatic (16.8-16.6),acceptor x hydrophobic (16.8-16.6),acceptor x negative (16.8-16.6),acceptor x neutral (16.8-16.6),acceptor x positive (16.8-16.6),acceptor x sulfide (16.8-16.6),donor x donor (16.8-16.6),donor x aromatic (16.8-16.6),donor x hydrophobic (16.8-16.6),donor x negative (16.8-16.6),donor x neutral (16.8-16.6),donor x positive (16.8-16.6),donor x sulfide (16.8-16.6),aromatic x aromatic (16.8-16.6),aromatic x hydrophobic (16.8-16.6),aromatic x negative (16.8-16.6),aromatic x neutral

(16.8-16.6),aromatic x positive (16.8-16.6),aromatic x sulfide (16.8-16.6),hydrophobic x hydrophobic (16.8-16.6),hydrophobic x negative (16.8-16.6),hydrophobic x neutral (16.8-16.6),hydrophobic x positive (16.8-16.6),hydrophobic x sulfide (16.8-16.6),negative x negative (16.8-16.6),negative x neutral (16.8-16.6),negative x positive (16.8-16.6),negative x sulfide (16.8-16.6),neutral x neutral (16.8-16.6),neutral x positive (16.8-16.6),neutral x sulfide (16.8-16.6),positive x positive (16.8-16.6),positive x sulfide (16.8-16.6),sulfide x sulfide (16.8-16.6),acceptor x acceptor (16.6-16.4),acceptor x donor (16.6-16.4),acceptor x aromatic (16.6-16.4),acceptor x hydrophobic (16.6-16.4),acceptor x negative (16.6-16.4),acceptor x neutral (16.6-16.4),acceptor x positive (16.6-16.4),acceptor x sulfide (16.6-16.4),donor x donor (16.6-16.4),donor x aromatic (16.6-16.4),donor x hydrophobic (16.6-16.4),donor x negative (16.6-16.4),donor x neutral (16.6-16.4),donor x positive (16.6-16.4),donor x sulfide (16.6-16.4),aromatic x aromatic (16.6-16.4),aromatic x hydrophobic (16.6-16.4),aromatic x negative (16.6-16.4),aromatic x neutral (16.6-16.4),aromatic x positive (16.6-16.4),aromatic x sulfide (16.6-16.4),hydrophobic x hydrophobic (16.6-16.4),hydrophobic x negative (16.6-16.4),hydrophobic x neutral (16.6-16.4),hydrophobic x positive (16.6-16.4),hydrophobic x sulfide (16.6-16.4),negative x negative (16.6-16.4),negative x neutral (16.6-16.4),negative x positive (16.6-16.4),negative x sulfide (16.6-16.4),neutral x neutral (16.6-16.4),neutral x positive (16.6-16.4),neutral x sulfide (16.6-16.4),positive x positive (16.6-16.4),positive x sulfide (16.6-16.4),sulfide x sulfide (16.6-16.4),acceptor x acceptor (16.4-16.2),acceptor x donor (16.4-16.2),acceptor x aromatic (16.4-16.2),acceptor x hydrophobic (16.4-16.2),acceptor x negative (16.4-16.2),acceptor x neutral (16.4-16.2),acceptor x positive (16.4-16.2),acceptor x sulfide (16.4-16.2),donor x donor (16.4-16.2),donor x aromatic (16.4-16.2),donor x hydrophobic (16.4-16.2),donor x negative (16.4-16.2),donor x neutral (16.4-16.2),donor x positive (16.4-16.2),donor x sulfide (16.4-16.2),aromatic x aromatic (16.4-16.2),aromatic x hydrophobic (16.4-16.2),aromatic x negative (16.4-16.2),aromatic x neutral (16.4-16.2),aromatic x positive (16.4-16.2),aromatic x sulfide (16.4-16.2),hydrophobic x hydrophobic (16.4-16.2),hydrophobic x negative (16.4-16.2),hydrophobic x neutral (16.4-16.2),hydrophobic x positive (16.4-16.2),hydrophobic x sulfide (16.4-16.2),negative x negative (16.4-16.2),negative x neutral (16.4-16.2),negative x positive (16.4-16.2),negative x sulfide (16.4-16.2),neutral x neutral (16.4-16.2),neutral x positive (16.4-16.2),neutral x sulfide (16.4-16.2),positive x positive (16.4-16.2),positive x sulfide (16.4-16.2),sulfide x sulfide (16.4-16.2),acceptor x acceptor (16.2-16.0),acceptor x donor (16.2-16.0),acceptor x aromatic (16.2-16.0),acceptor x hydrophobic (16.2-16.0),acceptor x negative (16.2-16.0),acceptor x neutral (16.2-16.0),acceptor x positive (16.2-16.0),acceptor x sulfide (16.2-16.0),donor x donor (16.2-16.0),donor x aromatic (16.2-16.0),donor x hydrophobic (16.2-16.0),donor x negative (16.2-16.0),donor x neutral (16.2-16.0),donor x positive (16.2-16.0),donor x sulfide (16.2-16.0),aromatic x aromatic (16.2-16.0),aromatic x hydrophobic (16.2-16.0),aromatic x negative (16.2-16.0),aromatic x neutral (16.2-16.0),aromatic x positive (16.2-16.0),aromatic x sulfide (16.2-16.0),hydrophobic x hydrophobic (16.2-16.0),hydrophobic x negative (16.2-16.0),hydrophobic x neutral (16.2-16.0),hydrophobic x positive (16.2-16.0),hydrophobic x sulfide (16.2-16.0),negative x negative (16.2-16.0),negative x neutral (16.2-16.0),negative x positive (16.2-16.0),negative x sulfide (16.2-16.0),neutral x neutral (16.2-16.0),neutral x positive (16.2-16.0),neutral x sulfide (16.2-16.0),positive x positive (16.2-16.0),positive x sulfide (16.2-16.0),sulfide x sulfide (16.2-16.0),acceptor x acceptor (16.0-15.8),acceptor x donor (16.0-15.8),acceptor x aromatic (16.0-15.8),acceptor x hydrophobic (16.0-15.8),acceptor x negative (16.0-15.8),acceptor x neutral (16.0-15.8),acceptor x positive (16.0-15.8),acceptor x sulfide (16.0-15.8),donor x donor (16.0-15.8),donor x aromatic (16.0-15.8),donor x hydrophobic (16.0-15.8),donor x negative (16.0-15.8),donor x neutral (16.0-15.8),donor x positive (16.0-15.8),donor x sulfide (16.0-15.8),aromatic x aromatic (16.0-15.8),aromatic x hydrophobic (16.0-15.8),aromatic x negative (16.0-15.8),aromatic x neutral (16.0-15.8),aromatic x positive (16.0-15.8),aromatic x sulfide (16.0-15.8),hydrophobic x hydrophobic (16.0-15.8),hydrophobic x negative (16.0-15.8),hydrophobic x neutral (16.0-15.8),hydrophobic x positive (16.0-15.8),hydrophobic x sulfide (16.0-15.8),negative x negative (16.0-15.8),negative x neutral (16.0-15.8),negative x positive (16.0-15.8),negative x sulfide (16.0-15.8),neutral x neutral (16.0-15.8),neutral x positive (16.0-15.8),neutral x sulfide (16.0-15.8),positive x positive (16.0-15.8),positive x sulfide (16.0-15.8),sulfide x sulfide (16.0-15.8),acceptor x acceptor (15.8-15.6),acceptor x donor (15.8-15.6),acceptor x aromatic (15.8-15.6),acceptor x hydrophobic (15.8-15.6),acceptor x negative (15.8-15.6),acceptor x neutral (15.8-15.6),acceptor x positive (15.8-15.6),acceptor x sulfide (15.8-15.6),donor x donor (15.8-15.6),donor x aromatic (15.8-15.6),donor x hydrophobic (15.8-15.6),donor x negative (15.8-15.6),donor x neutral (15.8-15.6),donor x positive (15.8-15.6),donor x sulfide (15.8-15.6),aromatic x aromatic (15.8-15.6),aromatic x hydrophobic (15.8-15.6),aromatic x negative (15.8-15.6),aromatic x neutral (15.8-15.6),aromatic x positive (15.8-15.6),aromatic x sulfide (15.8-15.6),hydrophobic x hydrophobic (15.8-15.6),hydrophobic x negative (15.8-15.6),hydrophobic x neutral (15.8-15.6),hydrophobic x positive (15.8-15.6),hydrophobic x sulfide (15.8-15.6),negative x negative (15.8-15.6),negative x neutral (15.8-15.6),negative x positive (15.8-15.6),negative x sulfide (15.8-15.6),neutral x neutral (15.8-15.6),neutral x positive (15.8-15.6),neutral x sulfide (15.8-15.6),positive x positive (15.8-15.6),positive x sulfide (15.8-15.6),sulfide x sulfide (15.8-15.6),acceptor x acceptor (15.6-15.4),acceptor x donor (15.6-15.4),acceptor x aromatic (15.6-15.4),acceptor x hydrophobic (15.6-15.4),acceptor x negative (15.6-15.4),acceptor x neutral (15.6-15.4),acceptor x positive (15.6-15.4),acceptor x sulfide (15.6-15.4),donor x donor (15.6-15.4),donor x aromatic (15.6-15.4),donor x hydrophobic (15.6-15.4),donor x negative (15.6-15.4),donor x neutral (15.6-15.4),donor x positive (15.6-15.4),donor x sulfide (15.6-15.4),aromatic x aromatic (15.6-15.4),aromatic x hydrophobic (15.6-15.4),aromatic x negative (15.6-15.4),aromatic x neutral (15.6-15.4),aromatic x positive (15.6-15.4),aromatic x sulfide (15.6-15.4),hydrophobic x hydrophobic (15.6-15.4),hydrophobic x negative (15.6-15.4),hydrophobic x neutral (15.6-15.4),hydrophobic x positive (15.6-15.4),hydrophobic x sulfide (15.6-15.4),negative x negative (15.6-15.4),negative x neutral (15.6-15.4),negative x positive (15.6-15.4),negative x sulfide (15.6-15.4),neutral x neutral (15.6-15.4),neutral x positive (15.6-15.4),neutral x sulfide (15.6-15.4),positive x positive (15.6-15.4),positive x sulfide (15.6-15.4),sulfide x sulfide (15.6-15.4),acceptor x acceptor (15.4-15.2),acceptor x donor (15.4-15.2),acceptor x aromatic (15.4-15.2),acceptor x hydrophobic (15.4-15.2),acceptor x negative (15.4-15.2),acceptor x neutral (15.4-15.2),acceptor x positive (15.4-15.2),acceptor x sulfide (15.4-15.2),donor x donor (15.4-15.2),donor x aromatic (15.4-15.2),donor x hydrophobic (15.4-15.2),donor x negative (15.4-15.2),donor x neutral (15.4-15.2),donor x positive (15.4-15.2),donor x sulfide (15.4-15.2),aromatic x aromatic (15.4-15.2),aromatic x hydrophobic (15.4-15.2),aromatic x negative (15.4-15.2),aromatic x neutral (15.4-15.2),aromatic x positive (15.4-15.2),aromatic x sulfide (15.4-15.2),hydrophobic x hydrophobic (15.4-15.2),hydrophobic x negative (15.4-15.2),hydrophobic x neutral (15.4-15.2),hydrophobic x positive (15.4-15.2),hydrophobic x sulfide (15.4-15.2),negative x negative (15.4-15.2),negative x neutral (15.4-15.2),negative x positive (15.4-15.2),negative x sulfide (15.4-15.2),neutral x neutral (15.4-15.2),neutral x positive (15.4-15.2),neutral x sulfide (15.4-15.2),positive x positive (15.4-15.2),positive x sulfide (15.4-15.2),sulfide x sulfide (15.4-15.2),acceptor x acceptor (15.2-15.0),acceptor x donor (15.2-15.0),acceptor x aromatic (15.2-15.0),acceptor x hydrophobic (15.2-15.0),acceptor x negative (15.2-15.0),acceptor x neutral (15.2-15.0),acceptor x positive (15.2-15.0),acceptor x sulfide (15.2-15.0),donor x donor (15.2-15.0),donor x aromatic (15.2-15.0),donor x hydrophobic (15.2-15.0),donor x negative (15.2-15.0),donor x neutral (15.2-15.0),donor x positive (15.2-15.0),donor x sulfide (15.2-15.0),aromatic x aromatic (15.2-15.0),aromatic x hydrophobic (15.2-15.0),aromatic x negative (15.2-15.0),aromatic x neutral (15.2-15.0),aromatic x positive (15.2-15.0),aromatic x sulfide (15.2-15.0),hydrophobic x hydrophobic (15.2-15.0),hydrophobic x negative (15.2-15.0),hydrophobic x neutral (15.2-15.0),hydrophobic x positive (15.2-15.0),hydrophobic x sulfide (15.2-15.0),negative x negative (15.2-15.0),negative x neutral (15.2-15.0),negative x positive (15.2-15.0),negative x sulfide (15.2-15.0),neutral x neutral (15.2-15.0),neutral x

positive (15.2-15.0),neutral x sulfide (15.2-15.0),positive x positive (15.2-15.0),positive x sulfide (15.2-15.0),sulfide x sulfide (15.2-15.0),acceptor x
acceptor (15.0-14.8),acceptor x donor (15.0-14.8),acceptor x aromatic (15.0-14.8),acceptor x hydrophobic (15.0-14.8),acceptor x negative
(15.0-14.8),acceptor x neutral (15.0-14.8),acceptor x positive (15.0-14.8),acceptor x sulfide (15.0-14.8),donor x donor (15.0-14.8),donor x aromatic
(15.0-14.8),donor x hydrophobic (15.0-14.8),donor x negative (15.0-14.8),donor x neutral (15.0-14.8),donor x positive (15.0-14.8),donor x sulfide
(15.0-14.8),aromatic x aromatic (15.0-14.8),aromatic x hydrophobic (15.0-14.8),aromatic x negative (15.0-14.8),aromatic x neutral
(15.0-14.8),aromatic x positive (15.0-14.8),aromatic x sulfide (15.0-14.8),hydrophobic x hydrophobic (15.0-14.8),hydrophobic x negative
(15.0-14.8),hydrophobic x neutral (15.0-14.8),hydrophobic x positive (15.0-14.8),hydrophobic x sulfide (15.0-14.8),negative x negative
(15.0-14.8),negative x neutral (15.0-14.8),negative x positive (15.0-14.8),negative x sulfide (15.0-14.8),neutral x neutral (15.0-14.8),neutral x
positive (15.0-14.8),neutral x sulfide (15.0-14.8),positive x positive (15.0-14.8),positive x sulfide (15.0-14.8),sulfide x sulfide (15.0-14.8),acceptor x
acceptor (14.8-14.6),acceptor x donor (14.8-14.6),acceptor x aromatic (14.8-14.6),acceptor x hydrophobic (14.8-14.6),acceptor x negative
(14.8-14.6),acceptor x neutral (14.8-14.6),acceptor x positive (14.8-14.6),acceptor x sulfide (14.8-14.6),donor x donor (14.8-14.6),donor x aromatic
(14.8-14.6),donor x hydrophobic (14.8-14.6),donor x negative (14.8-14.6),donor x neutral (14.8-14.6),donor x positive (14.8-14.6),donor x sulfide
(14.8-14.6),aromatic x aromatic (14.8-14.6),aromatic x hydrophobic (14.8-14.6),aromatic x negative (14.8-14.6),aromatic x neutral
(14.8-14.6),aromatic x positive (14.8-14.6),aromatic x sulfide (14.8-14.6),hydrophobic x hydrophobic (14.8-14.6),hydrophobic x negative
(14.8-14.6),hydrophobic x neutral (14.8-14.6),hydrophobic x positive (14.8-14.6),hydrophobic x sulfide (14.8-14.6),negative x negative
(14.8-14.6),negative x neutral (14.8-14.6),negative x positive (14.8-14.6),negative x sulfide (14.8-14.6),neutral x neutral (14.8-14.6),neutral x
positive (14.8-14.6),neutral x sulfide (14.8-14.6),positive x positive (14.8-14.6),positive x sulfide (14.8-14.6),sulfide x sulfide (14.8-14.6),acceptor x
acceptor (14.6-14.4),acceptor x donor (14.6-14.4),acceptor x aromatic (14.6-14.4),acceptor x hydrophobic (14.6-14.4),acceptor x negative
(14.6-14.4),acceptor x neutral (14.6-14.4),acceptor x positive (14.6-14.4),acceptor x sulfide (14.6-14.4),donor x donor (14.6-14.4),donor x aromatic
(14.6-14.4),donor x hydrophobic (14.6-14.4),donor x negative (14.6-14.4),donor x neutral (14.6-14.4),donor x positive (14.6-14.4),donor x sulfide
(14.6-14.4),aromatic x aromatic (14.6-14.4),aromatic x hydrophobic (14.6-14.4),aromatic x negative (14.6-14.4),aromatic x neutral
(14.6-14.4),aromatic x positive (14.6-14.4),aromatic x sulfide (14.6-14.4),hydrophobic x hydrophobic (14.6-14.4),hydrophobic x negative
(14.6-14.4),hydrophobic x neutral (14.6-14.4),hydrophobic x positive (14.6-14.4),hydrophobic x sulfide (14.6-14.4),negative x negative
(14.6-14.4),negative x neutral (14.6-14.4),negative x positive (14.6-14.4),negative x sulfide (14.6-14.4),neutral x neutral (14.6-14.4),neutral x
positive (14.6-14.4),neutral x sulfide (14.6-14.4),positive x positive (14.6-14.4),positive x sulfide (14.6-14.4),sulfide x sulfide (14.6-14.4),acceptor x
acceptor (14.4-14.2),acceptor x donor (14.4-14.2),acceptor x aromatic (14.4-14.2),acceptor x hydrophobic (14.4-14.2),acceptor x negative
(14.4-14.2),acceptor x neutral (14.4-14.2),acceptor x positive (14.4-14.2),acceptor x sulfide (14.4-14.2),donor x donor (14.4-14.2),donor x aromatic
(14.4-14.2),donor x hydrophobic (14.4-14.2),donor x negative (14.4-14.2),donor x neutral (14.4-14.2),donor x positive (14.4-14.2),donor x sulfide
(14.4-14.2),aromatic x aromatic (14.4-14.2),aromatic x hydrophobic (14.4-14.2),aromatic x negative (14.4-14.2),aromatic x neutral
(14.4-14.2),aromatic x positive (14.4-14.2),aromatic x sulfide (14.4-14.2),hydrophobic x hydrophobic (14.4-14.2),hydrophobic x negative
(14.4-14.2),hydrophobic x neutral (14.4-14.2),hydrophobic x positive (14.4-14.2),hydrophobic x sulfide (14.4-14.2),negative x negative
(14.4-14.2),negative x neutral (14.4-14.2),negative x positive (14.4-14.2),negative x sulfide (14.4-14.2),neutral x neutral (14.4-14.2),neutral x
positive (14.4-14.2),neutral x sulfide (14.4-14.2),positive x positive (14.4-14.2),positive x sulfide (14.4-14.2),sulfide x sulfide (14.4-14.2),acceptor x
acceptor (14.2-14.0),acceptor x donor (14.2-14.0),acceptor x aromatic (14.2-14.0),acceptor x hydrophobic (14.2-14.0),acceptor x negative
(14.2-14.0),acceptor x neutral (14.2-14.0),acceptor x positive (14.2-14.0),acceptor x sulfide (14.2-14.0),donor x donor (14.2-14.0),donor x aromatic
(14.2-14.0),donor x hydrophobic (14.2-14.0),donor x negative (14.2-14.0),donor x neutral (14.2-14.0),donor x positive (14.2-14.0),donor x sulfide
(14.2-14.0),aromatic x aromatic (14.2-14.0),aromatic x hydrophobic (14.2-14.0),aromatic x negative (14.2-14.0),aromatic x neutral
(14.2-14.0),aromatic x positive (14.2-14.0),aromatic x sulfide (14.2-14.0),hydrophobic x hydrophobic (14.2-14.0),hydrophobic x negative
(14.2-14.0),hydrophobic x neutral (14.2-14.0),hydrophobic x positive (14.2-14.0),hydrophobic x sulfide (14.2-14.0),negative x negative
(14.2-14.0),negative x neutral (14.2-14.0),negative x positive (14.2-14.0),negative x sulfide (14.2-14.0),neutral x neutral (14.2-14.0),neutral x
positive (14.2-14.0),neutral x sulfide (14.2-14.0),positive x positive (14.2-14.0),positive x sulfide (14.2-14.0),sulfide x sulfide (14.2-14.0),acceptor x
acceptor (14.0-13.8),acceptor x donor (14.0-13.8),acceptor x aromatic (14.0-13.8),acceptor x hydrophobic (14.0-13.8),acceptor x negative
(14.0-13.8),acceptor x neutral (14.0-13.8),acceptor x positive (14.0-13.8),acceptor x sulfide (14.0-13.8),donor x donor (14.0-13.8),donor x aromatic
(14.0-13.8),donor x hydrophobic (14.0-13.8),donor x negative (14.0-13.8),donor x neutral (14.0-13.8),donor x positive (14.0-13.8),donor x sulfide
(14.0-13.8),aromatic x aromatic (14.0-13.8),aromatic x hydrophobic (14.0-13.8),aromatic x negative (14.0-13.8),aromatic x neutral
(14.0-13.8),aromatic x positive (14.0-13.8),aromatic x sulfide (14.0-13.8),hydrophobic x hydrophobic (14.0-13.8),hydrophobic x negative
(14.0-13.8),hydrophobic x neutral (14.0-13.8),hydrophobic x positive (14.0-13.8),hydrophobic x sulfide (14.0-13.8),negative x negative
(14.0-13.8),negative x neutral (14.0-13.8),negative x positive (14.0-13.8),negative x sulfide (14.0-13.8),neutral x neutral (14.0-13.8),neutral x
positive (14.0-13.8),neutral x sulfide (14.0-13.8),positive x positive (14.0-13.8),positive x sulfide (14.0-13.8),sulfide x sulfide (14.0-13.8),acceptor x
acceptor (13.8-13.6),acceptor x donor (13.8-13.6),acceptor x aromatic (13.8-13.6),acceptor x hydrophobic (13.8-13.6),acceptor x negative
(13.8-13.6),acceptor x neutral (13.8-13.6),acceptor x positive (13.8-13.6),acceptor x sulfide (13.8-13.6),donor x donor (13.8-13.6),donor x aromatic
(13.8-13.6),donor x hydrophobic (13.8-13.6),donor x negative (13.8-13.6),donor x neutral (13.8-13.6),donor x positive (13.8-13.6),donor x sulfide
(13.8-13.6),aromatic x aromatic (13.8-13.6),aromatic x hydrophobic (13.8-13.6),aromatic x negative (13.8-13.6),aromatic x neutral
(13.8-13.6),aromatic x positive (13.8-13.6),aromatic x sulfide (13.8-13.6),hydrophobic x hydrophobic (13.8-13.6),hydrophobic x negative
(13.8-13.6),hydrophobic x neutral (13.8-13.6),hydrophobic x positive (13.8-13.6),hydrophobic x sulfide (13.8-13.6),negative x negative
(13.8-13.6),negative x neutral (13.8-13.6),negative x positive (13.8-13.6),negative x sulfide (13.8-13.6),neutral x neutral (13.8-13.6),neutral x
positive (13.8-13.6),neutral x sulfide (13.8-13.6),positive x positive (13.8-13.6),positive x sulfide (13.8-13.6),sulfide x sulfide (13.8-13.6),acceptor x
acceptor (13.6-13.4),acceptor x donor (13.6-13.4),acceptor x aromatic (13.6-13.4),acceptor x hydrophobic (13.6-13.4),acceptor x negative
(13.6-13.4),acceptor x neutral (13.6-13.4),acceptor x positive (13.6-13.4),acceptor x sulfide (13.6-13.4),donor x donor (13.6-13.4),donor x aromatic
(13.6-13.4),donor x hydrophobic (13.6-13.4),donor x negative (13.6-13.4),donor x neutral (13.6-13.4),donor x positive (13.6-13.4),donor x sulfide
(13.6-13.4),aromatic x aromatic (13.6-13.4),aromatic x hydrophobic (13.6-13.4),aromatic x negative (13.6-13.4),aromatic x neutral
(13.6-13.4),aromatic x positive (13.6-13.4),aromatic x sulfide (13.6-13.4),hydrophobic x hydrophobic (13.6-13.4),hydrophobic x negative
(13.6-13.4),hydrophobic x neutral (13.6-13.4),hydrophobic x positive (13.6-13.4),hydrophobic x sulfide (13.6-13.4),negative x negative
(13.6-13.4),negative x neutral (13.6-13.4),negative x positive (13.6-13.4),negative x sulfide (13.6-13.4),neutral x neutral (13.6-13.4),neutral x
positive (13.6-13.4),neutral x sulfide (13.6-13.4),positive x positive (13.6-13.4),positive x sulfide (13.6-13.4),sulfide x sulfide (13.6-13.4),acceptor x
acceptor (13.4-13.2),acceptor x donor (13.4-13.2),acceptor x aromatic (13.4-13.2),acceptor x hydrophobic (13.4-13.2),acceptor x negative
(13.4-13.2),acceptor x neutral (13.4-13.2),acceptor x positive (13.4-13.2),acceptor x sulfide (13.4-13.2),donor x donor (13.4-13.2),donor x aromatic
(13.4-13.2),donor x hydrophobic (13.4-13.2),donor x negative (13.4-13.2),donor x neutral (13.4-13.2),donor x positive (13.4-13.2),donor x sulfide
(13.4-13.2),aromatic x aromatic (13.4-13.2),aromatic x hydrophobic (13.4-13.2),aromatic x negative (13.4-13.2),aromatic x neutral
(13.4-13.2),aromatic x positive (13.4-13.2),aromatic x sulfide (13.4-13.2),hydrophobic x hydrophobic (13.4-13.2),hydrophobic x negative

(13.4-13.2),hydrophobic x neutral (13.4-13.2),hydrophobic x positive (13.4-13.2),hydrophobic x sulfide (13.4-13.2),negative x negative (13.4-13.2),negative x neutral (13.4-13.2),negative x positive (13.4-13.2),negative x sulfide (13.4-13.2),neutral x neutral (13.4-13.2),neutral x positive (13.4-13.2),neutral x sulfide (13.4-13.2),positive x positive (13.4-13.2),positive x sulfide (13.4-13.2),sulfide x sulfide (13.4-13.2),acceptor x acceptor (13.2-13.0),acceptor x donor (13.2-13.0),acceptor x aromatic (13.2-13.0),acceptor x hydrophobic (13.2-13.0),acceptor x negative (13.2-13.0),acceptor x neutral (13.2-13.0),acceptor x positive (13.2-13.0),acceptor x sulfide (13.2-13.0),donor x donor (13.2-13.0),donor x aromatic (13.2-13.0),donor x hydrophobic (13.2-13.0),donor x negative (13.2-13.0),donor x neutral (13.2-13.0),donor x positive (13.2-13.0),donor x sulfide (13.2-13.0),aromatic x aromatic (13.2-13.0),aromatic x hydrophobic (13.2-13.0),aromatic x negative (13.2-13.0),aromatic x neutral (13.2-13.0),aromatic x positive (13.2-13.0),aromatic x sulfide (13.2-13.0),hydrophobic x hydrophobic (13.2-13.0),hydrophobic x negative (13.2-13.0),hydrophobic x neutral (13.2-13.0),hydrophobic x positive (13.2-13.0),hydrophobic x sulfide (13.2-13.0),negative x negative (13.2-13.0),negative x neutral (13.2-13.0),negative x positive (13.2-13.0),negative x sulfide (13.2-13.0),neutral x neutral (13.2-13.0),neutral x positive (13.2-13.0),neutral x sulfide (13.2-13.0),positive x positive (13.2-13.0),positive x sulfide (13.2-13.0),sulfide x sulfide (13.2-13.0),acceptor x acceptor (13.0-12.8),acceptor x donor (13.0-12.8),acceptor x aromatic (13.0-12.8),acceptor x hydrophobic (13.0-12.8),acceptor x negative (13.0-12.8),acceptor x neutral (13.0-12.8),acceptor x positive (13.0-12.8),acceptor x sulfide (13.0-12.8),donor x donor (13.0-12.8),donor x aromatic (13.0-12.8),donor x hydrophobic (13.0-12.8),donor x negative (13.0-12.8),donor x neutral (13.0-12.8),donor x positive (13.0-12.8),donor x sulfide (13.0-12.8),aromatic x aromatic (13.0-12.8),aromatic x hydrophobic (13.0-12.8),aromatic x negative (13.0-12.8),aromatic x neutral (13.0-12.8),aromatic x positive (13.0-12.8),aromatic x sulfide (13.0-12.8),hydrophobic x hydrophobic (13.0-12.8),hydrophobic x negative (13.0-12.8),hydrophobic x neutral (13.0-12.8),hydrophobic x positive (13.0-12.8),hydrophobic x sulfide (13.0-12.8),negative x negative (13.0-12.8),negative x neutral (13.0-12.8),negative x positive (13.0-12.8),negative x sulfide (13.0-12.8),neutral x neutral (13.0-12.8),neutral x positive (13.0-12.8),neutral x sulfide (13.0-12.8),positive x positive (13.0-12.8),positive x sulfide (13.0-12.8),sulfide x sulfide (13.0-12.8),acceptor x acceptor (12.8-12.6),acceptor x donor (12.8-12.6),acceptor x aromatic (12.8-12.6),acceptor x hydrophobic (12.8-12.6),acceptor x negative (12.8-12.6),acceptor x neutral (12.8-12.6),acceptor x positive (12.8-12.6),acceptor x sulfide (12.8-12.6),donor x donor (12.8-12.6),donor x aromatic (12.8-12.6),donor x hydrophobic (12.8-12.6),donor x negative (12.8-12.6),donor x neutral (12.8-12.6),donor x positive (12.8-12.6),donor x sulfide (12.8-12.6),aromatic x aromatic (12.8-12.6),aromatic x hydrophobic (12.8-12.6),aromatic x negative (12.8-12.6),aromatic x neutral (12.8-12.6),aromatic x positive (12.8-12.6),aromatic x sulfide (12.8-12.6),hydrophobic x hydrophobic (12.8-12.6),hydrophobic x negative (12.8-12.6),hydrophobic x neutral (12.8-12.6),hydrophobic x positive (12.8-12.6),hydrophobic x sulfide (12.8-12.6),negative x negative (12.8-12.6),negative x neutral (12.8-12.6),negative x positive (12.8-12.6),negative x sulfide (12.8-12.6),neutral x neutral (12.8-12.6),neutral x positive (12.8-12.6),neutral x sulfide (12.8-12.6),positive x positive (12.8-12.6),positive x sulfide (12.8-12.6),sulfide x sulfide (12.8-12.6),acceptor x acceptor (12.6-12.4),acceptor x donor (12.6-12.4),acceptor x aromatic (12.6-12.4),acceptor x hydrophobic (12.6-12.4),acceptor x negative (12.6-12.4),acceptor x neutral (12.6-12.4),acceptor x positive (12.6-12.4),acceptor x sulfide (12.6-12.4),donor x donor (12.6-12.4),donor x aromatic (12.6-12.4),donor x hydrophobic (12.6-12.4),donor x negative (12.6-12.4),donor x neutral (12.6-12.4),donor x positive (12.6-12.4),donor x sulfide (12.6-12.4),aromatic x aromatic (12.6-12.4),aromatic x hydrophobic (12.6-12.4),aromatic x negative (12.6-12.4),aromatic x neutral (12.6-12.4),aromatic x positive (12.6-12.4),aromatic x sulfide (12.6-12.4),hydrophobic x hydrophobic (12.6-12.4),hydrophobic x negative (12.6-12.4),hydrophobic x neutral (12.6-12.4),hydrophobic x positive (12.6-12.4),hydrophobic x sulfide (12.6-12.4),negative x negative (12.6-12.4),negative x neutral (12.6-12.4),negative x positive (12.6-12.4),negative x sulfide (12.6-12.4),neutral x neutral (12.6-12.4),neutral x positive (12.6-12.4),neutral x sulfide (12.6-12.4),positive x positive (12.6-12.4),positive x sulfide (12.6-12.4),sulfide x sulfide (12.6-12.4),acceptor x acceptor (12.4-12.2),acceptor x donor (12.4-12.2),acceptor x aromatic (12.4-12.2),acceptor x hydrophobic (12.4-12.2),acceptor x negative (12.4-12.2),acceptor x neutral (12.4-12.2),acceptor x positive (12.4-12.2),acceptor x sulfide (12.4-12.2),donor x donor (12.4-12.2),donor x aromatic (12.4-12.2),donor x hydrophobic (12.4-12.2),donor x negative (12.4-12.2),donor x neutral (12.4-12.2),donor x positive (12.4-12.2),donor x sulfide (12.4-12.2),aromatic x aromatic (12.4-12.2),aromatic x hydrophobic (12.4-12.2),aromatic x negative (12.4-12.2),aromatic x neutral (12.4-12.2),aromatic x positive (12.4-12.2),aromatic x sulfide (12.4-12.2),hydrophobic x hydrophobic (12.4-12.2),hydrophobic x negative (12.4-12.2),hydrophobic x neutral (12.4-12.2),hydrophobic x positive (12.4-12.2),hydrophobic x sulfide (12.4-12.2),negative x negative (12.4-12.2),negative x neutral (12.4-12.2),negative x positive (12.4-12.2),negative x sulfide (12.4-12.2),neutral x neutral (12.4-12.2),neutral x positive (12.4-12.2),neutral x sulfide (12.4-12.2),positive x positive (12.4-12.2),positive x sulfide (12.4-12.2),sulfide x sulfide (12.4-12.2),acceptor x acceptor (12.2-12.0),acceptor x donor (12.2-12.0),acceptor x aromatic (12.2-12.0),acceptor x hydrophobic (12.2-12.0),acceptor x negative (12.2-12.0),acceptor x neutral (12.2-12.0),acceptor x positive (12.2-12.0),acceptor x sulfide (12.2-12.0),donor x donor (12.2-12.0),donor x aromatic (12.2-12.0),donor x hydrophobic (12.2-12.0),donor x negative (12.2-12.0),donor x neutral (12.2-12.0),donor x positive (12.2-12.0),donor x sulfide (12.2-12.0),aromatic x aromatic (12.2-12.0),aromatic x hydrophobic (12.2-12.0),aromatic x negative (12.2-12.0),aromatic x neutral (12.2-12.0),aromatic x positive (12.2-12.0),aromatic x sulfide (12.2-12.0),hydrophobic x hydrophobic (12.2-12.0),hydrophobic x negative (12.2-12.0),hydrophobic x neutral (12.2-12.0),hydrophobic x positive (12.2-12.0),hydrophobic x sulfide (12.2-12.0),negative x negative (12.2-12.0),negative x neutral (12.2-12.0),negative x positive (12.2-12.0),negative x sulfide (12.2-12.0),neutral x neutral (12.2-12.0),neutral x positive (12.2-12.0),neutral x sulfide (12.2-12.0),positive x positive (12.2-12.0),positive x sulfide (12.2-12.0),sulfide x sulfide (12.2-12.0),acceptor x acceptor (12.0-11.8),acceptor x donor (12.0-11.8),acceptor x aromatic (12.0-11.8),acceptor x hydrophobic (12.0-11.8),acceptor x negative (12.0-11.8),acceptor x neutral (12.0-11.8),acceptor x positive (12.0-11.8),acceptor x sulfide (12.0-11.8),donor x donor (12.0-11.8),donor x aromatic (12.0-11.8),donor x hydrophobic (12.0-11.8),donor x negative (12.0-11.8),donor x neutral (12.0-11.8),donor x positive (12.0-11.8),donor x sulfide (12.0-11.8),aromatic x aromatic (12.0-11.8),aromatic x hydrophobic (12.0-11.8),aromatic x negative (12.0-11.8),aromatic x neutral (12.0-11.8),aromatic x positive (12.0-11.8),aromatic x sulfide (12.0-11.8),hydrophobic x hydrophobic (12.0-11.8),hydrophobic x negative (12.0-11.8),hydrophobic x neutral (12.0-11.8),hydrophobic x positive (12.0-11.8),hydrophobic x sulfide (12.0-11.8),negative x negative (12.0-11.8),negative x neutral (12.0-11.8),negative x positive (12.0-11.8),negative x sulfide (12.0-11.8),neutral x neutral (12.0-11.8),neutral x positive (12.0-11.8),neutral x sulfide (12.0-11.8),positive x positive (12.0-11.8),positive x sulfide (12.0-11.8),sulfide x sulfide (12.0-11.8),acceptor x acceptor (11.8-11.6),acceptor x donor (11.8-11.6),acceptor x aromatic (11.8-11.6),acceptor x hydrophobic (11.8-11.6),acceptor x negative (11.8-11.6),acceptor x neutral (11.8-11.6),acceptor x positive (11.8-11.6),acceptor x sulfide (11.8-11.6),donor x donor (11.8-11.6),donor x aromatic (11.8-11.6),donor x hydrophobic (11.8-11.6),donor x negative (11.8-11.6),donor x neutral (11.8-11.6),donor x positive (11.8-11.6),donor x sulfide (11.8-11.6),aromatic x aromatic (11.8-11.6),aromatic x hydrophobic (11.8-11.6),aromatic x negative (11.8-11.6),aromatic x neutral (11.8-11.6),aromatic x positive (11.8-11.6),aromatic x sulfide (11.8-11.6),hydrophobic x hydrophobic (11.8-11.6),hydrophobic x negative (11.8-11.6),hydrophobic x neutral (11.8-11.6),hydrophobic x positive (11.8-11.6),hydrophobic x sulfide (11.8-11.6),negative x negative (11.8-11.6),negative x neutral (11.8-11.6),negative x positive (11.8-11.6),negative x sulfide (11.8-11.6),neutral x neutral (11.8-11.6),neutral x positive (11.8-11.6),neutral x sulfide (11.8-11.6),positive x positive (11.8-11.6),positive x sulfide (11.8-11.6),sulfide x sulfide (11.8-11.6),acceptor x acceptor

(11.6-11.4),acceptor x donor (11.6-11.4),acceptor x aromatic (11.6-11.4),acceptor x hydrophobic (11.6-11.4),acceptor x negative (11.6-11.4),acceptor x neutral (11.6-11.4),acceptor x positive (11.6-11.4),acceptor x sulfide (11.6-11.4),donor x donor (11.6-11.4),donor x aromatic (11.6-11.4),donor x hydrophobic (11.6-11.4),donor x negative (11.6-11.4),donor x neutral (11.6-11.4),donor x positive (11.6-11.4),donor x sulfide (11.6-11.4),aromatic x aromatic (11.6-11.4),aromatic x hydrophobic (11.6-11.4),aromatic x negative (11.6-11.4),aromatic x neutral (11.6-11.4),aromatic x positive (11.6-11.4),aromatic x sulfide (11.6-11.4),hydrophobic x hydrophobic (11.6-11.4),hydrophobic x negative (11.6-11.4),hydrophobic x neutral (11.6-11.4),hydrophobic x positive (11.6-11.4),hydrophobic x sulfide (11.6-11.4),negative x negative (11.6-11.4),negative x neutral (11.6-11.4),negative x positive (11.6-11.4),negative x sulfide (11.6-11.4),neutral x neutral (11.6-11.4),neutral x positive (11.6-11.4),neutral x sulfide (11.6-11.4),positive x positive (11.6-11.4),positive x sulfide (11.6-11.4),sulfide x sulfide (11.6-11.4),acceptor x acceptor (11.4-11.2),acceptor x donor (11.4-11.2),acceptor x aromatic (11.4-11.2),acceptor x hydrophobic (11.4-11.2),acceptor x negative (11.4-11.2),acceptor x neutral (11.4-11.2),acceptor x positive (11.4-11.2),acceptor x sulfide (11.4-11.2),donor x donor (11.4-11.2),donor x aromatic (11.4-11.2),donor x hydrophobic (11.4-11.2),donor x negative (11.4-11.2),donor x neutral (11.4-11.2),donor x positive (11.4-11.2),donor x sulfide (11.4-11.2),aromatic x aromatic (11.4-11.2),aromatic x hydrophobic (11.4-11.2),aromatic x negative (11.4-11.2),aromatic x neutral (11.4-11.2),aromatic x positive (11.4-11.2),aromatic x sulfide (11.4-11.2),hydrophobic x hydrophobic (11.4-11.2),hydrophobic x negative (11.4-11.2),hydrophobic x neutral (11.4-11.2),hydrophobic x positive (11.4-11.2),hydrophobic x sulfide (11.4-11.2),negative x negative (11.4-11.2),negative x neutral (11.4-11.2),negative x positive (11.4-11.2),negative x sulfide (11.4-11.2),neutral x neutral (11.4-11.2),neutral x positive (11.4-11.2),neutral x sulfide (11.4-11.2),positive x positive (11.4-11.2),positive x sulfide (11.4-11.2),sulfide x sulfide (11.4-11.2),acceptor x acceptor (11.2-11.0),acceptor x donor (11.2-11.0),acceptor x aromatic (11.2-11.0),acceptor x hydrophobic (11.2-11.0),acceptor x negative (11.2-11.0),acceptor x neutral (11.2-11.0),acceptor x positive (11.2-11.0),acceptor x sulfide (11.2-11.0),donor x donor (11.2-11.0),donor x aromatic (11.2-11.0),donor x hydrophobic (11.2-11.0),donor x negative (11.2-11.0),donor x neutral (11.2-11.0),donor x positive (11.2-11.0),donor x sulfide (11.2-11.0),aromatic x aromatic (11.2-11.0),aromatic x hydrophobic (11.2-11.0),aromatic x negative (11.2-11.0),aromatic x neutral (11.2-11.0),aromatic x positive (11.2-11.0),aromatic x sulfide (11.2-11.0),hydrophobic x hydrophobic (11.2-11.0),hydrophobic x negative (11.2-11.0),hydrophobic x neutral (11.2-11.0),hydrophobic x positive (11.2-11.0),hydrophobic x sulfide (11.2-11.0),negative x negative (11.2-11.0),negative x neutral (11.2-11.0),negative x positive (11.2-11.0),negative x sulfide (11.2-11.0),neutral x neutral (11.2-11.0),neutral x positive (11.2-11.0),neutral x sulfide (11.2-11.0),positive x positive (11.2-11.0),positive x sulfide (11.2-11.0),sulfide x sulfide (11.2-11.0),acceptor x acceptor (11.0-10.8),acceptor x donor (11.0-10.8),acceptor x aromatic (11.0-10.8),acceptor x hydrophobic (11.0-10.8),acceptor x negative (11.0-10.8),acceptor x neutral (11.0-10.8),acceptor x positive (11.0-10.8),acceptor x sulfide (11.0-10.8),donor x donor (11.0-10.8),donor x aromatic (11.0-10.8),donor x hydrophobic (11.0-10.8),donor x negative (11.0-10.8),donor x neutral (11.0-10.8),donor x positive (11.0-10.8),donor x sulfide (11.0-10.8),aromatic x aromatic (11.0-10.8),aromatic x hydrophobic (11.0-10.8),aromatic x negative (11.0-10.8),aromatic x neutral (11.0-10.8),aromatic x positive (11.0-10.8),aromatic x sulfide (11.0-10.8),hydrophobic x hydrophobic (11.0-10.8),hydrophobic x negative (11.0-10.8),hydrophobic x neutral (11.0-10.8),hydrophobic x positive (11.0-10.8),hydrophobic x sulfide (11.0-10.8),negative x negative (11.0-10.8),negative x neutral (11.0-10.8),negative x positive (11.0-10.8),negative x sulfide (11.0-10.8),neutral x neutral (11.0-10.8),neutral x positive (11.0-10.8),neutral x sulfide (11.0-10.8),positive x positive (11.0-10.8),positive x sulfide (11.0-10.8),sulfide x sulfide (11.0-10.8),acceptor x acceptor (10.8-10.6),acceptor x donor (10.8-10.6),acceptor x aromatic (10.8-10.6),acceptor x hydrophobic (10.8-10.6),acceptor x negative (10.8-10.6),acceptor x neutral (10.8-10.6),acceptor x positive (10.8-10.6),acceptor x sulfide (10.8-10.6),donor x donor (10.8-10.6),donor x aromatic (10.8-10.6),donor x hydrophobic (10.8-10.6),donor x negative (10.8-10.6),donor x neutral (10.8-10.6),donor x positive (10.8-10.6),donor x sulfide (10.8-10.6),aromatic x aromatic (10.8-10.6),aromatic x hydrophobic (10.8-10.6),aromatic x negative (10.8-10.6),aromatic x neutral (10.8-10.6),aromatic x positive (10.8-10.6),aromatic x sulfide (10.8-10.6),hydrophobic x hydrophobic (10.8-10.6),hydrophobic x negative (10.8-10.6),hydrophobic x neutral (10.8-10.6),hydrophobic x positive (10.8-10.6),hydrophobic x sulfide (10.8-10.6),negative x negative (10.8-10.6),negative x neutral (10.8-10.6),negative x positive (10.8-10.6),negative x sulfide (10.8-10.6),neutral x neutral (10.8-10.6),neutral x positive (10.8-10.6),neutral x sulfide (10.8-10.6),positive x positive (10.8-10.6),positive x sulfide (10.8-10.6),sulfide x sulfide (10.8-10.6),acceptor x acceptor (10.6-10.4),acceptor x donor (10.6-10.4),acceptor x aromatic (10.6-10.4),acceptor x hydrophobic (10.6-10.4),acceptor x negative (10.6-10.4),acceptor x neutral (10.6-10.4),acceptor x positive (10.6-10.4),acceptor x sulfide (10.6-10.4),donor x donor (10.6-10.4),donor x aromatic (10.6-10.4),donor x hydrophobic (10.6-10.4),donor x negative (10.6-10.4),donor x neutral (10.6-10.4),donor x positive (10.6-10.4),donor x sulfide (10.6-10.4),aromatic x aromatic (10.6-10.4),aromatic x hydrophobic (10.6-10.4),aromatic x negative (10.6-10.4),aromatic x neutral (10.6-10.4),aromatic x positive (10.6-10.4),aromatic x sulfide (10.6-10.4),hydrophobic x hydrophobic (10.6-10.4),hydrophobic x negative (10.6-10.4),hydrophobic x neutral (10.6-10.4),hydrophobic x positive (10.6-10.4),hydrophobic x sulfide (10.6-10.4),negative x negative (10.6-10.4),negative x neutral (10.6-10.4),negative x positive (10.6-10.4),negative x sulfide (10.6-10.4),neutral x neutral (10.6-10.4),neutral x positive (10.6-10.4),neutral x sulfide (10.6-10.4),positive x positive (10.6-10.4),positive x sulfide (10.6-10.4),sulfide x sulfide (10.6-10.4),acceptor x acceptor (10.4-10.2),acceptor x donor (10.4-10.2),acceptor x aromatic (10.4-10.2),acceptor x hydrophobic (10.4-10.2),acceptor x negative (10.4-10.2),acceptor x neutral (10.4-10.2),acceptor x positive (10.4-10.2),acceptor x sulfide (10.4-10.2),donor x donor (10.4-10.2),donor x aromatic (10.4-10.2),donor x hydrophobic (10.4-10.2),donor x negative (10.4-10.2),donor x neutral (10.4-10.2),donor x positive (10.4-10.2),donor x sulfide (10.4-10.2),aromatic x aromatic (10.4-10.2),aromatic x hydrophobic (10.4-10.2),aromatic x negative (10.4-10.2),aromatic x neutral (10.4-10.2),aromatic x positive (10.4-10.2),aromatic x sulfide (10.4-10.2),hydrophobic x hydrophobic (10.4-10.2),hydrophobic x negative (10.4-10.2),hydrophobic x neutral (10.4-10.2),hydrophobic x positive (10.4-10.2),hydrophobic x sulfide (10.4-10.2),negative x negative (10.4-10.2),negative x neutral (10.4-10.2),negative x positive (10.4-10.2),negative x sulfide (10.4-10.2),neutral x neutral (10.4-10.2),neutral x positive (10.4-10.2),neutral x sulfide (10.4-10.2),positive x positive (10.4-10.2),positive x sulfide (10.4-10.2),sulfide x sulfide (10.4-10.2),acceptor x acceptor (10.2-10.0),acceptor x donor (10.2-10.0),acceptor x aromatic (10.2-10.0),acceptor x hydrophobic (10.2-10.0),acceptor x negative (10.2-10.0),acceptor x neutral (10.2-10.0),acceptor x positive (10.2-10.0),acceptor x sulfide (10.2-10.0),donor x donor (10.2-10.0),donor x aromatic (10.2-10.0),donor x hydrophobic (10.2-10.0),donor x negative (10.2-10.0),donor x neutral (10.2-10.0),donor x positive (10.2-10.0),donor x sulfide (10.2-10.0),aromatic x aromatic (10.2-10.0),aromatic x hydrophobic (10.2-10.0),aromatic x negative (10.2-10.0),aromatic x neutral (10.2-10.0),aromatic x positive (10.2-10.0),aromatic x sulfide (10.2-10.0),hydrophobic x hydrophobic (10.2-10.0),hydrophobic x negative (10.2-10.0),hydrophobic x neutral (10.2-10.0),hydrophobic x positive (10.2-10.0),hydrophobic x sulfide (10.2-10.0),negative x negative (10.2-10.0),negative x neutral (10.2-10.0),negative x positive (10.2-10.0),negative x sulfide (10.2-10.0),neutral x neutral (10.2-10.0),neutral x positive (10.2-10.0),neutral x sulfide (10.2-10.0),positive x positive (10.2-10.0),positive x sulfide (10.2-10.0),sulfide x sulfide (10.2-10.0),acceptor x acceptor (10.0-9.8),acceptor x donor (10.0-9.8),acceptor x aromatic (10.0-9.8),acceptor x hydrophobic (10.0-9.8),acceptor x negative (10.0-9.8),acceptor x neutral (10.0-9.8),acceptor x positive (10.0-9.8),acceptor x sulfide (10.0-9.8),donor x donor (10.0-9.8),donor x aromatic (10.0-9.8),donor x hydrophobic (10.0-9.8),donor x negative (10.0-9.8),donor x neutral (10.0-9.8),donor x positive (10.0-9.8),donor x sulfide (10.0-9.8),aromatic x aromatic (10.0-9.8),aromatic x hydrophobic (10.0-9.8),aromatic x negative (10.0-9.8),aromatic x neutral (10.0-9.8),aromatic x positive (10.0-9.8),aromatic x sulfide (10.0-9.8),hydrophobic x hydrophobic (10.0-9.8),hydrophobic x negative (10.0-9.8),hydrophobic x neutral (10.0-9.8),hydrophobic x positive (10.0-9.8),hydrophobic x sulfide (10.0-9.8),negative x negative (10.0-9.8),negative x neutral (10.0-9.8),negative x positive (10.0-9.8),negative x

sulfide (10.0-9.8),neutral x neutral (10.0-9.8),neutral x positive (10.0-9.8),neutral x sulfide (10.0-9.8),positive x positive (10.0-9.8),positive x sulfide
(10.0-9.8),sulfide x sulfide (10.0-9.8),acceptor x acceptor (9.8-9.6),acceptor x donor (9.8-9.6),acceptor x aromatic (9.8-9.6),acceptor x hydrophobic
(9.8-9.6),acceptor x negative (9.8-9.6),acceptor x neutral (9.8-9.6),acceptor x positive (9.8-9.6),acceptor x sulfide (9.8-9.6),donor x donor
(9.8-9.6),donor x aromatic (9.8-9.6),donor x hydrophobic (9.8-9.6),donor x negative (9.8-9.6),donor x neutral (9.8-9.6),donor x positive
(9.8-9.6),donor x sulfide (9.8-9.6),aromatic x aromatic (9.8-9.6),aromatic x hydrophobic (9.8-9.6),aromatic x negative (9.8-9.6),aromatic x neutral
(9.8-9.6),aromatic x positive (9.8-9.6),aromatic x sulfide (9.8-9.6),hydrophobic x hydrophobic (9.8-9.6),hydrophobic x negative
(9.8-9.6),hydrophobic x neutral (9.8-9.6),hydrophobic x positive (9.8-9.6),hydrophobic x sulfide (9.8-9.6),negative x negative (9.8-9.6),negative x
neutral (9.8-9.6),negative x positive (9.8-9.6),negative x sulfide (9.8-9.6),neutral x neutral (9.8-9.6),neutral x positive (9.8-9.6),neutral x sulfide
(9.8-9.6),positive x positive (9.8-9.6),positive x sulfide (9.8-9.6),sulfide x sulfide (9.8-9.6),acceptor x acceptor (9.6-9.4),acceptor x donor
(9.6-9.4),acceptor x aromatic (9.6-9.4),acceptor x hydrophobic (9.6-9.4),acceptor x negative (9.6-9.4),acceptor x neutral (9.6-9.4),acceptor x positive
(9.6-9.4),acceptor x sulfide (9.6-9.4),donor x donor (9.6-9.4),donor x aromatic (9.6-9.4),donor x hydrophobic (9.6-9.4),donor x negative
(9.6-9.4),donor x neutral (9.6-9.4),donor x positive (9.6-9.4),donor x sulfide (9.6-9.4),aromatic x aromatic (9.6-9.4),aromatic x hydrophobic
(9.6-9.4),aromatic x negative (9.6-9.4),aromatic x neutral (9.6-9.4),aromatic x positive (9.6-9.4),aromatic x sulfide (9.6-9.4),hydrophobic x
hydrophobic (9.6-9.4),hydrophobic x negative (9.6-9.4),hydrophobic x neutral (9.6-9.4),hydrophobic x positive (9.6-9.4),hydrophobic x sulfide
(9.6-9.4),negative x negative (9.6-9.4),negative x neutral (9.6-9.4),negative x positive (9.6-9.4),negative x sulfide (9.6-9.4),neutral x neutral
(9.6-9.4),neutral x positive (9.6-9.4),neutral x sulfide (9.6-9.4),positive x positive (9.6-9.4),positive x sulfide (9.6-9.4),sulfide x sulfide
(9.6-9.4),acceptor x acceptor (9.4-9.2),acceptor x donor (9.4-9.2),acceptor x aromatic (9.4-9.2),acceptor x hydrophobic (9.4-9.2),acceptor x negative
(9.4-9.2),acceptor x neutral (9.4-9.2),acceptor x positive (9.4-9.2),acceptor x sulfide (9.4-9.2),donor x donor (9.4-9.2),donor x aromatic
(9.4-9.2),donor x hydrophobic (9.4-9.2),donor x negative (9.4-9.2),donor x neutral (9.4-9.2),donor x positive (9.4-9.2),donor x sulfide
(9.4-9.2),aromatic x aromatic (9.4-9.2),aromatic x hydrophobic (9.4-9.2),aromatic x negative (9.4-9.2),aromatic x neutral (9.4-9.2),aromatic x
positive (9.4-9.2),aromatic x sulfide (9.4-9.2),hydrophobic x hydrophobic (9.4-9.2),hydrophobic x negative (9.4-9.2),hydrophobic x neutral
(9.4-9.2),hydrophobic x positive (9.4-9.2),hydrophobic x sulfide (9.4-9.2),negative x negative (9.4-9.2),negative x neutral (9.4-9.2),negative x
positive (9.4-9.2),negative x sulfide (9.4-9.2),neutral x neutral (9.4-9.2),neutral x positive (9.4-9.2),neutral x sulfide (9.4-9.2),positive x positive
(9.4-9.2),positive x sulfide (9.4-9.2),sulfide x sulfide (9.4-9.2),acceptor x acceptor (9.2-9.0),acceptor x donor (9.2-9.0),acceptor x aromatic
(9.2-9.0),acceptor x hydrophobic (9.2-9.0),acceptor x negative (9.2-9.0),acceptor x neutral (9.2-9.0),acceptor x positive (9.2-9.0),acceptor x sulfide
(9.2-9.0),donor x donor (9.2-9.0),donor x aromatic (9.2-9.0),donor x hydrophobic (9.2-9.0),donor x negative (9.2-9.0),donor x neutral
(9.2-9.0),donor x positive (9.2-9.0),donor x sulfide (9.2-9.0),aromatic x aromatic (9.2-9.0),aromatic x hydrophobic (9.2-9.0),aromatic x negative
(9.2-9.0),aromatic x neutral (9.2-9.0),aromatic x positive (9.2-9.0),aromatic x sulfide (9.2-9.0),hydrophobic x hydrophobic (9.2-9.0),hydrophobic x
negative (9.2-9.0),hydrophobic x neutral (9.2-9.0),hydrophobic x positive (9.2-9.0),hydrophobic x sulfide (9.2-9.0),negative x negative
(9.2-9.0),negative x neutral (9.2-9.0),negative x positive (9.2-9.0),negative x sulfide (9.2-9.0),neutral x neutral (9.2-9.0),neutral x positive
(9.2-9.0),neutral x sulfide (9.2-9.0),positive x positive (9.2-9.0),positive x sulfide (9.2-9.0),sulfide x sulfide (9.2-9.0),acceptor x acceptor
(9.0-8.8),acceptor x donor (9.0-8.8),acceptor x aromatic (9.0-8.8),acceptor x hydrophobic (9.0-8.8),acceptor x negative (9.0-8.8),acceptor x neutral
(9.0-8.8),acceptor x positive (9.0-8.8),acceptor x sulfide (9.0-8.8),donor x donor (9.0-8.8),donor x aromatic (9.0-8.8),donor x hydrophobic
(9.0-8.8),donor x negative (9.0-8.8),donor x neutral (9.0-8.8),donor x positive (9.0-8.8),donor x sulfide (9.0-8.8),aromatic x aromatic
(9.0-8.8),aromatic x hydrophobic (9.0-8.8),aromatic x negative (9.0-8.8),aromatic x neutral (9.0-8.8),aromatic x positive (9.0-8.8),aromatic x sulfide
(9.0-8.8),hydrophobic x hydrophobic (9.0-8.8),hydrophobic x negative (9.0-8.8),hydrophobic x neutral (9.0-8.8),hydrophobic x positive
(9.0-8.8),hydrophobic x sulfide (9.0-8.8),negative x negative (9.0-8.8),negative x neutral (9.0-8.8),negative x positive (9.0-8.8),negative x sulfide
(9.0-8.8),neutral x neutral (9.0-8.8),neutral x positive (9.0-8.8),neutral x sulfide (9.0-8.8),positive x positive (9.0-8.8),positive x sulfide
(9.0-8.8),sulfide x sulfide (9.0-8.8),acceptor x acceptor (8.8-8.6),acceptor x donor (8.8-8.6),acceptor x aromatic (8.8-8.6),acceptor x hydrophobic
(8.8-8.6),acceptor x negative (8.8-8.6),acceptor x neutral (8.8-8.6),acceptor x positive (8.8-8.6),acceptor x sulfide (8.8-8.6),donor x donor
(8.8-8.6),donor x aromatic (8.8-8.6),donor x hydrophobic (8.8-8.6),donor x negative (8.8-8.6),donor x neutral (8.8-8.6),donor x positive
(8.8-8.6),donor x sulfide (8.8-8.6),aromatic x aromatic (8.8-8.6),aromatic x hydrophobic (8.8-8.6),aromatic x negative (8.8-8.6),aromatic x neutral
(8.8-8.6),aromatic x positive (8.8-8.6),aromatic x sulfide (8.8-8.6),hydrophobic x hydrophobic (8.8-8.6),hydrophobic x negative
(8.8-8.6),hydrophobic x neutral (8.8-8.6),hydrophobic x positive (8.8-8.6),hydrophobic x sulfide (8.8-8.6),negative x negative (8.8-8.6),negative x
neutral (8.8-8.6),negative x positive (8.8-8.6),negative x sulfide (8.8-8.6),neutral x neutral (8.8-8.6),neutral x positive (8.8-8.6),neutral x sulfide
(8.8-8.6),positive x positive (8.8-8.6),positive x sulfide (8.8-8.6),sulfide x sulfide (8.8-8.6),acceptor x acceptor (8.6-8.4),acceptor x donor
(8.6-8.4),acceptor x aromatic (8.6-8.4),acceptor x hydrophobic (8.6-8.4),acceptor x negative (8.6-8.4),acceptor x neutral (8.6-8.4),acceptor x positive
(8.6-8.4),acceptor x sulfide (8.6-8.4),donor x donor (8.6-8.4),donor x aromatic (8.6-8.4),donor x hydrophobic (8.6-8.4),donor x negative
(8.6-8.4),donor x neutral (8.6-8.4),donor x positive (8.6-8.4),donor x sulfide (8.6-8.4),aromatic x aromatic (8.6-8.4),aromatic x hydrophobic
(8.6-8.4),aromatic x negative (8.6-8.4),aromatic x neutral (8.6-8.4),aromatic x positive (8.6-8.4),aromatic x sulfide (8.6-8.4),hydrophobic x
hydrophobic (8.6-8.4),hydrophobic x negative (8.6-8.4),hydrophobic x neutral (8.6-8.4),hydrophobic x positive (8.6-8.4),hydrophobic x sulfide
(8.6-8.4),negative x negative (8.6-8.4),negative x neutral (8.6-8.4),negative x positive (8.6-8.4),negative x sulfide (8.6-8.4),neutral x neutral
(8.6-8.4),neutral x positive (8.6-8.4),neutral x sulfide (8.6-8.4),positive x positive (8.6-8.4),positive x sulfide (8.6-8.4),sulfide x sulfide
(8.6-8.4),acceptor x acceptor (8.4-8.2),acceptor x donor (8.4-8.2),acceptor x aromatic (8.4-8.2),acceptor x hydrophobic (8.4-8.2),acceptor x negative
(8.4-8.2),acceptor x neutral (8.4-8.2),acceptor x positive (8.4-8.2),acceptor x sulfide (8.4-8.2),donor x donor (8.4-8.2),donor x aromatic
(8.4-8.2),donor x hydrophobic (8.4-8.2),donor x negative (8.4-8.2),donor x neutral (8.4-8.2),donor x positive (8.4-8.2),donor x sulfide
(8.4-8.2),aromatic x aromatic (8.4-8.2),aromatic x hydrophobic (8.4-8.2),aromatic x negative (8.4-8.2),aromatic x neutral (8.4-8.2),aromatic x
positive (8.4-8.2),aromatic x sulfide (8.4-8.2),hydrophobic x hydrophobic (8.4-8.2),hydrophobic x negative (8.4-8.2),hydrophobic x neutral
(8.4-8.2),hydrophobic x positive (8.4-8.2),hydrophobic x sulfide (8.4-8.2),negative x negative (8.4-8.2),negative x neutral (8.4-8.2),negative x
positive (8.4-8.2),negative x sulfide (8.4-8.2),neutral x neutral (8.4-8.2),neutral x positive (8.4-8.2),neutral x sulfide (8.4-8.2),positive x positive
(8.4-8.2),positive x sulfide (8.4-8.2),sulfide x sulfide (8.4-8.2),acceptor x acceptor (8.2-8.0),acceptor x donor (8.2-8.0),acceptor x aromatic
(8.2-8.0),acceptor x hydrophobic (8.2-8.0),acceptor x negative (8.2-8.0),acceptor x neutral (8.2-8.0),acceptor x positive (8.2-8.0),acceptor x sulfide
(8.2-8.0),donor x donor (8.2-8.0),donor x aromatic (8.2-8.0),donor x hydrophobic (8.2-8.0),donor x negative (8.2-8.0),donor x neutral
(8.2-8.0),donor x positive (8.2-8.0),donor x sulfide (8.2-8.0),aromatic x aromatic (8.2-8.0),aromatic x hydrophobic (8.2-8.0),aromatic x negative
(8.2-8.0),aromatic x neutral (8.2-8.0),aromatic x positive (8.2-8.0),aromatic x sulfide (8.2-8.0),hydrophobic x hydrophobic (8.2-8.0),hydrophobic x
negative (8.2-8.0),hydrophobic x neutral (8.2-8.0),hydrophobic x positive (8.2-8.0),hydrophobic x sulfide (8.2-8.0),negative x negative
(8.2-8.0),negative x neutral (8.2-8.0),negative x positive (8.2-8.0),negative x sulfide (8.2-8.0),neutral x neutral (8.2-8.0),neutral x positive

(8.2-8.0),neutral x sulfide (8.2-8.0),positive x positive (8.2-8.0),positive x sulfide (8.2-8.0),sulfide x sulfide (8.2-8.0),acceptor x acceptor (8.0-7.8),acceptor x donor (8.0-7.8),acceptor x aromatic (8.0-7.8),acceptor x hydrophobic (8.0-7.8),acceptor x negative (8.0-7.8),acceptor x neutral (8.0-7.8),acceptor x positive (8.0-7.8),acceptor x sulfide (8.0-7.8),donor x donor (8.0-7.8),donor x aromatic (8.0-7.8),donor x hydrophobic (8.0-7.8),donor x negative (8.0-7.8),donor x neutral (8.0-7.8),donor x positive (8.0-7.8),donor x sulfide (8.0-7.8),aromatic x aromatic (8.0-7.8),aromatic x hydrophobic (8.0-7.8),aromatic x negative (8.0-7.8),aromatic x neutral (8.0-7.8),aromatic x positive (8.0-7.8),aromatic x sulfide (8.0-7.8),hydrophobic x hydrophobic (8.0-7.8),hydrophobic x negative (8.0-7.8),hydrophobic x neutral (8.0-7.8),hydrophobic x positive (8.0-7.8),hydrophobic x sulfide (8.0-7.8),negative x negative (8.0-7.8),negative x neutral (8.0-7.8),negative x positive (8.0-7.8),negative x sulfide (8.0-7.8),neutral x neutral (8.0-7.8),neutral x positive (8.0-7.8),neutral x sulfide (8.0-7.8),positive x positive (8.0-7.8),positive x sulfide (8.0-7.8),sulfide x sulfide (8.0-7.8),acceptor x acceptor (7.8-7.6),acceptor x donor (7.8-7.6),acceptor x aromatic (7.8-7.6),acceptor x hydrophobic (7.8-7.6),acceptor x negative (7.8-7.6),acceptor x neutral (7.8-7.6),acceptor x positive (7.8-7.6),acceptor x sulfide (7.8-7.6),donor x donor (7.8-7.6),donor x aromatic (7.8-7.6),donor x hydrophobic (7.8-7.6),donor x negative (7.8-7.6),donor x neutral (7.8-7.6),donor x positive (7.8-7.6),donor x sulfide (7.8-7.6),aromatic x aromatic (7.8-7.6),aromatic x hydrophobic (7.8-7.6),aromatic x negative (7.8-7.6),aromatic x neutral (7.8-7.6),aromatic x positive (7.8-7.6),aromatic x sulfide (7.8-7.6),hydrophobic x hydrophobic (7.8-7.6),hydrophobic x negative (7.8-7.6),hydrophobic x neutral (7.8-7.6),hydrophobic x positive (7.8-7.6),hydrophobic x sulfide (7.8-7.6),negative x negative (7.8-7.6),negative x neutral (7.8-7.6),negative x positive (7.8-7.6),negative x sulfide (7.8-7.6),neutral x neutral (7.8-7.6),neutral x positive (7.8-7.6),neutral x sulfide (7.8-7.6),positive x positive (7.8-7.6),positive x sulfide (7.8-7.6),sulfide x sulfide (7.8-7.6),acceptor x acceptor (7.6-7.4),acceptor x donor (7.6-7.4),acceptor x aromatic (7.6-7.4),acceptor x hydrophobic (7.6-7.4),acceptor x negative (7.6-7.4),acceptor x neutral (7.6-7.4),acceptor x positive (7.6-7.4),acceptor x sulfide (7.6-7.4),donor x donor (7.6-7.4),donor x aromatic (7.6-7.4),donor x hydrophobic (7.6-7.4),donor x negative (7.6-7.4),donor x neutral (7.6-7.4),donor x positive (7.6-7.4),donor x sulfide (7.6-7.4),aromatic x aromatic (7.6-7.4),aromatic x hydrophobic (7.6-7.4),aromatic x negative (7.6-7.4),aromatic x neutral (7.6-7.4),aromatic x positive (7.6-7.4),aromatic x sulfide (7.6-7.4),hydrophobic x hydrophobic (7.6-7.4),hydrophobic x negative (7.6-7.4),hydrophobic x neutral (7.6-7.4),hydrophobic x positive (7.6-7.4),hydrophobic x sulfide (7.6-7.4),negative x negative (7.6-7.4),negative x neutral (7.6-7.4),negative x positive (7.6-7.4),negative x sulfide (7.6-7.4),neutral x neutral (7.6-7.4),neutral x positive (7.6-7.4),neutral x sulfide (7.6-7.4),positive x positive (7.6-7.4),positive x sulfide (7.6-7.4),sulfide x sulfide (7.6-7.4),acceptor x acceptor (7.4-7.2),acceptor x donor (7.4-7.2),acceptor x aromatic (7.4-7.2),acceptor x hydrophobic (7.4-7.2),acceptor x negative (7.4-7.2),acceptor x neutral (7.4-7.2),acceptor x positive (7.4-7.2),acceptor x sulfide (7.4-7.2),donor x donor (7.4-7.2),donor x aromatic (7.4-7.2),donor x hydrophobic (7.4-7.2),donor x negative (7.4-7.2),donor x neutral (7.4-7.2),donor x positive (7.4-7.2),donor x sulfide (7.4-7.2),aromatic x aromatic (7.4-7.2),aromatic x hydrophobic (7.4-7.2),aromatic x negative (7.4-7.2),aromatic x neutral (7.4-7.2),aromatic x positive (7.4-7.2),aromatic x sulfide (7.4-7.2),hydrophobic x hydrophobic (7.4-7.2),hydrophobic x negative (7.4-7.2),hydrophobic x neutral (7.4-7.2),hydrophobic x positive (7.4-7.2),hydrophobic x sulfide (7.4-7.2),negative x negative (7.4-7.2),negative x neutral (7.4-7.2),negative x positive (7.4-7.2),negative x sulfide (7.4-7.2),neutral x neutral (7.4-7.2),neutral x positive (7.4-7.2),neutral x sulfide (7.4-7.2),positive x positive (7.4-7.2),positive x sulfide (7.4-7.2),sulfide x sulfide (7.4-7.2),acceptor x acceptor (7.2-7.0),acceptor x donor (7.2-7.0),acceptor x aromatic (7.2-7.0),acceptor x hydrophobic (7.2-7.0),acceptor x negative (7.2-7.0),acceptor x neutral (7.2-7.0),acceptor x positive (7.2-7.0),acceptor x sulfide (7.2-7.0),donor x donor (7.2-7.0),donor x aromatic (7.2-7.0),donor x hydrophobic (7.2-7.0),donor x negative (7.2-7.0),donor x neutral (7.2-7.0),donor x positive (7.2-7.0),donor x sulfide (7.2-7.0),aromatic x aromatic (7.2-7.0),aromatic x hydrophobic (7.2-7.0),aromatic x negative (7.2-7.0),aromatic x neutral (7.2-7.0),aromatic x positive (7.2-7.0),aromatic x sulfide (7.2-7.0),hydrophobic x hydrophobic (7.2-7.0),hydrophobic x negative (7.2-7.0),hydrophobic x neutral (7.2-7.0),hydrophobic x positive (7.2-7.0),hydrophobic x sulfide (7.2-7.0),negative x negative (7.2-7.0),negative x neutral (7.2-7.0),negative x positive (7.2-7.0),negative x sulfide (7.2-7.0),neutral x neutral (7.2-7.0),neutral x positive (7.2-7.0),neutral x sulfide (7.2-7.0),positive x positive (7.2-7.0),positive x sulfide (7.2-7.0),sulfide x sulfide (7.2-7.0),acceptor x acceptor (7.0-6.8),acceptor x donor (7.0-6.8),acceptor x aromatic (7.0-6.8),acceptor x hydrophobic (7.0-6.8),acceptor x negative (7.0-6.8),acceptor x neutral (7.0-6.8),acceptor x positive (7.0-6.8),acceptor x sulfide (7.0-6.8),donor x donor (7.0-6.8),donor x aromatic (7.0-6.8),donor x hydrophobic (7.0-6.8),donor x negative (7.0-6.8),donor x neutral (7.0-6.8),donor x positive (7.0-6.8),donor x sulfide (7.0-6.8),aromatic x aromatic (7.0-6.8),aromatic x hydrophobic (7.0-6.8),aromatic x negative (7.0-6.8),aromatic x neutral (7.0-6.8),aromatic x positive (7.0-6.8),aromatic x sulfide (7.0-6.8),hydrophobic x hydrophobic (7.0-6.8),hydrophobic x negative (7.0-6.8),hydrophobic x neutral (7.0-6.8),hydrophobic x positive (7.0-6.8),hydrophobic x sulfide (7.0-6.8),negative x negative (7.0-6.8),negative x neutral (7.0-6.8),negative x positive (7.0-6.8),negative x sulfide (7.0-6.8),neutral x neutral (7.0-6.8),neutral x positive (7.0-6.8),neutral x sulfide (7.0-6.8),positive x positive (7.0-6.8),positive x sulfide (7.0-6.8),sulfide x sulfide (7.0-6.8),acceptor x acceptor (6.8-6.6),acceptor x donor (6.8-6.6),acceptor x aromatic (6.8-6.6),acceptor x hydrophobic (6.8-6.6),acceptor x negative (6.8-6.6),acceptor x neutral (6.8-6.6),acceptor x positive (6.8-6.6),acceptor x sulfide (6.8-6.6),donor x donor (6.8-6.6),donor x aromatic (6.8-6.6),donor x hydrophobic (6.8-6.6),donor x negative (6.8-6.6),donor x neutral (6.8-6.6),donor x positive (6.8-6.6),donor x sulfide (6.8-6.6),aromatic x aromatic (6.8-6.6),aromatic x hydrophobic (6.8-6.6),aromatic x negative (6.8-6.6),aromatic x neutral (6.8-6.6),aromatic x positive (6.8-6.6),aromatic x sulfide (6.8-6.6),hydrophobic x hydrophobic (6.8-6.6),hydrophobic x negative (6.8-6.6),hydrophobic x neutral (6.8-6.6),hydrophobic x positive (6.8-6.6),hydrophobic x sulfide (6.8-6.6),negative x negative (6.8-6.6),negative x neutral (6.8-6.6),negative x positive (6.8-6.6),negative x sulfide (6.8-6.6),neutral x neutral (6.8-6.6),neutral x positive (6.8-6.6),neutral x sulfide (6.8-6.6),positive x positive (6.8-6.6),positive x sulfide (6.8-6.6),sulfide x sulfide (6.8-6.6),acceptor x acceptor (6.6-6.4),acceptor x donor (6.6-6.4),acceptor x aromatic (6.6-6.4),acceptor x hydrophobic (6.6-6.4),acceptor x negative (6.6-6.4),acceptor x neutral (6.6-6.4),acceptor x positive (6.6-6.4),acceptor x sulfide (6.6-6.4),donor x donor (6.6-6.4),donor x aromatic (6.6-6.4),donor x hydrophobic (6.6-6.4),donor x negative (6.6-6.4),donor x neutral (6.6-6.4),donor x positive (6.6-6.4),donor x sulfide (6.6-6.4),aromatic x aromatic (6.6-6.4),aromatic x hydrophobic (6.6-6.4),aromatic x negative (6.6-6.4),aromatic x neutral (6.6-6.4),aromatic x positive (6.6-6.4),aromatic x sulfide (6.6-6.4),hydrophobic x hydrophobic (6.6-6.4),hydrophobic x negative (6.6-6.4),hydrophobic x neutral (6.6-6.4),hydrophobic x positive (6.6-6.4),hydrophobic x sulfide (6.6-6.4),negative x negative (6.6-6.4),negative x neutral (6.6-6.4),negative x positive (6.6-6.4),negative x sulfide (6.6-6.4),neutral x neutral (6.6-6.4),neutral x positive (6.6-6.4),neutral x sulfide (6.6-6.4),positive x positive (6.6-6.4),positive x sulfide (6.6-6.4),sulfide x sulfide (6.6-6.4),acceptor x acceptor (6.4-6.2),acceptor x donor (6.4-6.2),acceptor x aromatic (6.4-6.2),acceptor x hydrophobic (6.4-6.2),acceptor x negative (6.4-6.2),acceptor x neutral (6.4-6.2),acceptor x positive (6.4-6.2),acceptor x sulfide (6.4-6.2),donor x donor (6.4-6.2),donor x aromatic (6.4-6.2),donor x hydrophobic (6.4-6.2),donor x negative (6.4-6.2),donor x neutral (6.4-6.2),donor x positive (6.4-6.2),donor x sulfide (6.4-6.2),aromatic x aromatic (6.4-6.2),aromatic x hydrophobic (6.4-6.2),aromatic x negative (6.4-6.2),aromatic x neutral (6.4-6.2),aromatic x positive (6.4-6.2),aromatic x sulfide (6.4-6.2),hydrophobic x hydrophobic (6.4-6.2),hydrophobic x negative (6.4-6.2),hydrophobic x neutral (6.4-6.2),hydrophobic x positive (6.4-6.2),hydrophobic x sulfide (6.4-6.2),negative x negative (6.4-6.2),negative x neutral (6.4-6.2),negative x positive (6.4-6.2),negative x sulfide (6.4-6.2),neutral x neutral (6.4-6.2),neutral x positive (6.4-6.2),neutral x sulfide (6.4-6.2),positive x positive (6.4-6.2),positive x sulfide (6.4-6.2),sulfide x sulfide (6.4-6.2),acceptor x acceptor (6.2-6.0),acceptor x donor (6.2-6.0),acceptor x aromatic (6.2-6.0),acceptor x hydrophobic (6.2-6.0),acceptor x negative (6.2-6.0),acceptor x neutral (6.2-6.0),acceptor x positive (6.2-6.0),acceptor x sulfide (6.2-6.0),donor x donor (6.2-6.0),donor x aromatic (6.2-6.0),donor x hydrophobic (6.2-6.0),donor x negative (6.2-6.0),donor x neutral

(6.2-6.0),donor x positive (6.2-6.0),donor x sulfide (6.2-6.0),aromatic x aromatic (6.2-6.0),aromatic x hydrophobic (6.2-6.0),aromatic x negative (6.2-6.0),aromatic x neutral (6.2-6.0),aromatic x positive (6.2-6.0),aromatic x sulfide (6.2-6.0),hydrophobic x hydrophobic (6.2-6.0),hydrophobic x negative (6.2-6.0),hydrophobic x neutral (6.2-6.0),hydrophobic x positive (6.2-6.0),hydrophobic x sulfide (6.2-6.0),negative x negative (6.2-6.0),negative x neutral (6.2-6.0),negative x positive (6.2-6.0),negative x sulfide (6.2-6.0),neutral x neutral (6.2-6.0),neutral x positive (6.2-6.0),neutral x sulfide (6.2-6.0),positive x positive (6.2-6.0),positive x sulfide (6.2-6.0),sulfide x sulfide (6.2-6.0),acceptor x acceptor (6.0-5.8),acceptor x donor (6.0-5.8),acceptor x aromatic (6.0-5.8),acceptor x hydrophobic (6.0-5.8),acceptor x negative (6.0-5.8),acceptor x neutral (6.0-5.8),acceptor x positive (6.0-5.8),acceptor x sulfide (6.0-5.8),donor x donor (6.0-5.8),donor x aromatic (6.0-5.8),donor x hydrophobic (6.0-5.8),donor x negative (6.0-5.8),donor x neutral (6.0-5.8),donor x positive (6.0-5.8),donor x sulfide (6.0-5.8),aromatic x aromatic (6.0-5.8),aromatic x hydrophobic (6.0-5.8),aromatic x negative (6.0-5.8),aromatic x neutral (6.0-5.8),aromatic x positive (6.0-5.8),aromatic x sulfide (6.0-5.8),hydrophobic x hydrophobic (6.0-5.8),hydrophobic x negative (6.0-5.8),hydrophobic x neutral (6.0-5.8),hydrophobic x positive (6.0-5.8),hydrophobic x sulfide (6.0-5.8),negative x negative (6.0-5.8),negative x neutral (6.0-5.8),negative x positive (6.0-5.8),negative x sulfide (6.0-5.8),neutral x neutral (6.0-5.8),neutral x positive (6.0-5.8),neutral x sulfide (6.0-5.8),positive x positive (6.0-5.8),positive x sulfide (6.0-5.8),sulfide x sulfide (6.0-5.8),acceptor x acceptor (5.8-5.6),acceptor x donor (5.8-5.6),acceptor x aromatic (5.8-5.6),acceptor x hydrophobic (5.8-5.6),acceptor x negative (5.8-5.6),acceptor x neutral (5.8-5.6),acceptor x positive (5.8-5.6),acceptor x sulfide (5.8-5.6),donor x donor (5.8-5.6),donor x aromatic (5.8-5.6),donor x hydrophobic (5.8-5.6),donor x negative (5.8-5.6),donor x neutral (5.8-5.6),donor x positive (5.8-5.6),donor x sulfide (5.8-5.6),aromatic x aromatic (5.8-5.6),aromatic x hydrophobic (5.8-5.6),aromatic x negative (5.8-5.6),aromatic x neutral (5.8-5.6),aromatic x positive (5.8-5.6),aromatic x sulfide (5.8-5.6),hydrophobic x hydrophobic (5.8-5.6),hydrophobic x negative (5.8-5.6),hydrophobic x neutral (5.8-5.6),hydrophobic x positive (5.8-5.6),hydrophobic x sulfide (5.8-5.6),negative x negative (5.8-5.6),negative x neutral (5.8-5.6),negative x positive (5.8-5.6),negative x sulfide (5.8-5.6),neutral x neutral (5.8-5.6),neutral x positive (5.8-5.6),neutral x sulfide (5.8-5.6),positive x positive (5.8-5.6),positive x sulfide (5.8-5.6),sulfide x sulfide (5.8-5.6),acceptor x acceptor (5.6-5.4),acceptor x donor (5.6-5.4),acceptor x aromatic (5.6-5.4),acceptor x hydrophobic (5.6-5.4),acceptor x negative (5.6-5.4),acceptor x neutral (5.6-5.4),acceptor x positive (5.6-5.4),acceptor x sulfide (5.6-5.4),donor x donor (5.6-5.4),donor x aromatic (5.6-5.4),donor x hydrophobic (5.6-5.4),donor x negative (5.6-5.4),donor x neutral (5.6-5.4),donor x positive (5.6-5.4),donor x sulfide (5.6-5.4),aromatic x aromatic (5.6-5.4),aromatic x hydrophobic (5.6-5.4),aromatic x negative (5.6-5.4),aromatic x neutral (5.6-5.4),aromatic x positive (5.6-5.4),aromatic x sulfide (5.6-5.4),hydrophobic x hydrophobic (5.6-5.4),hydrophobic x negative (5.6-5.4),hydrophobic x neutral (5.6-5.4),hydrophobic x positive (5.6-5.4),hydrophobic x sulfide (5.6-5.4),negative x negative (5.6-5.4),negative x neutral (5.6-5.4),negative x positive (5.6-5.4),negative x sulfide (5.6-5.4),neutral x neutral (5.6-5.4),neutral x positive (5.6-5.4),neutral x sulfide (5.6-5.4),positive x positive (5.6-5.4),positive x sulfide (5.6-5.4),sulfide x sulfide (5.6-5.4),acceptor x acceptor (5.4-5.2),acceptor x donor (5.4-5.2),acceptor x aromatic (5.4-5.2),acceptor x hydrophobic (5.4-5.2),acceptor x negative (5.4-5.2),acceptor x neutral (5.4-5.2),acceptor x positive (5.4-5.2),acceptor x sulfide (5.4-5.2),donor x donor (5.4-5.2),donor x aromatic (5.4-5.2),donor x hydrophobic (5.4-5.2),donor x negative (5.4-5.2),donor x neutral (5.4-5.2),donor x positive (5.4-5.2),donor x sulfide (5.4-5.2),aromatic x aromatic (5.4-5.2),aromatic x hydrophobic (5.4-5.2),aromatic x negative (5.4-5.2),aromatic x neutral (5.4-5.2),aromatic x positive (5.4-5.2),aromatic x sulfide (5.4-5.2),hydrophobic x hydrophobic (5.4-5.2),hydrophobic x negative (5.4-5.2),hydrophobic x neutral (5.4-5.2),hydrophobic x positive (5.4-5.2),hydrophobic x sulfide (5.4-5.2),negative x negative (5.4-5.2),negative x neutral (5.4-5.2),negative x positive (5.4-5.2),negative x sulfide (5.4-5.2),neutral x neutral (5.4-5.2),neutral x positive (5.4-5.2),neutral x sulfide (5.4-5.2),positive x positive (5.4-5.2),positive x sulfide (5.4-5.2),sulfide x sulfide (5.4-5.2),acceptor x acceptor (5.2-5.0),acceptor x donor (5.2-5.0),acceptor x aromatic (5.2-5.0),acceptor x hydrophobic (5.2-5.0),acceptor x negative (5.2-5.0),acceptor x neutral (5.2-5.0),acceptor x positive (5.2-5.0),acceptor x sulfide (5.2-5.0),donor x donor (5.2-5.0),donor x aromatic (5.2-5.0),donor x hydrophobic (5.2-5.0),donor x negative (5.2-5.0),donor x neutral (5.2-5.0),donor x positive (5.2-5.0),donor x sulfide (5.2-5.0),aromatic x aromatic (5.2-5.0),aromatic x hydrophobic (5.2-5.0),aromatic x negative (5.2-5.0),aromatic x neutral (5.2-5.0),aromatic x positive (5.2-5.0),aromatic x sulfide (5.2-5.0),hydrophobic x hydrophobic (5.2-5.0),hydrophobic x negative (5.2-5.0),hydrophobic x neutral (5.2-5.0),hydrophobic x positive (5.2-5.0),hydrophobic x sulfide (5.2-5.0),negative x negative (5.2-5.0),negative x neutral (5.2-5.0),negative x positive (5.2-5.0),negative x sulfide (5.2-5.0),neutral x neutral (5.2-5.0),neutral x positive (5.2-5.0),neutral x sulfide (5.2-5.0),positive x positive (5.2-5.0),positive x sulfide (5.2-5.0),sulfide x sulfide (5.2-5.0),acceptor x acceptor (5.0-4.8),acceptor x donor (5.0-4.8),acceptor x aromatic (5.0-4.8),acceptor x hydrophobic (5.0-4.8),acceptor x negative (5.0-4.8),acceptor x neutral (5.0-4.8),acceptor x positive (5.0-4.8),acceptor x sulfide (5.0-4.8),donor x donor (5.0-4.8),donor x aromatic (5.0-4.8),donor x hydrophobic (5.0-4.8),donor x negative (5.0-4.8),donor x neutral (5.0-4.8),donor x positive (5.0-4.8),donor x sulfide (5.0-4.8),aromatic x aromatic (5.0-4.8),aromatic x hydrophobic (5.0-4.8),aromatic x negative (5.0-4.8),aromatic x neutral (5.0-4.8),aromatic x positive (5.0-4.8),aromatic x sulfide (5.0-4.8),hydrophobic x hydrophobic (5.0-4.8),hydrophobic x negative (5.0-4.8),hydrophobic x neutral (5.0-4.8),hydrophobic x positive (5.0-4.8),hydrophobic x sulfide (5.0-4.8),negative x negative (5.0-4.8),negative x neutral (5.0-4.8),negative x positive (5.0-4.8),negative x sulfide (5.0-4.8),neutral x neutral (5.0-4.8),neutral x positive (5.0-4.8),neutral x sulfide (5.0-4.8),positive x positive (5.0-4.8),positive x sulfide (5.0-4.8),sulfide x sulfide (5.0-4.8),acceptor x acceptor (4.8-4.6),acceptor x donor (4.8-4.6),acceptor x aromatic (4.8-4.6),acceptor x hydrophobic (4.8-4.6),acceptor x negative (4.8-4.6),acceptor x neutral (4.8-4.6),acceptor x positive (4.8-4.6),acceptor x sulfide (4.8-4.6),donor x donor (4.8-4.6),donor x aromatic (4.8-4.6),donor x hydrophobic (4.8-4.6),donor x negative (4.8-4.6),donor x neutral (4.8-4.6),donor x positive (4.8-4.6),donor x sulfide (4.8-4.6),aromatic x aromatic (4.8-4.6),aromatic x hydrophobic (4.8-4.6),aromatic x negative (4.8-4.6),aromatic x neutral (4.8-4.6),aromatic x positive (4.8-4.6),aromatic x sulfide (4.8-4.6),hydrophobic x hydrophobic (4.8-4.6),hydrophobic x negative (4.8-4.6),hydrophobic x neutral (4.8-4.6),hydrophobic x positive (4.8-4.6),hydrophobic x sulfide (4.8-4.6),negative x negative (4.8-4.6),negative x neutral (4.8-4.6),negative x positive (4.8-4.6),negative x sulfide (4.8-4.6),neutral x neutral (4.8-4.6),neutral x positive (4.8-4.6),neutral x sulfide (4.8-4.6),positive x positive (4.8-4.6),positive x sulfide (4.8-4.6),sulfide x sulfide (4.8-4.6),acceptor x acceptor (4.6-4.4),acceptor x donor (4.6-4.4),acceptor x aromatic (4.6-4.4),acceptor x hydrophobic (4.6-4.4),acceptor x negative (4.6-4.4),acceptor x neutral (4.6-4.4),acceptor x positive (4.6-4.4),acceptor x sulfide (4.6-4.4),donor x donor (4.6-4.4),donor x aromatic (4.6-4.4),donor x hydrophobic (4.6-4.4),donor x negative (4.6-4.4),donor x neutral (4.6-4.4),donor x positive (4.6-4.4),donor x sulfide (4.6-4.4),aromatic x aromatic (4.6-4.4),aromatic x hydrophobic (4.6-4.4),aromatic x negative (4.6-4.4),aromatic x neutral (4.6-4.4),aromatic x positive (4.6-4.4),aromatic x sulfide (4.6-4.4),hydrophobic x hydrophobic (4.6-4.4),hydrophobic x negative (4.6-4.4),hydrophobic x neutral (4.6-4.4),hydrophobic x positive (4.6-4.4),hydrophobic x sulfide (4.6-4.4),negative x negative (4.6-4.4),negative x neutral (4.6-4.4),negative x positive (4.6-4.4),negative x sulfide (4.6-4.4),neutral x neutral (4.6-4.4),neutral x positive (4.6-4.4),neutral x sulfide (4.6-4.4),positive x positive (4.6-4.4),positive x sulfide (4.6-4.4),sulfide x sulfide (4.6-4.4),acceptor x acceptor (4.4-4.2),acceptor x donor (4.4-4.2),acceptor x aromatic (4.4-4.2),acceptor x hydrophobic (4.4-4.2),acceptor x negative (4.4-4.2),acceptor x neutral (4.4-4.2),acceptor x positive (4.4-4.2),acceptor x sulfide (4.4-4.2),donor x donor (4.4-4.2),donor x aromatic (4.4-4.2),donor x hydrophobic (4.4-4.2),donor x negative (4.4-4.2),donor x neutral (4.4-4.2),donor x positive (4.4-4.2),donor x sulfide

(4.4-4.2),aromatic x aromatic (4.4-4.2),aromatic x hydrophobic (4.4-4.2),aromatic x negative (4.4-4.2),aromatic x neutral (4.4-4.2),aromatic x positive (4.4-4.2),aromatic x sulfide (4.4-4.2),hydrophobic x hydrophobic (4.4-4.2),hydrophobic x negative (4.4-4.2),hydrophobic x neutral (4.4-4.2),hydrophobic x positive (4.4-4.2),hydrophobic x sulfide (4.4-4.2),negative x negative (4.4-4.2),negative x neutral (4.4-4.2),negative x positive (4.4-4.2),negative x sulfide (4.4-4.2),neutral x neutral (4.4-4.2),neutral x positive (4.4-4.2),neutral x sulfide (4.4-4.2),positive x positive (4.4-4.2),positive x sulfide (4.4-4.2),sulfide x sulfide (4.4-4.2),acceptor x acceptor (4.2-4.0),acceptor x donor (4.2-4.0),acceptor x aromatic (4.2-4.0),acceptor x hydrophobic (4.2-4.0),acceptor x negative (4.2-4.0),acceptor x neutral (4.2-4.0),acceptor x positive (4.2-4.0),acceptor x sulfide (4.2-4.0),donor x donor (4.2-4.0),donor x aromatic (4.2-4.0),donor x hydrophobic (4.2-4.0),donor x negative (4.2-4.0),donor x neutral (4.2-4.0),donor x positive (4.2-4.0),donor x sulfide (4.2-4.0),aromatic x aromatic (4.2-4.0),aromatic x hydrophobic (4.2-4.0),aromatic x negative (4.2-4.0),aromatic x neutral (4.2-4.0),aromatic x positive (4.2-4.0),aromatic x sulfide (4.2-4.0),hydrophobic x hydrophobic (4.2-4.0),hydrophobic x negative (4.2-4.0),hydrophobic x neutral (4.2-4.0),hydrophobic x positive (4.2-4.0),hydrophobic x sulfide (4.2-4.0),negative x negative (4.2-4.0),negative x neutral (4.2-4.0),negative x positive (4.2-4.0),negative x sulfide (4.2-4.0),neutral x neutral (4.2-4.0),neutral x positive (4.2-4.0),neutral x sulfide (4.2-4.0),positive x positive (4.2-4.0),positive x sulfide (4.2-4.0),sulfide x sulfide (4.2-4.0),acceptor x acceptor (4.0-3.8),acceptor x donor (4.0-3.8),acceptor x aromatic (4.0-3.8),acceptor x hydrophobic (4.0-3.8),acceptor x negative (4.0-3.8),acceptor x neutral (4.0-3.8),acceptor x positive (4.0-3.8),acceptor x sulfide (4.0-3.8),donor x donor (4.0-3.8),donor x aromatic (4.0-3.8),donor x hydrophobic (4.0-3.8),donor x negative (4.0-3.8),donor x neutral (4.0-3.8),donor x positive (4.0-3.8),donor x sulfide (4.0-3.8),aromatic x aromatic (4.0-3.8),aromatic x hydrophobic (4.0-3.8),aromatic x negative (4.0-3.8),aromatic x neutral (4.0-3.8),aromatic x positive (4.0-3.8),aromatic x sulfide (4.0-3.8),hydrophobic x hydrophobic (4.0-3.8),hydrophobic x negative (4.0-3.8),hydrophobic x neutral (4.0-3.8),hydrophobic x positive (4.0-3.8),hydrophobic x sulfide (4.0-3.8),negative x negative (4.0-3.8),negative x neutral (4.0-3.8),negative x positive (4.0-3.8),negative x sulfide (4.0-3.8),neutral x neutral (4.0-3.8),neutral x positive (4.0-3.8),neutral x sulfide (4.0-3.8),positive x positive (4.0-3.8),positive x sulfide (4.0-3.8),sulfide x sulfide (4.0-3.8),acceptor x acceptor (3.8-3.6),acceptor x donor (3.8-3.6),acceptor x aromatic (3.8-3.6),acceptor x hydrophobic (3.8-3.6),acceptor x negative (3.8-3.6),acceptor x neutral (3.8-3.6),acceptor x positive (3.8-3.6),acceptor x sulfide (3.8-3.6),donor x donor (3.8-3.6),donor x aromatic (3.8-3.6),donor x hydrophobic (3.8-3.6),donor x negative (3.8-3.6),donor x neutral (3.8-3.6),donor x positive (3.8-3.6),donor x sulfide (3.8-3.6),aromatic x aromatic (3.8-3.6),aromatic x hydrophobic (3.8-3.6),aromatic x negative (3.8-3.6),aromatic x neutral (3.8-3.6),aromatic x positive (3.8-3.6),aromatic x sulfide (3.8-3.6),hydrophobic x hydrophobic (3.8-3.6),hydrophobic x negative (3.8-3.6),hydrophobic x neutral (3.8-3.6),hydrophobic x positive (3.8-3.6),hydrophobic x sulfide (3.8-3.6),negative x negative (3.8-3.6),negative x neutral (3.8-3.6),negative x positive (3.8-3.6),negative x sulfide (3.8-3.6),neutral x neutral (3.8-3.6),neutral x positive (3.8-3.6),neutral x sulfide (3.8-3.6),positive x positive (3.8-3.6),positive x sulfide (3.8-3.6),sulfide x sulfide (3.8-3.6),acceptor x acceptor (3.6-3.4),acceptor x donor (3.6-3.4),acceptor x aromatic (3.6-3.4),acceptor x hydrophobic (3.6-3.4),acceptor x negative (3.6-3.4),acceptor x neutral (3.6-3.4),acceptor x positive (3.6-3.4),acceptor x sulfide (3.6-3.4),donor x donor (3.6-3.4),donor x aromatic (3.6-3.4),donor x hydrophobic (3.6-3.4),donor x negative (3.6-3.4),donor x neutral (3.6-3.4),donor x positive (3.6-3.4),donor x sulfide (3.6-3.4),aromatic x aromatic (3.6-3.4),aromatic x hydrophobic (3.6-3.4),aromatic x negative (3.6-3.4),aromatic x neutral (3.6-3.4),aromatic x positive (3.6-3.4),aromatic x sulfide (3.6-3.4),hydrophobic x hydrophobic (3.6-3.4),hydrophobic x negative (3.6-3.4),hydrophobic x neutral (3.6-3.4),hydrophobic x positive (3.6-3.4),hydrophobic x sulfide (3.6-3.4),negative x negative (3.6-3.4),negative x neutral (3.6-3.4),negative x positive (3.6-3.4),negative x sulfide (3.6-3.4),neutral x neutral (3.6-3.4),neutral x positive (3.6-3.4),neutral x sulfide (3.6-3.4),positive x positive (3.6-3.4),positive x sulfide (3.6-3.4),sulfide x sulfide (3.6-3.4),acceptor x acceptor (3.4-3.2),acceptor x donor (3.4-3.2),acceptor x aromatic (3.4-3.2),acceptor x hydrophobic (3.4-3.2),acceptor x negative (3.4-3.2),acceptor x neutral (3.4-3.2),acceptor x positive (3.4-3.2),acceptor x sulfide (3.4-3.2),donor x donor (3.4-3.2),donor x aromatic (3.4-3.2),donor x hydrophobic (3.4-3.2),donor x negative (3.4-3.2),donor x neutral (3.4-3.2),donor x positive (3.4-3.2),donor x sulfide (3.4-3.2),aromatic x aromatic (3.4-3.2),aromatic x hydrophobic (3.4-3.2),aromatic x negative (3.4-3.2),aromatic x neutral (3.4-3.2),aromatic x positive (3.4-3.2),aromatic x sulfide (3.4-3.2),hydrophobic x hydrophobic (3.4-3.2),hydrophobic x negative (3.4-3.2),hydrophobic x neutral (3.4-3.2),hydrophobic x positive (3.4-3.2),hydrophobic x sulfide (3.4-3.2),negative x negative (3.4-3.2),negative x neutral (3.4-3.2),negative x positive (3.4-3.2),negative x sulfide (3.4-3.2),neutral x neutral (3.4-3.2),neutral x positive (3.4-3.2),neutral x sulfide (3.4-3.2),positive x positive (3.4-3.2),positive x sulfide (3.4-3.2),sulfide x sulfide (3.4-3.2),acceptor x acceptor (3.2-3.0),acceptor x donor (3.2-3.0),acceptor x aromatic (3.2-3.0),acceptor x hydrophobic (3.2-3.0),acceptor x negative (3.2-3.0),acceptor x neutral (3.2-3.0),acceptor x positive (3.2-3.0),acceptor x sulfide (3.2-3.0),donor x donor (3.2-3.0),donor x aromatic (3.2-3.0),donor x hydrophobic (3.2-3.0),donor x negative (3.2-3.0),donor x neutral (3.2-3.0),donor x positive (3.2-3.0),donor x sulfide (3.2-3.0),aromatic x aromatic (3.2-3.0),aromatic x hydrophobic (3.2-3.0),aromatic x negative (3.2-3.0),aromatic x neutral (3.2-3.0),aromatic x positive (3.2-3.0),aromatic x sulfide (3.2-3.0),hydrophobic x hydrophobic (3.2-3.0),hydrophobic x negative (3.2-3.0),hydrophobic x neutral (3.2-3.0),hydrophobic x positive (3.2-3.0),hydrophobic x sulfide (3.2-3.0),negative x negative (3.2-3.0),negative x neutral (3.2-3.0),negative x positive (3.2-3.0),negative x sulfide (3.2-3.0),neutral x neutral (3.2-3.0),neutral x positive (3.2-3.0),neutral x sulfide (3.2-3.0),positive x positive (3.2-3.0),positive x sulfide (3.2-3.0),sulfide x sulfide (3.2-3.0),acceptor x acceptor (3.0-2.8),acceptor x donor (3.0-2.8),acceptor x aromatic (3.0-2.8),acceptor x hydrophobic (3.0-2.8),acceptor x negative (3.0-2.8),acceptor x neutral (3.0-2.8),acceptor x positive (3.0-2.8),acceptor x sulfide (3.0-2.8),donor x donor (3.0-2.8),donor x aromatic (3.0-2.8),donor x hydrophobic (3.0-2.8),donor x negative (3.0-2.8),donor x neutral (3.0-2.8),donor x positive (3.0-2.8),donor x sulfide (3.0-2.8),aromatic x aromatic (3.0-2.8),aromatic x hydrophobic (3.0-2.8),aromatic x negative (3.0-2.8),aromatic x neutral (3.0-2.8),aromatic x positive (3.0-2.8),aromatic x sulfide (3.0-2.8),hydrophobic x hydrophobic (3.0-2.8),hydrophobic x negative (3.0-2.8),hydrophobic x neutral (3.0-2.8),hydrophobic x positive (3.0-2.8),hydrophobic x sulfide (3.0-2.8),negative x negative (3.0-2.8),negative x neutral (3.0-2.8),negative x positive (3.0-2.8),negative x sulfide (3.0-2.8),neutral x neutral (3.0-2.8),neutral x positive (3.0-2.8),neutral x sulfide (3.0-2.8),positive x positive (3.0-2.8),positive x sulfide (3.0-2.8),sulfide x sulfide (3.0-2.8),acceptor x acceptor (2.8-2.6),acceptor x donor (2.8-2.6),acceptor x aromatic (2.8-2.6),acceptor x hydrophobic (2.8-2.6),acceptor x negative (2.8-2.6),acceptor x neutral (2.8-2.6),acceptor x positive (2.8-2.6),acceptor x sulfide (2.8-2.6),donor x donor (2.8-2.6),donor x aromatic (2.8-2.6),donor x hydrophobic (2.8-2.6),donor x negative (2.8-2.6),donor x neutral (2.8-2.6),donor x positive (2.8-2.6),donor x sulfide (2.8-2.6),aromatic x aromatic (2.8-2.6),aromatic x hydrophobic (2.8-2.6),aromatic x negative (2.8-2.6),aromatic x neutral (2.8-2.6),aromatic x positive (2.8-2.6),aromatic x sulfide (2.8-2.6),hydrophobic x hydrophobic (2.8-2.6),hydrophobic x negative (2.8-2.6),hydrophobic x neutral (2.8-2.6),hydrophobic x positive (2.8-2.6),hydrophobic x sulfide (2.8-2.6),negative x negative (2.8-2.6),negative x neutral (2.8-2.6),negative x positive (2.8-2.6),negative x sulfide (2.8-2.6),neutral x neutral (2.8-2.6),neutral x positive (2.8-2.6),neutral x sulfide (2.8-2.6),positive x positive (2.8-2.6),positive x sulfide (2.8-2.6),sulfide x sulfide (2.8-2.6),acceptor x acceptor (2.6-2.4),acceptor x donor (2.6-2.4),acceptor x aromatic (2.6-2.4),acceptor x hydrophobic (2.6-2.4),acceptor x negative (2.6-2.4),acceptor x neutral (2.6-2.4),acceptor x positive (2.6-2.4),acceptor x sulfide (2.6-2.4),donor x donor (2.6-2.4),donor x aromatic (2.6-2.4),donor x hydrophobic (2.6-2.4),donor x negative (2.6-2.4),donor x neutral (2.6-2.4),donor x positive (2.6-2.4),donor x sulfide (2.6-2.4),aromatic x aromatic (2.6-2.4),aromatic x hydrophobic (2.6-2.4),aromatic x negative (2.6-2.4),aromatic x neutral (2.6-2.4),aromatic x positive (2.6-2.4),aromatic x sulfide (2.6-2.4),hydrophobic x hydrophobic (2.6-2.4),hydrophobic x negative (2.6-2.4),hydrophobic x neutral (2.6-2.4),hydrophobic x positive (2.6-2.4),hydrophobic x sulfide (2.6-2.4),negative x negative (2.6-2.4),negative x neutral (2.6-2.4),negative x positive (2.6-2.4),negative x sulfide (2.6-2.4),neutral x neutral

(2.6-2.4),neutral x positive (2.6-2.4),neutral x sulfide (2.6-2.4),positive x positive (2.6-2.4),positive x sulfide (2.6-2.4),sulfide x sulfide (2.6-2.4),acceptor x acceptor (2.4-2.2),acceptor x donor (2.4-2.2),acceptor x aromatic (2.4-2.2),acceptor x hydrophobic (2.4-2.2),acceptor x negative (2.4-2.2),acceptor x neutral (2.4-2.2),acceptor x positive (2.4-2.2),acceptor x sulfide (2.4-2.2),donor x donor (2.4-2.2),donor x aromatic (2.4-2.2),donor x hydrophobic (2.4-2.2),donor x negative (2.4-2.2),donor x neutral (2.4-2.2),donor x positive (2.4-2.2),donor x sulfide (2.4-2.2),aromatic x aromatic (2.4-2.2),aromatic x hydrophobic (2.4-2.2),aromatic x negative (2.4-2.2),aromatic x neutral (2.4-2.2),aromatic x positive (2.4-2.2),aromatic x sulfide (2.4-2.2),hydrophobic x hydrophobic (2.4-2.2),hydrophobic x negative (2.4-2.2),hydrophobic x neutral (2.4-2.2),hydrophobic x positive (2.4-2.2),hydrophobic x sulfide (2.4-2.2),negative x negative (2.4-2.2),negative x neutral (2.4-2.2),negative x positive (2.4-2.2),negative x sulfide (2.4-2.2),neutral x neutral (2.4-2.2),neutral x positive (2.4-2.2),neutral x sulfide (2.4-2.2),positive x positive (2.4-2.2),positive x sulfide (2.4-2.2),sulfide x sulfide (2.4-2.2),acceptor x acceptor (2.2-2.0),acceptor x donor (2.2-2.0),acceptor x aromatic (2.2-2.0),acceptor x hydrophobic (2.2-2.0),acceptor x negative (2.2-2.0),acceptor x neutral (2.2-2.0),acceptor x positive (2.2-2.0),acceptor x sulfide (2.2-2.0),donor x donor (2.2-2.0),donor x aromatic (2.2-2.0),donor x hydrophobic (2.2-2.0),donor x negative (2.2-2.0),donor x neutral (2.2-2.0),donor x positive (2.2-2.0),donor x sulfide (2.2-2.0),aromatic x aromatic (2.2-2.0),aromatic x hydrophobic (2.2-2.0),aromatic x negative (2.2-2.0),aromatic x neutral (2.2-2.0),aromatic x positive (2.2-2.0),aromatic x sulfide (2.2-2.0),hydrophobic x hydrophobic (2.2-2.0),hydrophobic x negative (2.2-2.0),hydrophobic x neutral (2.2-2.0),hydrophobic x positive (2.2-2.0),hydrophobic x sulfide (2.2-2.0),negative x negative (2.2-2.0),negative x neutral (2.2-2.0),negative x positive (2.2-2.0),negative x sulfide (2.2-2.0),neutral x neutral (2.2-2.0),neutral x positive (2.2-2.0),neutral x sulfide (2.2-2.0),positive x positive (2.2-2.0),positive x sulfide (2.2-2.0),sulfide x sulfide (2.2-2.0),acceptor x acceptor (2.0-1.8),acceptor x donor (2.0-1.8),acceptor x aromatic (2.0-1.8),acceptor x hydrophobic (2.0-1.8),acceptor x negative (2.0-1.8),acceptor x neutral (2.0-1.8),acceptor x positive (2.0-1.8),acceptor x sulfide (2.0-1.8),donor x donor (2.0-1.8),donor x aromatic (2.0-1.8),donor x hydrophobic (2.0-1.8),donor x negative (2.0-1.8),donor x neutral (2.0-1.8),donor x positive (2.0-1.8),donor x sulfide (2.0-1.8),aromatic x aromatic (2.0-1.8),aromatic x hydrophobic (2.0-1.8),aromatic x negative (2.0-1.8),aromatic x neutral (2.0-1.8),aromatic x positive (2.0-1.8),aromatic x sulfide (2.0-1.8),hydrophobic x hydrophobic (2.0-1.8),hydrophobic x negative (2.0-1.8),hydrophobic x neutral (2.0-1.8),hydrophobic x positive (2.0-1.8),hydrophobic x sulfide (2.0-1.8),negative x negative (2.0-1.8),negative x neutral (2.0-1.8),negative x positive (2.0-1.8),negative x sulfide (2.0-1.8),neutral x neutral (2.0-1.8),neutral x positive (2.0-1.8),neutral x sulfide (2.0-1.8),positive x positive (2.0-1.8),positive x sulfide (2.0-1.8),sulfide x sulfide (2.0-1.8),acceptor x acceptor (1.8-1.6),acceptor x donor (1.8-1.6),acceptor x aromatic (1.8-1.6),acceptor x hydrophobic (1.8-1.6),acceptor x negative (1.8-1.6),acceptor x neutral (1.8-1.6),acceptor x positive (1.8-1.6),acceptor x sulfide (1.8-1.6),donor x donor (1.8-1.6),donor x aromatic (1.8-1.6),donor x hydrophobic (1.8-1.6),donor x negative (1.8-1.6),donor x neutral (1.8-1.6),donor x positive (1.8-1.6),donor x sulfide (1.8-1.6),aromatic x aromatic (1.8-1.6),aromatic x hydrophobic (1.8-1.6),aromatic x negative (1.8-1.6),aromatic x neutral (1.8-1.6),aromatic x positive (1.8-1.6),aromatic x sulfide (1.8-1.6),hydrophobic x hydrophobic (1.8-1.6),hydrophobic x negative (1.8-1.6),hydrophobic x neutral (1.8-1.6),hydrophobic x positive (1.8-1.6),hydrophobic x sulfide (1.8-1.6),negative x negative (1.8-1.6),negative x neutral (1.8-1.6),negative x positive (1.8-1.6),negative x sulfide (1.8-1.6),neutral x neutral (1.8-1.6),neutral x positive (1.8-1.6),neutral x sulfide (1.8-1.6),positive x positive (1.8-1.6),positive x sulfide (1.8-1.6),sulfide x sulfide (1.8-1.6),acceptor x acceptor (1.6-1.4),acceptor x donor (1.6-1.4),acceptor x aromatic (1.6-1.4),acceptor x hydrophobic (1.6-1.4),acceptor x negative (1.6-1.4),acceptor x neutral (1.6-1.4),acceptor x positive (1.6-1.4),acceptor x sulfide (1.6-1.4),donor x donor (1.6-1.4),donor x aromatic (1.6-1.4),donor x hydrophobic (1.6-1.4),donor x negative (1.6-1.4),donor x neutral (1.6-1.4),donor x positive (1.6-1.4),donor x sulfide (1.6-1.4),aromatic x aromatic (1.6-1.4),aromatic x hydrophobic (1.6-1.4),aromatic x negative (1.6-1.4),aromatic x neutral (1.6-1.4),aromatic x positive (1.6-1.4),aromatic x sulfide (1.6-1.4),hydrophobic x hydrophobic (1.6-1.4),hydrophobic x negative (1.6-1.4),hydrophobic x neutral (1.6-1.4),hydrophobic x positive (1.6-1.4),hydrophobic x sulfide (1.6-1.4),negative x negative (1.6-1.4),negative x neutral (1.6-1.4),negative x positive (1.6-1.4),negative x sulfide (1.6-1.4),neutral x neutral (1.6-1.4),neutral x positive (1.6-1.4),neutral x sulfide (1.6-1.4),positive x positive (1.6-1.4),positive x sulfide (1.6-1.4),sulfide x sulfide (1.6-1.4),acceptor x acceptor (1.4-1.2),acceptor x donor (1.4-1.2),acceptor x aromatic (1.4-1.2),acceptor x hydrophobic (1.4-1.2),acceptor x negative (1.4-1.2),acceptor x neutral (1.4-1.2),acceptor x positive (1.4-1.2),acceptor x sulfide (1.4-1.2),donor x donor (1.4-1.2),donor x aromatic (1.4-1.2),donor x hydrophobic (1.4-1.2),donor x negative (1.4-1.2),donor x neutral (1.4-1.2),donor x positive (1.4-1.2),donor x sulfide (1.4-1.2),aromatic x aromatic (1.4-1.2),aromatic x hydrophobic (1.4-1.2),aromatic x negative (1.4-1.2),aromatic x neutral (1.4-1.2),aromatic x positive (1.4-1.2),aromatic x sulfide (1.4-1.2),hydrophobic x hydrophobic (1.4-1.2),hydrophobic x negative (1.4-1.2),hydrophobic x neutral (1.4-1.2),hydrophobic x positive (1.4-1.2),hydrophobic x sulfide (1.4-1.2),negative x negative (1.4-1.2),negative x neutral (1.4-1.2),negative x positive (1.4-1.2),negative x sulfide (1.4-1.2),neutral x neutral (1.4-1.2),neutral x positive (1.4-1.2),neutral x sulfide (1.4-1.2),positive x positive (1.4-1.2),positive x sulfide (1.4-1.2),sulfide x sulfide (1.4-1.2),acceptor x acceptor (1.2-1.0),acceptor x donor (1.2-1.0),acceptor x aromatic (1.2-1.0),acceptor x hydrophobic (1.2-1.0),acceptor x negative (1.2-1.0),acceptor x neutral (1.2-1.0),acceptor x positive (1.2-1.0),acceptor x sulfide (1.2-1.0),donor x donor (1.2-1.0),donor x aromatic (1.2-1.0),donor x hydrophobic (1.2-1.0),donor x negative (1.2-1.0),donor x neutral (1.2-1.0),donor x positive (1.2-1.0),donor x sulfide (1.2-1.0),aromatic x aromatic (1.2-1.0),aromatic x hydrophobic (1.2-1.0),aromatic x negative (1.2-1.0),aromatic x neutral (1.2-1.0),aromatic x positive (1.2-1.0),aromatic x sulfide (1.2-1.0),hydrophobic x hydrophobic (1.2-1.0),hydrophobic x negative (1.2-1.0),hydrophobic x neutral (1.2-1.0),hydrophobic x positive (1.2-1.0),hydrophobic x sulfide (1.2-1.0),negative x negative (1.2-1.0),negative x neutral (1.2-1.0),negative x positive (1.2-1.0),negative x sulfide (1.2-1.0),neutral x neutral (1.2-1.0),neutral x positive (1.2-1.0),neutral x sulfide (1.2-1.0),positive x positive (1.2-1.0),positive x sulfide (1.2-1.0),sulfide x sulfide (1.2-1.0),acceptor x acceptor (1.0-0.8),acceptor x donor (1.0-0.8),acceptor x aromatic (1.0-0.8),acceptor x hydrophobic (1.0-0.8),acceptor x negative (1.0-0.8),acceptor x neutral (1.0-0.8),acceptor x positive (1.0-0.8),acceptor x sulfide (1.0-0.8),donor x donor (1.0-0.8),donor x aromatic (1.0-0.8),donor x hydrophobic (1.0-0.8),donor x negative (1.0-0.8),donor x neutral (1.0-0.8),donor x positive (1.0-0.8),donor x sulfide (1.0-0.8),aromatic x aromatic (1.0-0.8),aromatic x hydrophobic (1.0-0.8),aromatic x negative (1.0-0.8),aromatic x neutral (1.0-0.8),aromatic x positive (1.0-0.8),aromatic x sulfide (1.0-0.8),hydrophobic x hydrophobic (1.0-0.8),hydrophobic x negative (1.0-0.8),hydrophobic x neutral (1.0-0.8),hydrophobic x positive (1.0-0.8),hydrophobic x sulfide (1.0-0.8),negative x negative (1.0-0.8),negative x neutral (1.0-0.8),negative x positive (1.0-0.8),negative x sulfide (1.0-0.8),neutral x neutral (1.0-0.8),neutral x positive (1.0-0.8),neutral x sulfide (1.0-0.8),positive x positive (1.0-0.8),positive x sulfide (1.0-0.8),sulfide x sulfide (1.0-0.8),acceptor x acceptor (0.8-0.6),acceptor x donor (0.8-0.6),acceptor x aromatic (0.8-0.6),acceptor x hydrophobic (0.8-0.6),acceptor x negative (0.8-0.6),acceptor x neutral (0.8-0.6),acceptor x positive (0.8-0.6),acceptor x sulfide (0.8-0.6),donor x donor (0.8-0.6),donor x aromatic (0.8-0.6),donor x hydrophobic (0.8-0.6),donor x negative (0.8-0.6),donor x neutral (0.8-0.6),donor x positive (0.8-0.6),donor x sulfide (0.8-0.6),aromatic x aromatic (0.8-0.6),aromatic x hydrophobic (0.8-0.6),aromatic x negative (0.8-0.6),aromatic x neutral (0.8-0.6),aromatic x positive (0.8-0.6),aromatic x sulfide (0.8-0.6),hydrophobic x hydrophobic (0.8-0.6),hydrophobic x negative (0.8-0.6),hydrophobic x neutral (0.8-0.6),hydrophobic x positive (0.8-0.6),hydrophobic x sulfide (0.8-0.6),negative x negative (0.8-0.6),negative x neutral (0.8-0.6),negative x positive (0.8-0.6),negative x sulfide (0.8-0.6),neutral x neutral (0.8-0.6),neutral x positive (0.8-0.6),neutral x sulfide

(0.8-0.6),positive x positive (0.8-0.6),positive x sulfide (0.8-0.6),sulfide x sulfide (0.8-0.6),acceptor x acceptor (0.6-0.4),acceptor x donor (0.6-0.4),acceptor x aromatic (0.6-0.4),acceptor x hydrophobic (0.6-0.4),acceptor x negative (0.6-0.4),acceptor x neutral (0.6-0.4),acceptor x positive (0.6-0.4),acceptor x sulfide (0.6-0.4),donor x donor (0.6-0.4),donor x aromatic (0.6-0.4),donor x hydrophobic (0.6-0.4),donor x negative (0.6-0.4),donor x neutral (0.6-0.4),donor x positive (0.6-0.4),donor x sulfide (0.6-0.4),aromatic x aromatic (0.6-0.4),aromatic x hydrophobic (0.6-0.4),aromatic x negative (0.6-0.4),aromatic x neutral (0.6-0.4),aromatic x positive (0.6-0.4),aromatic x sulfide (0.6-0.4),hydrophobic x hydrophobic (0.6-0.4),hydrophobic x negative (0.6-0.4),hydrophobic x neutral (0.6-0.4),hydrophobic x positive (0.6-0.4),hydrophobic x sulfide (0.6-0.4),negative x negative (0.6-0.4),negative x neutral (0.6-0.4),negative x positive (0.6-0.4),negative x sulfide (0.6-0.4),neutral x neutral (0.6-0.4),neutral x positive (0.6-0.4),neutral x sulfide (0.6-0.4),positive x positive (0.6-0.4),positive x sulfide (0.6-0.4),sulfide x sulfide (0.6-0.4),acceptor x acceptor (0.4-0.2),acceptor x donor (0.4-0.2),acceptor x aromatic (0.4-0.2),acceptor x hydrophobic (0.4-0.2),acceptor x negative (0.4-0.2),acceptor x neutral (0.4-0.2),acceptor x positive (0.4-0.2),acceptor x sulfide (0.4-0.2),donor x donor (0.4-0.2),donor x aromatic (0.4-0.2),donor x hydrophobic (0.4-0.2),donor x negative (0.4-0.2),donor x neutral (0.4-0.2),donor x positive (0.4-0.2),donor x sulfide (0.4-0.2),aromatic x aromatic (0.4-0.2),aromatic x hydrophobic (0.4-0.2),aromatic x negative (0.4-0.2),aromatic x neutral (0.4-0.2),aromatic x positive (0.4-0.2),aromatic x sulfide (0.4-0.2),hydrophobic x hydrophobic (0.4-0.2),hydrophobic x negative (0.4-0.2),hydrophobic x neutral (0.4-0.2),hydrophobic x positive (0.4-0.2),hydrophobic x sulfide (0.4-0.2),negative x negative (0.4-0.2),negative x neutral (0.4-0.2),negative x positive (0.4-0.2),negative x sulfide (0.4-0.2),neutral x neutral (0.4-0.2),neutral x positive (0.4-0.2),neutral x sulfide (0.4-0.2),positive x positive (0.4-0.2),positive x sulfide (0.4-0.2),sulfide x sulfide (0.4-0.2),acceptor x acceptor (0.2-0.0),acceptor x donor (0.2-0.0),acceptor x aromatic (0.2-0.0),acceptor x hydrophobic (0.2-0.0),acceptor x negative (0.2-0.0),acceptor x neutral (0.2-0.0),acceptor x positive (0.2-0.0),acceptor x sulfide (0.2-0.0),donor x donor (0.2-0.0),donor x aromatic (0.2-0.0),donor x hydrophobic (0.2-0.0),donor x negative (0.2-0.0),donor x neutral (0.2-0.0),donor x positive (0.2-0.0),donor x sulfide (0.2-0.0),aromatic x aromatic (0.2-0.0),aromatic x hydrophobic (0.2-0.0),aromatic x negative (0.2-0.0),aromatic x neutral (0.2-0.0),aromatic x positive (0.2-0.0),aromatic x sulfide (0.2-0.0),hydrophobic x hydrophobic (0.2-0.0),hydrophobic x negative (0.2-0.0),hydrophobic x neutral (0.2-0.0),hydrophobic x positive (0.2-0.0),hydrophobic x sulfide (0.2-0.0),negative x negative (0.2-0.0),negative x neutral (0.2-0.0),negative x positive (0.2-0.0),negative x sulfide (0.2-0.0),neutral x neutral (0.2-0.0),neutral x positive (0.2-0.0),neutral x sulfide (0.2-0.0),positive x positive (0.2-0.0),positive x sulfide (0.2-0.0),sulfide x sulfide (0.2-0.0)

2 Case studies

2.1 Case study 1: five sequence clusters

We selected the five groups with the most elements for a case study in which we tried to reclassify them using the methodology based on structural signatures. Supplementary Figure 2 presents the consensus sequences with the most common amino acids found in the sequence clusters: S0, S1, S112, S151, and S162.

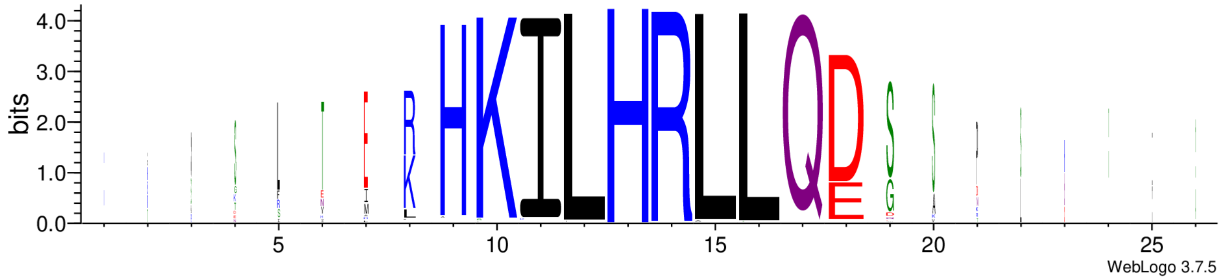

(A) S0

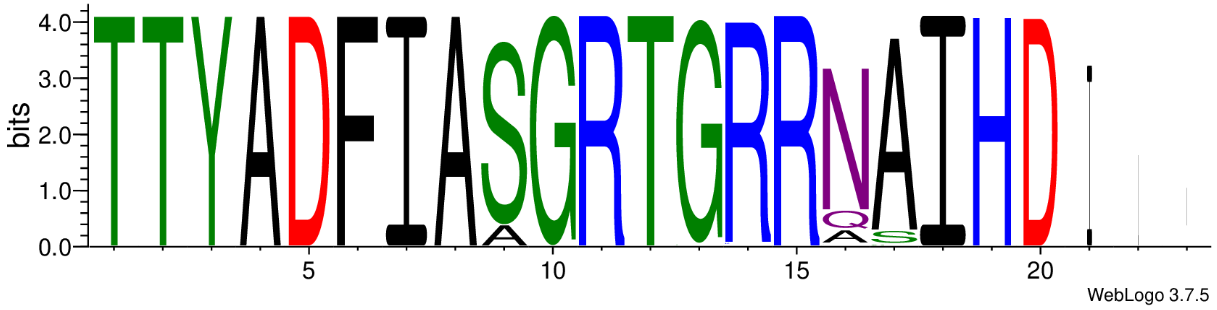

(B) S1

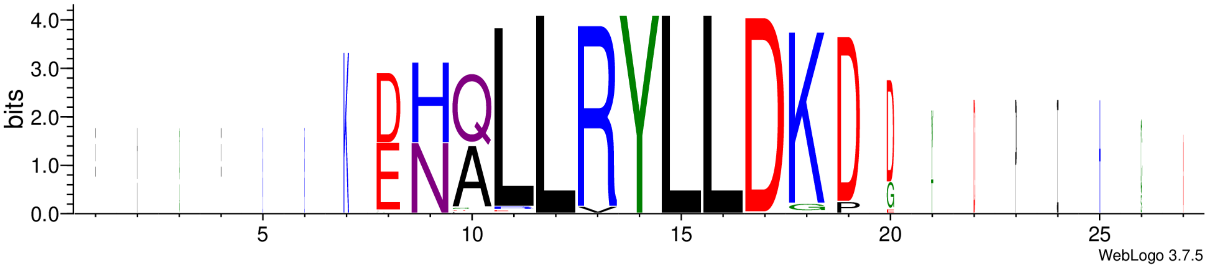

(C) S112

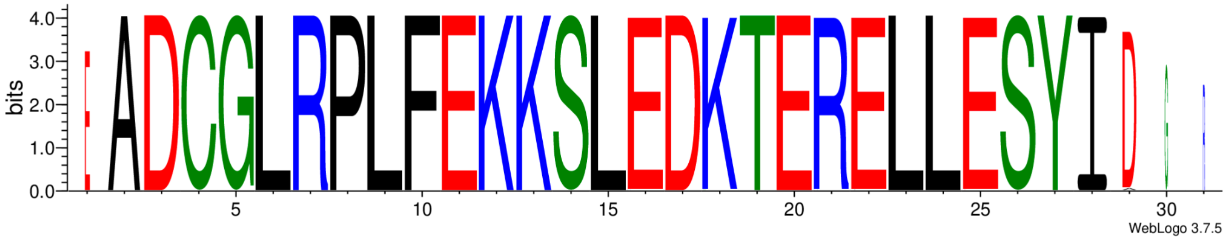

(D) S151

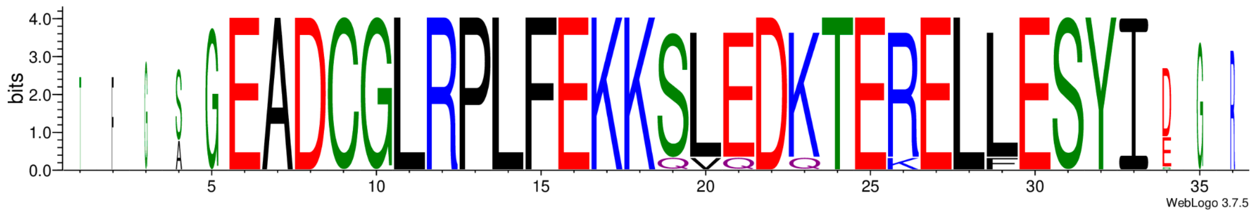

(E) S162

Supplementary Figure 2. Consensus sequences: the most common amino acids for each sequence cluster. Available at http://bioinfo.dcc.ufmg.br/propedia2/index.php/cluster/sequence/. Figures were generated using the Weblogo tool (https://weblogo.berkeley.edu/logo.cgi).

Supplementary Figure 3 presents the complete confusion matrix of the first case study for each machine learning algorithm: KNN, SVM, Logistic Regression, Decision tree, Random forest, Gradient Boosting, and Neural Network. The percentual values represent the number of correctly predicted samples for each class.

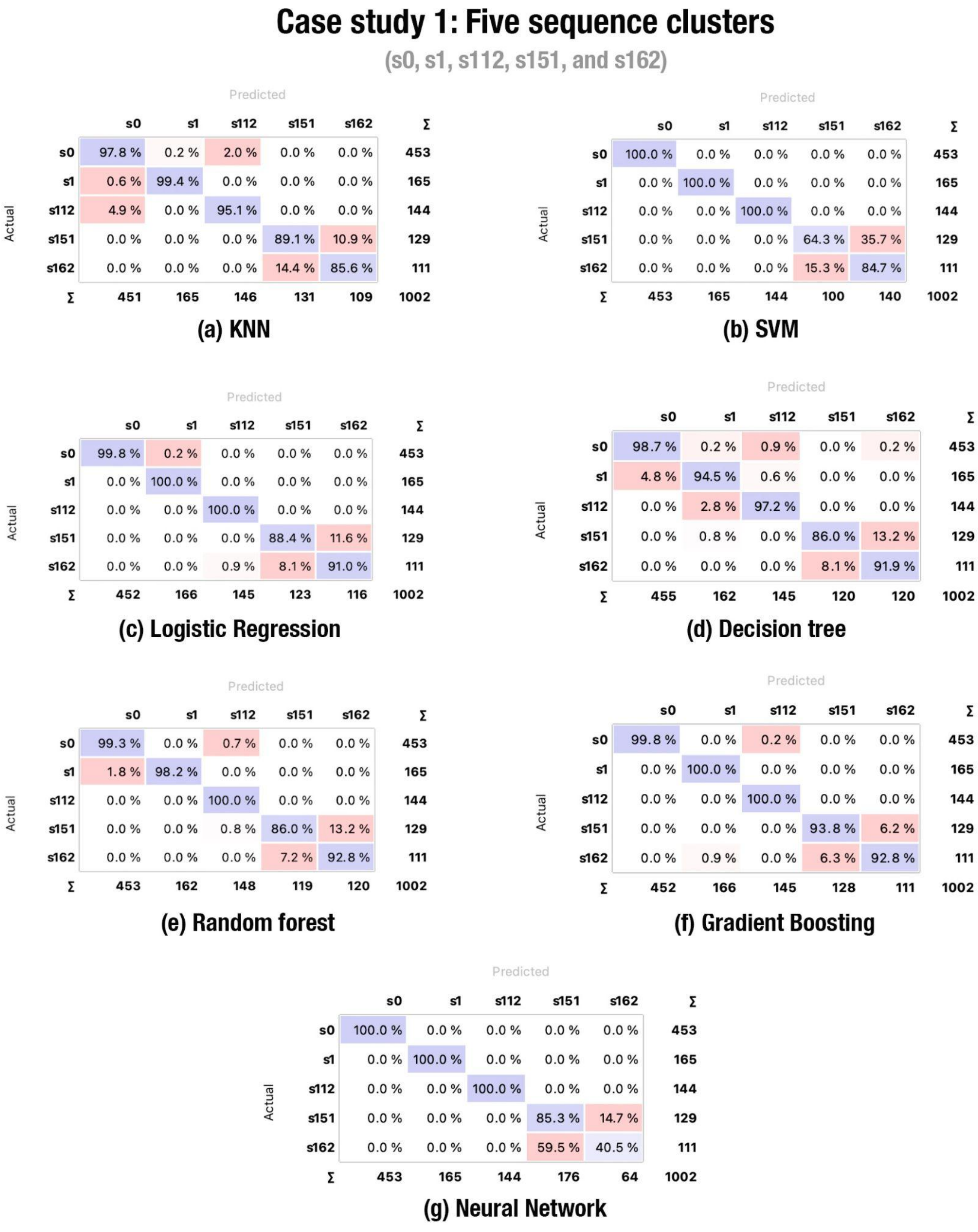

(a) KNN

Actual \ Predicted	s0	s1	s112	s151	s162	Σ
s0	97.8 %	0.2 %	2.0 %	0.0 %	0.0 %	453
s1	0.6 %	99.4 %	0.0 %	0.0 %	0.0 %	165
s112	4.9 %	0.0 %	95.1 %	0.0 %	0.0 %	144
s151	0.0 %	0.0 %	0.0 %	89.1 %	10.9 %	129
s162	0.0 %	0.0 %	0.0 %	14.4 %	85.6 %	111
Σ	451	165	146	131	109	1002

(b) SVM

Actual \ Predicted	s0	s1	s112	s151	s162	Σ
s0	100.0 %	0.0 %	0.0 %	0.0 %	0.0 %	453
s1	0.0 %	100.0 %	0.0 %	0.0 %	0.0 %	165
s112	0.0 %	0.0 %	100.0 %	0.0 %	0.0 %	144
s151	0.0 %	0.0 %	0.0 %	64.3 %	35.7 %	129
s162	0.0 %	0.0 %	0.0 %	15.3 %	84.7 %	111
Σ	453	165	144	100	140	1002

(c) Logistic Regression

Actual \ Predicted	s0	s1	s112	s151	s162	Σ
s0	99.8 %	0.2 %	0.0 %	0.0 %	0.0 %	453
s1	0.0 %	100.0 %	0.0 %	0.0 %	0.0 %	165
s112	0.0 %	0.0 %	100.0 %	0.0 %	0.0 %	144
s151	0.0 %	0.0 %	0.0 %	88.4 %	11.6 %	129
s162	0.0 %	0.0 %	0.9 %	8.1 %	91.0 %	111
Σ	452	166	145	123	116	1002

(d) Decision tree

Actual \ Predicted	s0	s1	s112	s151	s162	Σ
s0	98.7 %	0.2 %	0.9 %	0.0 %	0.2 %	453
s1	4.8 %	94.5 %	0.6 %	0.0 %	0.0 %	165
s112	0.0 %	2.8 %	97.2 %	0.0 %	0.0 %	144
s151	0.0 %	0.8 %	0.0 %	86.0 %	13.2 %	129
s162	0.0 %	0.0 %	0.0 %	8.1 %	91.9 %	111
Σ	455	162	145	120	120	1002

(e) Random forest

Actual \ Predicted	s0	s1	s112	s151	s162	Σ
s0	99.3 %	0.0 %	0.7 %	0.0 %	0.0 %	453
s1	1.8 %	98.2 %	0.0 %	0.0 %	0.0 %	165
s112	0.0 %	0.0 %	100.0 %	0.0 %	0.0 %	144
s151	0.0 %	0.0 %	0.8 %	86.0 %	13.2 %	129
s162	0.0 %	0.0 %	0.0 %	7.2 %	92.8 %	111
Σ	453	162	148	119	120	1002

(f) Gradient Boosting

Actual \ Predicted	s0	s1	s112	s151	s162	Σ
s0	99.8 %	0.0 %	0.2 %	0.0 %	0.0 %	453
s1	0.0 %	100.0 %	0.0 %	0.0 %	0.0 %	165
s112	0.0 %	0.0 %	100.0 %	0.0 %	0.0 %	144
s151	0.0 %	0.0 %	0.0 %	93.8 %	6.2 %	129
s162	0.0 %	0.9 %	0.0 %	6.3 %	92.8 %	111
Σ	452	166	145	128	111	1002

(g) Neural Network

Actual \ Predicted	s0	s1	s112	s151	s162	Σ
s0	100.0 %	0.0 %	0.0 %	0.0 %	0.0 %	453
s1	0.0 %	100.0 %	0.0 %	0.0 %	0.0 %	165
s112	0.0 %	0.0 %	100.0 %	0.0 %	0.0 %	144
s151	0.0 %	0.0 %	0.0 %	85.3 %	14.7 %	129
s162	0.0 %	0.0 %	0.0 %	59.5 %	40.5 %	111
Σ	453	165	144	176	64	1002

Supplementary Figure 3. Confusion matrix for case study 1. Figure generated using Orange Data Mining (https://orangedatamining.com/).

2.2 Case study 2: six Propedia clusters sub-datasets

In the second case study, we selected six groups based on their classification on the PDB database. The groups are enzyme, viral, plant, hormone, membrane, and antimicrobial. Supplementary Figure 4 presents the number of elements of each sub-dataset cluster used in case study 2.

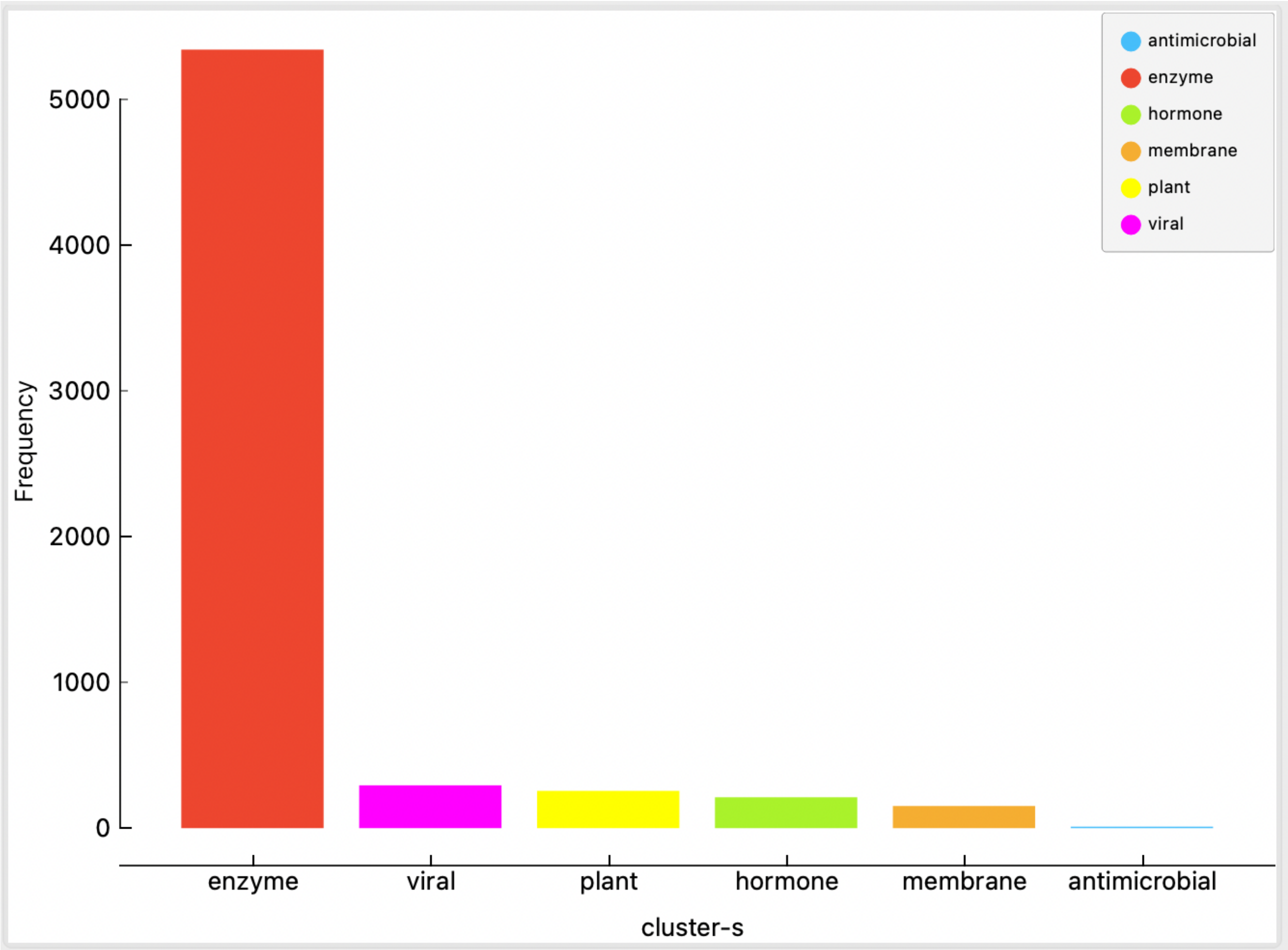

Supplementary Figure 4. Quantity of elements of each group of case study 2. Figure generated using Orange Data Mining.

Supplementary Figure 5 presents the complete confusion matrix of the second case study for each machine learning algorithm: KNN, SVM, Logistic Regression, Decision tree, Random forest, Gradient Boosting, and Neural Network. The percentual values represent the number of correctly predicted samples for each class.

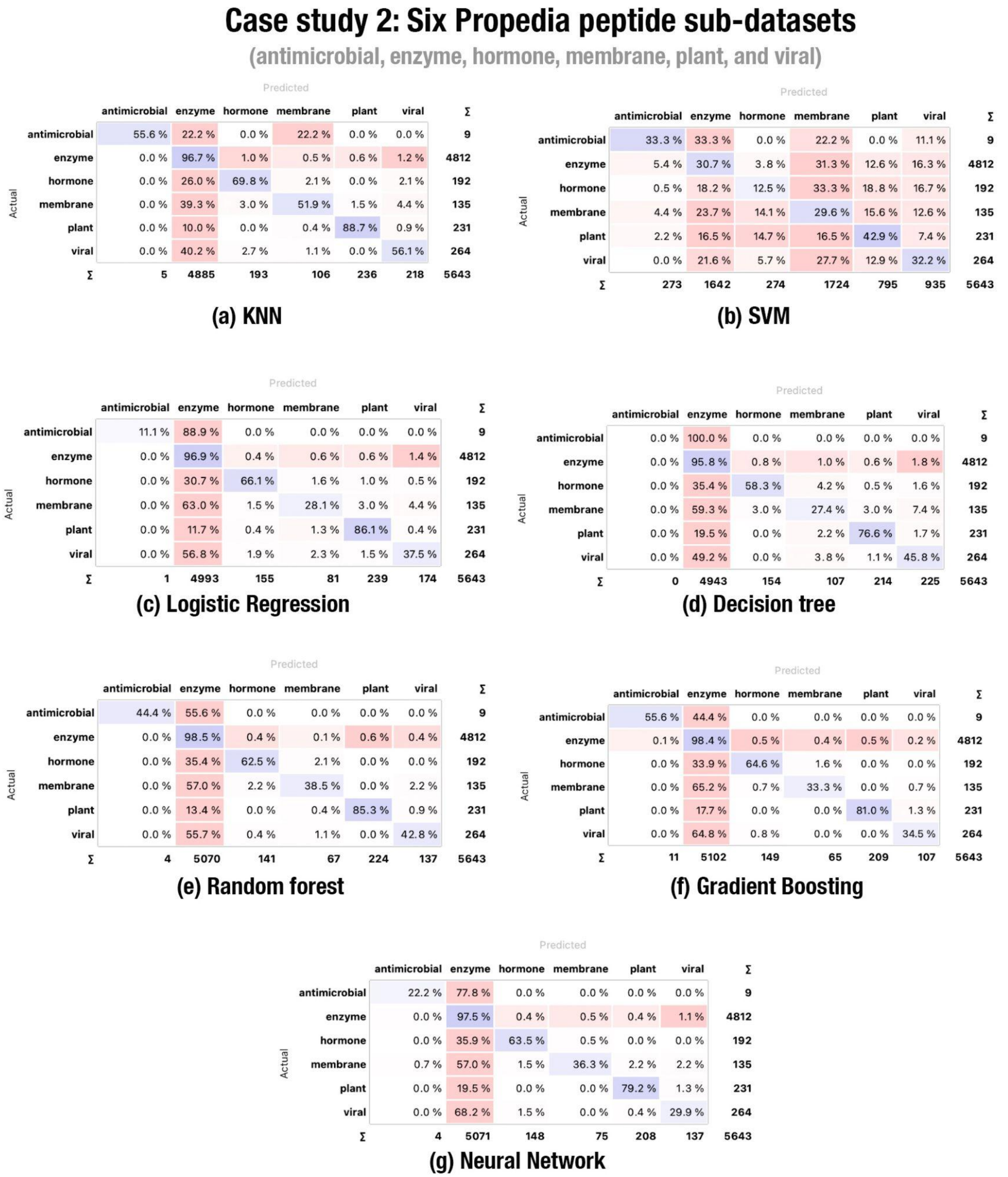

(a) KNN

	antimicrobial	enzyme	hormone	membrane	plant	viral	Σ
antimicrobial	55.6 %	22.2 %	0.0 %	22.2 %	0.0 %	0.0 %	9
enzyme	0.0 %	96.7 %	1.0 %	0.5 %	0.6 %	1.2 %	4812
hormone	0.0 %	26.0 %	69.8 %	2.1 %	0.0 %	2.1 %	192
membrane	0.0 %	39.3 %	3.0 %	51.9 %	1.5 %	4.4 %	135
plant	0.0 %	10.0 %	0.0 %	0.4 %	88.7 %	0.9 %	231
viral	0.0 %	40.2 %	2.7 %	1.1 %	0.0 %	56.1 %	264
Σ	5	4885	193	106	236	218	5643

(b) SVM

	antimicrobial	enzyme	hormone	membrane	plant	viral	Σ
antimicrobial	33.3 %	33.3 %	0.0 %	22.2 %	0.0 %	11.1 %	9
enzyme	5.4 %	30.7 %	3.8 %	31.3 %	12.6 %	16.3 %	4812
hormone	0.5 %	18.2 %	12.5 %	33.3 %	18.8 %	16.7 %	192
membrane	4.4 %	23.7 %	14.1 %	29.6 %	15.6 %	12.6 %	135
plant	2.2 %	16.5 %	14.7 %	16.5 %	42.9 %	7.4 %	231
viral	0.0 %	21.6 %	5.7 %	27.7 %	12.9 %	32.2 %	264
Σ	273	1642	274	1724	795	935	5643

(c) Logistic Regression

	antimicrobial	enzyme	hormone	membrane	plant	viral	Σ
antimicrobial	11.1 %	88.9 %	0.0 %	0.0 %	0.0 %	0.0 %	9
enzyme	0.0 %	96.9 %	0.4 %	0.6 %	0.6 %	1.4 %	4812
hormone	0.0 %	30.7 %	66.1 %	1.6 %	1.0 %	0.5 %	192
membrane	0.0 %	63.0 %	1.5 %	28.1 %	3.0 %	4.4 %	135
plant	0.0 %	11.7 %	0.4 %	1.3 %	86.1 %	0.4 %	231
viral	0.0 %	56.8 %	1.9 %	2.3 %	1.5 %	37.5 %	264
Σ	1	4993	155	81	239	174	5643

(d) Decision tree

	antimicrobial	enzyme	hormone	membrane	plant	viral	Σ
antimicrobial	0.0 %	100.0 %	0.0 %	0.0 %	0.0 %	0.0 %	9
enzyme	0.0 %	95.8 %	0.8 %	1.0 %	0.6 %	1.8 %	4812
hormone	0.0 %	35.4 %	58.3 %	4.2 %	0.5 %	1.6 %	192
membrane	0.0 %	59.3 %	3.0 %	27.4 %	3.0 %	7.4 %	135
plant	0.0 %	19.5 %	0.0 %	2.2 %	76.6 %	1.7 %	231
viral	0.0 %	49.2 %	0.0 %	3.8 %	1.1 %	45.8 %	264
Σ	0	4943	154	107	214	225	5643

(e) Random forest

	antimicrobial	enzyme	hormone	membrane	plant	viral	Σ
antimicrobial	44.4 %	55.6 %	0.0 %	0.0 %	0.0 %	0.0 %	9
enzyme	0.0 %	98.5 %	0.4 %	0.1 %	0.6 %	0.4 %	4812
hormone	0.0 %	35.4 %	62.5 %	2.1 %	0.0 %	0.0 %	192
membrane	0.0 %	57.0 %	2.2 %	38.5 %	0.0 %	2.2 %	135
plant	0.0 %	13.4 %	0.0 %	0.4 %	85.3 %	0.9 %	231
viral	0.0 %	55.7 %	0.4 %	1.1 %	0.0 %	42.8 %	264
Σ	4	5070	141	67	224	137	5643

(f) Gradient Boosting

	antimicrobial	enzyme	hormone	membrane	plant	viral	Σ
antimicrobial	55.6 %	44.4 %	0.0 %	0.0 %	0.0 %	0.0 %	9
enzyme	0.1 %	98.4 %	0.5 %	0.4 %	0.5 %	0.2 %	4812
hormone	0.0 %	33.9 %	64.6 %	1.6 %	0.0 %	0.0 %	192
membrane	0.0 %	65.2 %	0.7 %	33.3 %	0.0 %	0.7 %	135
plant	0.0 %	17.7 %	0.0 %	0.0 %	81.0 %	1.3 %	231
viral	0.0 %	64.8 %	0.8 %	0.0 %	0.0 %	34.5 %	264
Σ	11	5102	149	65	209	107	5643

(g) Neural Network

	antimicrobial	enzyme	hormone	membrane	plant	viral	Σ
antimicrobial	22.2 %	77.8 %	0.0 %	0.0 %	0.0 %	0.0 %	9
enzyme	0.0 %	97.5 %	0.4 %	0.5 %	0.4 %	1.1 %	4812
hormone	0.0 %	35.9 %	63.5 %	0.5 %	0.0 %	0.0 %	192
membrane	0.7 %	57.0 %	1.5 %	36.3 %	2.2 %	2.2 %	135
plant	0.0 %	19.5 %	0.0 %	0.0 %	79.2 %	1.3 %	231
viral	0.0 %	68.2 %	1.5 %	0.0 %	0.4 %	29.9 %	264
Σ	4	5071	148	75	208	137	5643

Supplementary Figure 5. Confusion matrix for case study 2. Figure generated using Orange Data Mining.

Lastly, we performed rank analyses of "info.gain" to evaluate the most important features for classifying previously selected groups. These analyses demonstrated that the acceptor-aromatic atom pairs and sulfide-sulfide atom pairs were the most important features for determining the groups (Supplementary Figure 5).

		#	Info.gain
1	N acceptor x aromatic (4.4-4.2)		0.112
2	N acceptor x aromatic (4.2-4.0)		0.111
3	N acceptor x aromatic (6.0-5.8)		0.110
4	N acceptor x aromatic (5.2-5.0)		0.110
5	N acceptor x aromatic (4.6-4.4)		0.110

(A) Infogain.

		#	Gai...tio
1	C sulfide x sulfide (6.0-5.8)	2	0.393
2	C sulfide x sulfide (5.8-5.6)	2	0.393
3	C sulfide x sulfide (5.2-5.0)	2	0.385
4	C sulfide x sulfide (5.6-5.4)	2	0.382
5	C sulfide x sulfide (6.4-6.2)	2	0.381

(B) Infogain (ratio)

Supplementary Figure 5. Most important features. Figure generated using Orange Data Mining (https://orangedatamining.com/).
