# Supplementary material for: Propedia v2.3: A novel representation approach for the peptide-protein interaction database using graph-based structural signatures
Source: Front Bioinform. 2023 Feb 16;3:1103103. doi: 10.3389/fbinf.2023.1103103 (PMC9978205; doi:10.3389/fbinf.2023.1103103)

ROC Analysis
Mon Jan 23 23, 18:56:00

Target class: antimicrobial
Costs: FP = 500, FN = 500
Target probability: 0.0 %

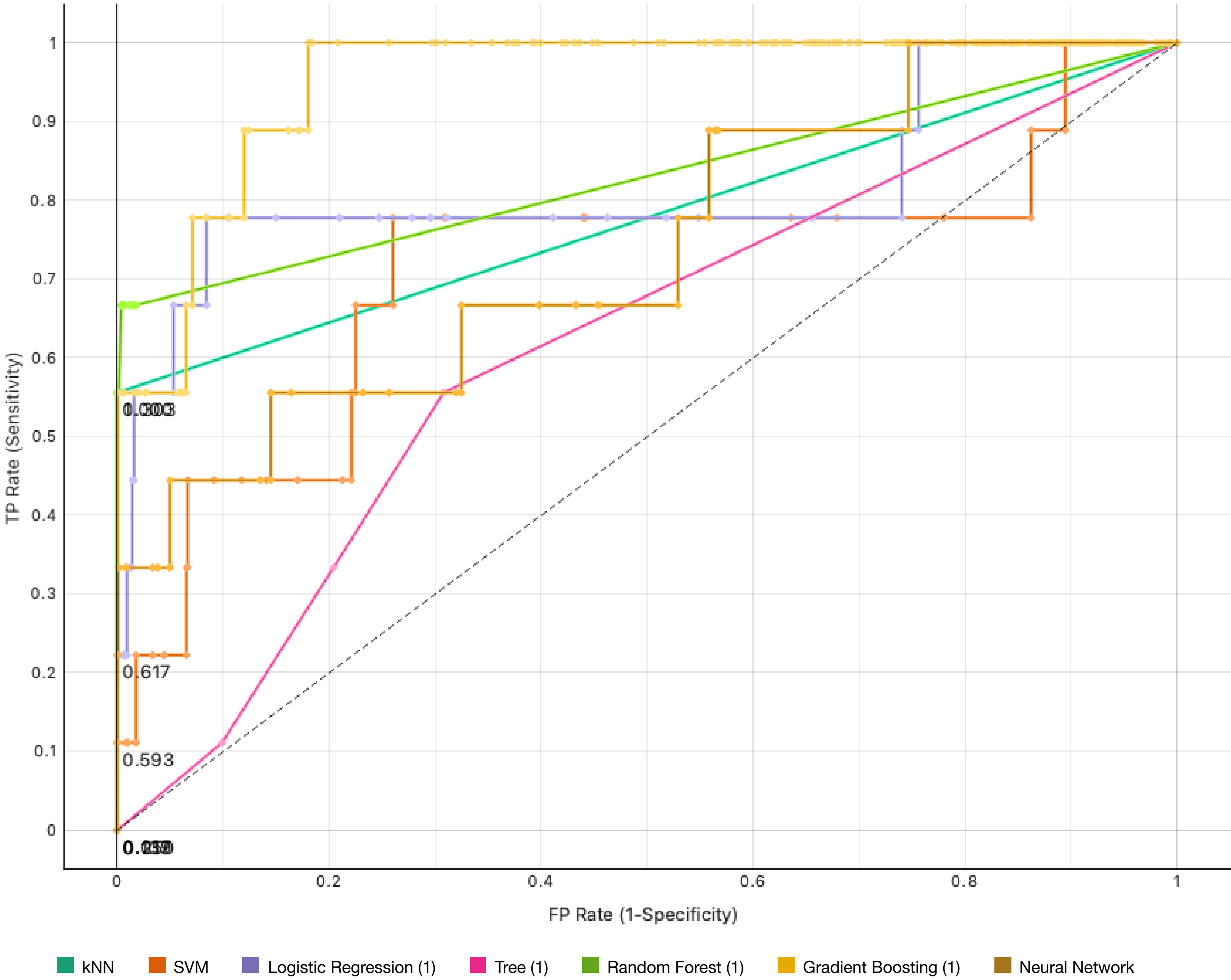

Target class: enzyme
Costs: FP = 500, FN = 500
Target probability: 85.0 %

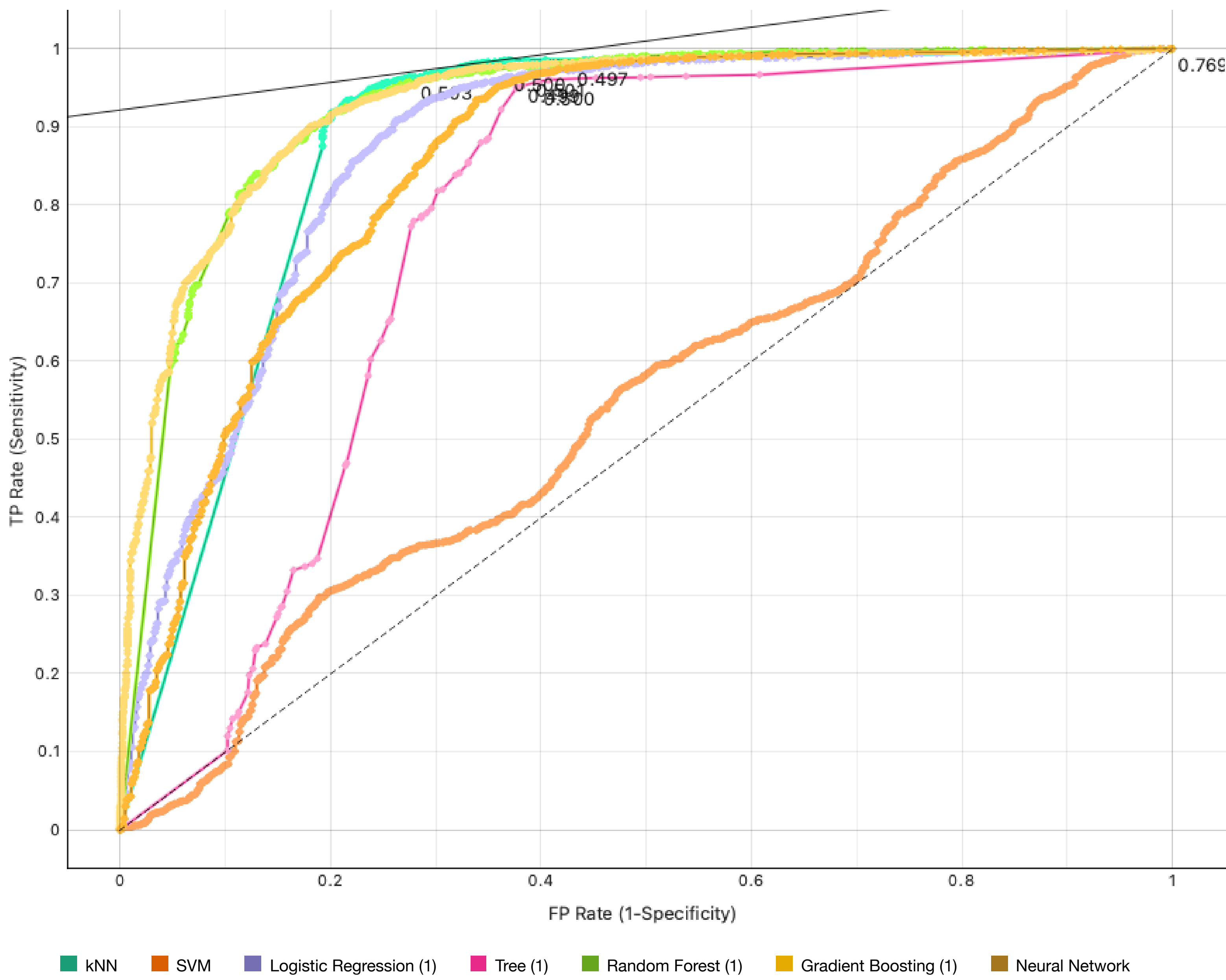

Target class: hormone
Costs: FP = 500, FN = 500
Target probability: 3.0 %

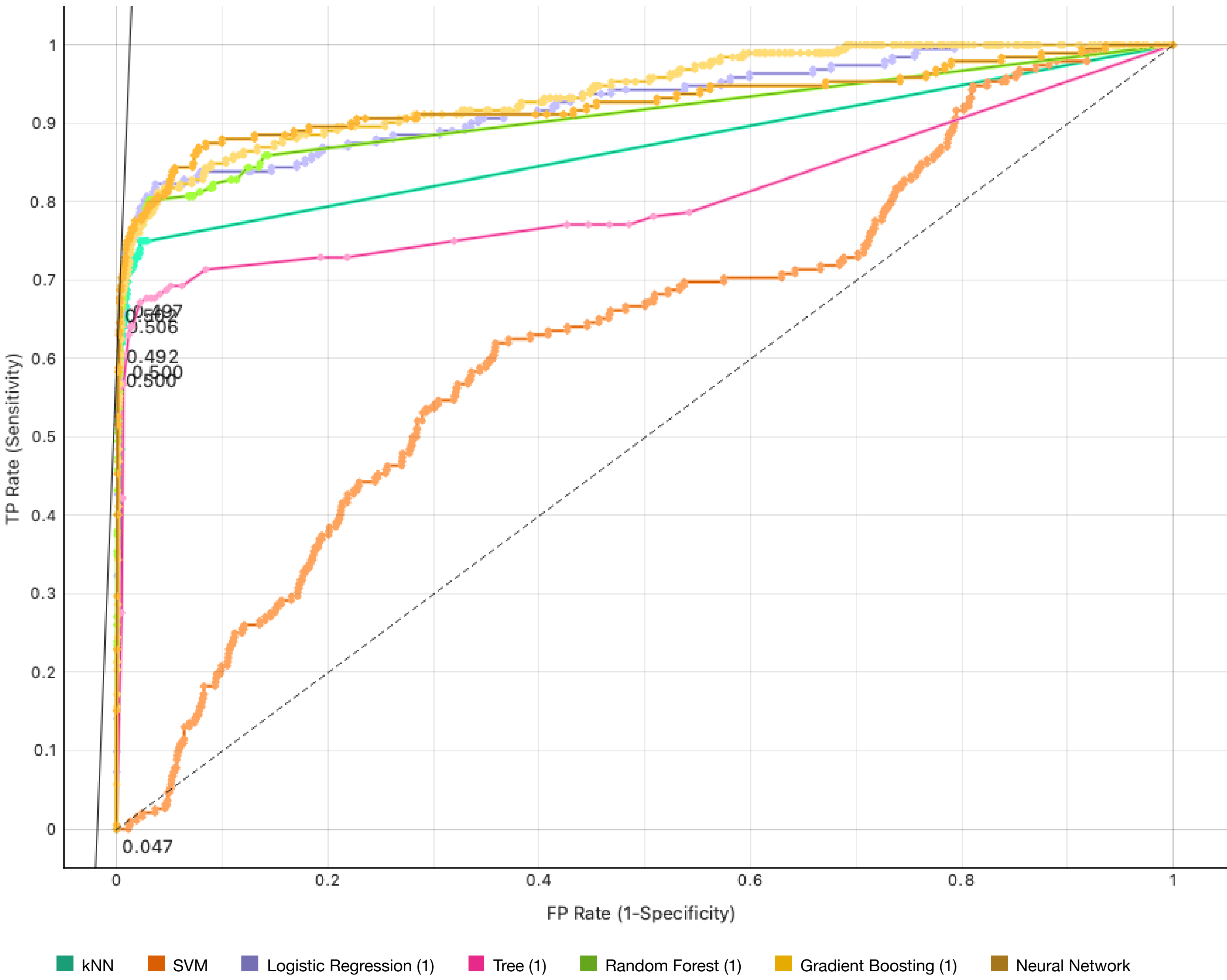

Target class: membrane
Costs: FP = 500, FN = 500
Target probability: 2.0 %

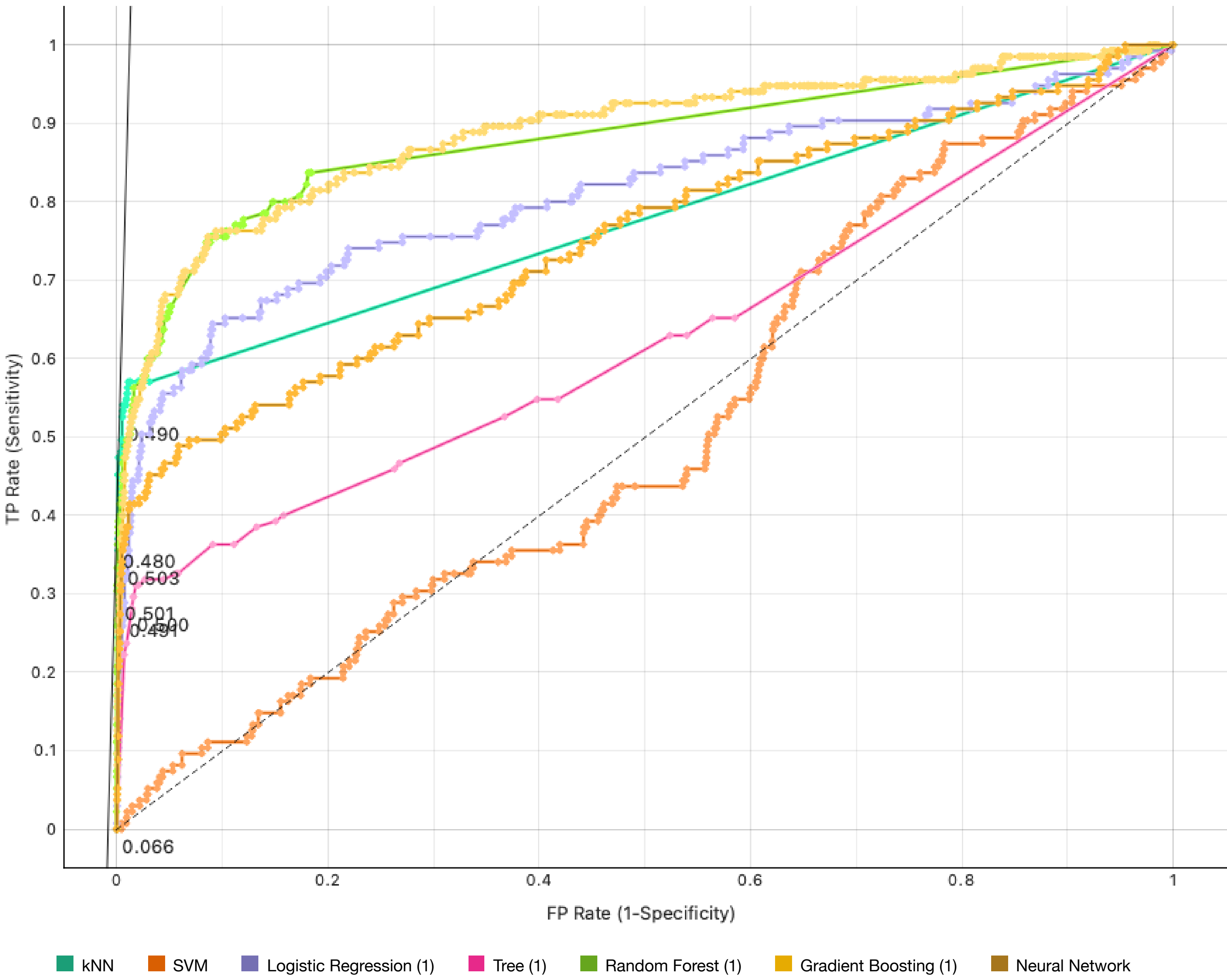

ROC Analysis Mon Jan 23 23, 18:56:22

Target class: plant
Costs: FP = 500, FN = 500
Target probability: 4.0 %

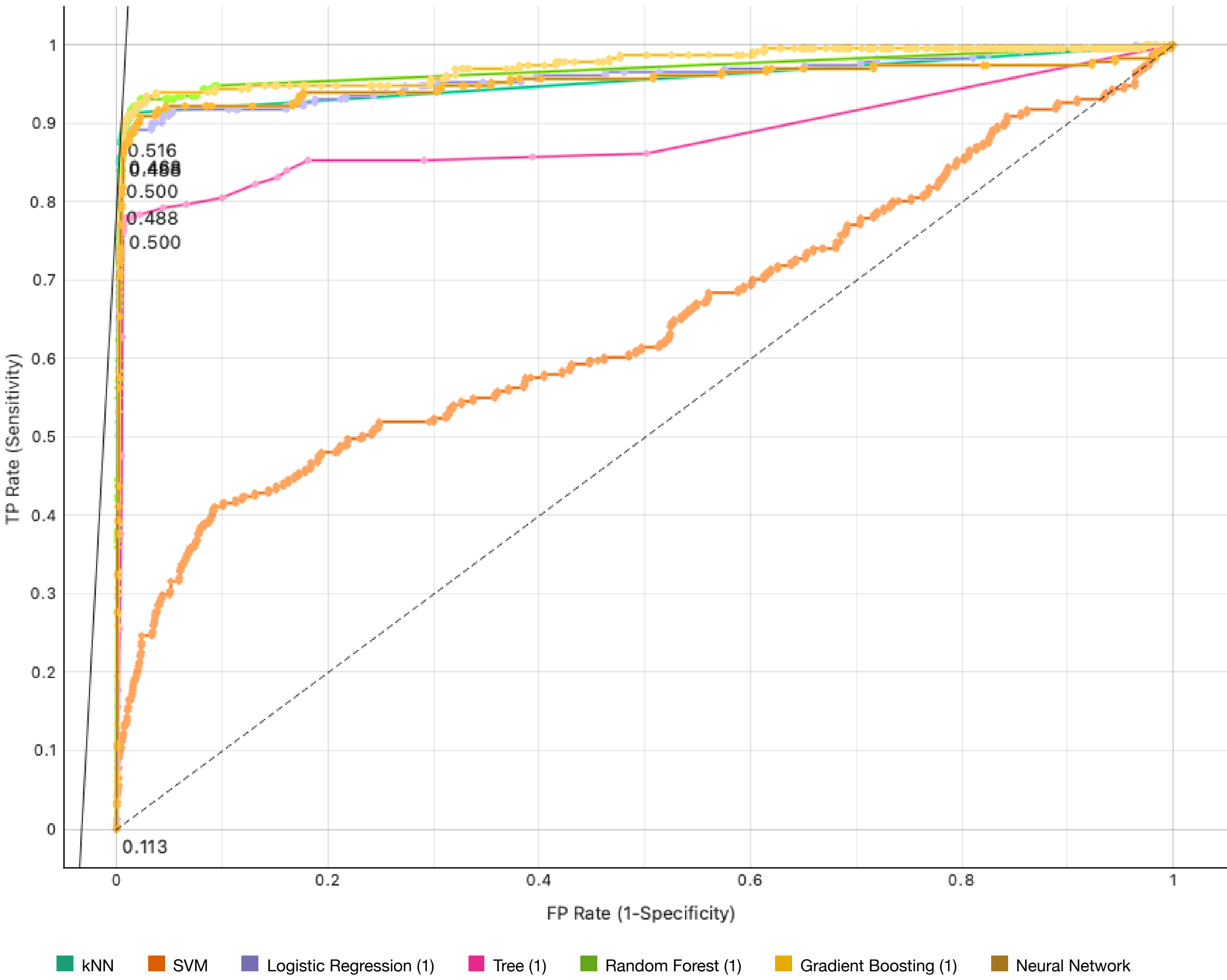

Target class: viral
Costs: FP = 500, FN = 500
Target probability: 5.0 %

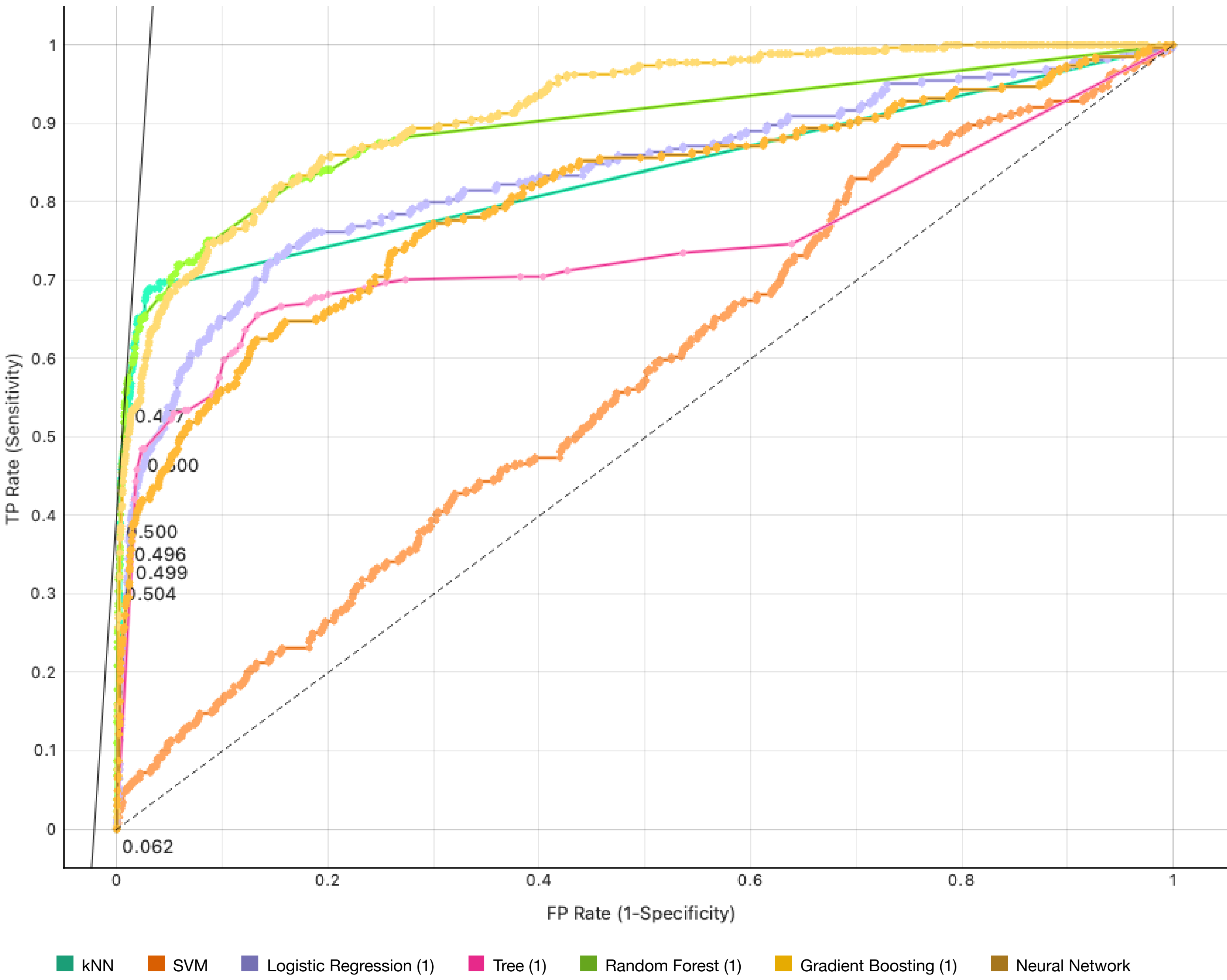

Supplement: Supplementary file 2 [file Image1.PDF]
